# The Role of Recent Admixture in Forming the Contemporary West Eurasian Genomic Landscape

**DOI:** 10.1016/j.cub.2015.08.007

**Published:** 2015-10-05

**Authors:** George B.J. Busby, Garrett Hellenthal, Francesco Montinaro, Sergio Tofanelli, Kazima Bulayeva, Igor Rudan, Tatijana Zemunik, Caroline Hayward, Draga Toncheva, Sena Karachanak-Yankova, Desislava Nesheva, Paolo Anagnostou, Francesco Cali, Francesca Brisighelli, Valentino Romano, Gerard Lefranc, Catherine Buresi, Jemni Ben Chibani, Amel Haj-Khelil, Sabri Denden, Rafal Ploski, Pawel Krajewski, Tor Hervig, Torolf Moen, Rene J. Herrera, James F. Wilson, Simon Myers, Cristian Capelli

**Affiliations:** 1 Department of Zoology, University of Oxford, South Parks Road, Oxford OX1 3PS, UK; 2 Wellcome Trust Centre for Human Genetics, University of Oxford, Roosevelt Drive, Oxford OX3 7BN, UK; 3 UCL Genetics Institute, University College London, Gower Street, London WC1E 6BT, UK; 4 Department of Biology, Università di Pisa, Via Ghini 13, 56126 Pisa, Italy; 5 N.I.Vavilov Institute of General Genetics, 3 Gubkin Street, Moscow 119991, Russia; 6 Centre for Global Health Research, Usher Institute of Population Heath Sciences and Informatics, University of Edinburgh, Teviot Place, Edinburgh EH8 9AG, UK; 7 Department of Medical Biology, School of Medicine Split, Soltanska 2, Split 21000, Croatia; 8 MRC Human Genetics Unit, Institute of Genetics and Molecular Medicine (IGMM), University of Edinburgh, Western General Hospital, Crewe Road, Edinburgh EH4 2XU, UK; 9 Department of Medical Genetics, National Human Genome Center, Medical University Sofia, Sofia 1431, Bulgaria; 10 Department of Environmental Biology, Università La Sapienza, Roma 00185, Italy; 11 Istituto Italiano di Antropologia, Roma 00185, Italy; 12 Laboratorio di Genetica Molecolare, IRCCS Associazione Oasi Maria SS, Troina 94018, Italy; 13 Forensic Genetics Laboratory, Institute of Legal Medicine, Università Cattolica del Sacro Cuore, Rome 00168, Italy; 14 Dipartimento di Fisica e Chimica, Università di Palermo, Palermo 90128, Italy; 15 Institute of Human Genetics, CNRS UPR 1142, and Montpellier University, Place Eugene Bataillon, 34095 Montpellier Cedex 5, France; 16 Laboratory of Biochemistry and Molecular Biology, Faculty of Pharmacy, 1 Avenue Avicenne, 5019 Monastir, Tunisia; 17 Department of Medical Genetics, Warsaw Medical University, 3c Pawinskiego Street, Warsaw 02-106, Poland; 18 Department of Forensic Medicine, Warsaw Medical University, 1 Oczki Street, Warsaw 02-007, Poland; 19 Department of Clinical Science, University of Bergen, Bergen 5021, Norway; 20 NTNU, Trondheim 7491, Norway; 21 Department of Human and Molecular Genetics, Florida International University, University Park, Miami, FL 33174, USA; 22 Department of Statistics, University of Oxford, South Parks Road, Oxford OX1 3TG, UK

## Abstract

Over the past few years, studies of DNA isolated from human fossils and archaeological remains have generated considerable novel insight into the history of our species. Several landmark papers have described the genomes of ancient humans across West Eurasia, demonstrating the presence of large-scale, dynamic population movements over the last 10,000 years, such that ancestry across present-day populations is likely to be a mixture of several ancient groups [1, 2, 3, 4, 5, 6, 7]. While these efforts are bringing the details of West Eurasian prehistory into increasing focus, studies aimed at understanding the processes behind the generation of the current West Eurasian genetic landscape have been limited by the number of populations sampled or have been either too regional or global in their outlook [8, 9, 10, 11]. Here, using recently described haplotype-based techniques [11], we present the results of a systematic survey of recent admixture history across Western Eurasia and show that admixture is a universal property across almost all groups. Admixture in all regions except North Western Europe involved the influx of genetic material from outside of West Eurasia, which we date to specific time periods. Within Northern, Western, and Central Europe, admixture tended to occur between local groups during the period 300 to 1200 CE. Comparisons of the genetic profiles of West Eurasians before and after admixture show that population movements within the last 1,500 years are likely to have maintained differentiation among groups. Our analysis provides a timeline of the gene flow events that have generated the contemporary genetic landscape of West Eurasia.

## Highlights

• Recent admixture events involved outside groups at the edges of West Eurasia
• Admixture within Europe tended to fall within the European Migration Period
• West Eurasian genetic structure today is likely to have been maintained by admixture

## Teaser

Using cutting edge statistical machinery, Busby et al. show that recent admixture is ubiquitous across West Eurasia, with the majority of populations showing evidence of population mixing. Dating of these admixture events demonstrates that the Medieval Migration Period was a key period in establishing the current West Eurasian genetic landscape.

## Results and Discussion

### The Genetic Structure of West Eurasia

**Figure 1:**
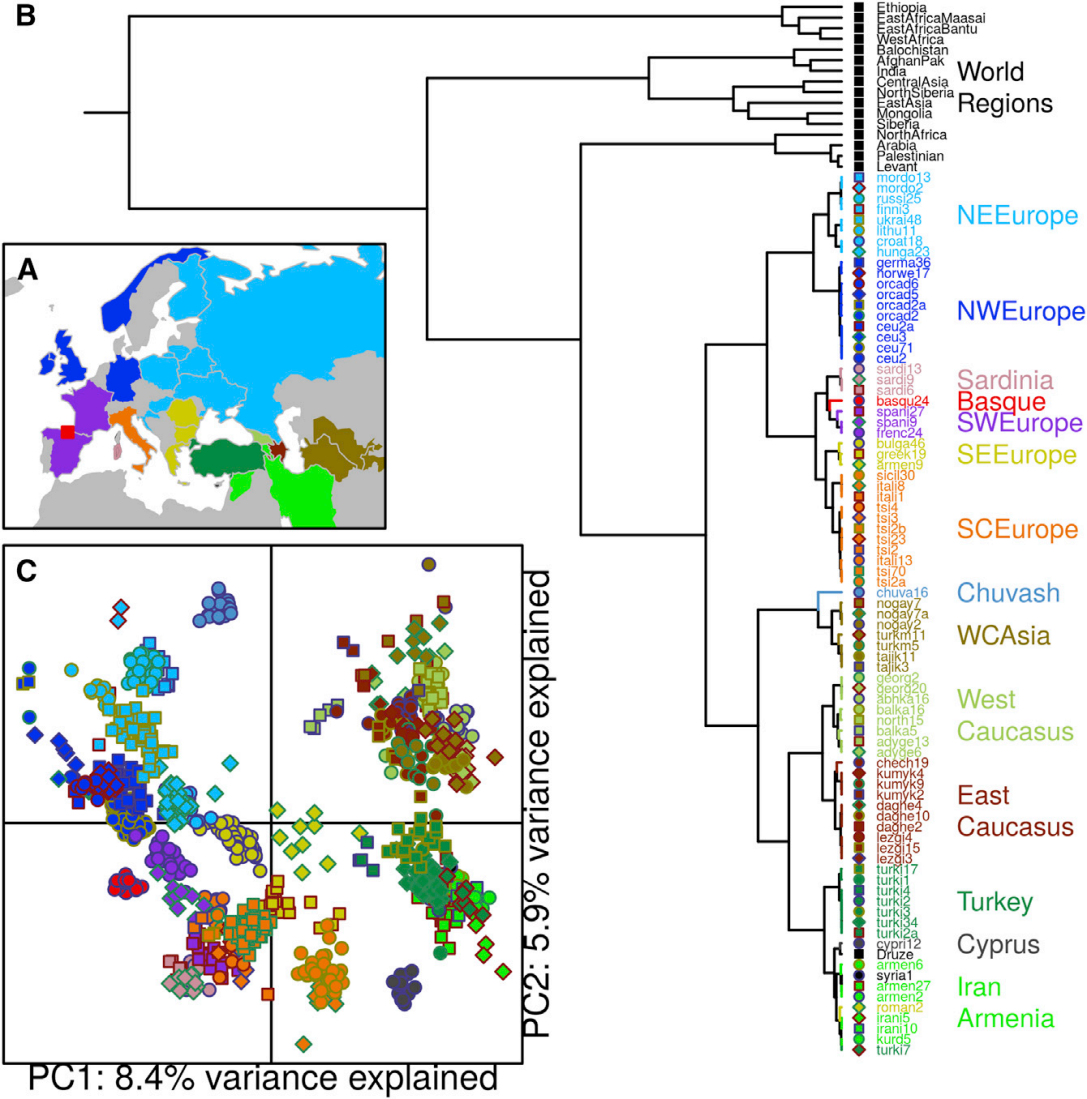
Structure and Relationships in West Eurasia (A) Map of West Eurasia showing the colors assigned to each major West Eurasian region. A full list of geographical populations used in the analysis is shown in Table S1. (B) The fineSTRUCTURE tree of the dataset showing the clusters used in the analysis. Cluster labels contain an alphabetical prefix relating to the geographical population label of the majority of individuals within a cluster. The numerical suffix describes the total number of individuals within a cluster. A full description of the identity of the individuals in each cluster is shown in Table S3. (C) Principal-component analysis (PCA) of the chunkcounts coancestry matrix used in fineSTRUCTURE. Each point is an individual and is labeled according to the fineSTRUCTURE cluster that it groups into as in (B).

Previous analyses of population structure have shown that despite high genetic similarity, European genetic structure is clinal and therefore heavily influenced by geography [12, 13]. But Eurasian populations are also genetically heterogeneous; in some countries (e.g., Sardinia) multiple genetic clusters of individuals can be inferred from genetic data, whereas in others (e.g., Basques), individuals are more similar [14]. To gain insight into the historical processes behind population genetic patterns in West Eurasia, we compiled a dataset of 1,235 phased West Eurasian genomes from 63 populations combined with 957 individuals from an additional 87 worldwide populations (Table S1) [11, 15, 16, 17]. We accounted for potential substructure within groups with the same geographic population label by performing an analysis of population structure using the fineSTRUCTURE [14] genetic clustering algorithm, which identifies groups of individuals who are statistically indistinguishable from each other from a genetic point of view (Figure 1 and Supplemental Information). This approach gains power over traditional methods of defining population structure like ADMIXTURE [18] or principal-component analysis (PCA) [19] by explicitly modeling the correlation structure among nearby SNPs due to linkage disequilibrium (LD) [20]. Moreover, because this clustering brings together individuals on the basis of shared ancestry, there should be a reduction in the noise in the admixture inference process that can come from having individuals with different ancestries within the same geographic population [21]. The algorithm additionally reconstructs the hierarchical relationships between the clusters, in the form of a tree, that allowed us to redefine West Eurasia as the monophyletic clade of 82 fineSTRUCTURE clusters containing 1,000 individuals (Figures 1 and S1B), which incorporates all of mainland Europe, Sardinia, Sicily, Cyprus, western Russia, the Caucasus, Turkey, and Iran, and some individuals from Tajikistan and Turkmenistan. Similarly, we defined 18 World Regions containing sets of related clusters within different broad geographic regions of the world. In general, within West Eurasia, we see that often the majority of individuals with the same geographic population label fall into the same genetic cluster (for example, the Basques, Greeks, and Mordovians), although sometimes individuals from larger geographic populations, such as Spain and HAPMAP CEU individuals (Northern and Western Europeans), are split into several clusters, which likely represents true substructure. In other cases, such substructure is not evident, and individuals from multiple populations are merged (for example Poland, Ukraine, Belorussia), suggesting that geographic population labels do not always describe genetic similarity, further motivating our genetic clustering approach (see also Figure S1A).

We investigated the effect of admixture—the process of mixing of haplotypes between genetically differentiated ancestral groups—in each of these clusters using GLOBETROTTER [11, 20]. First, we painted each recipient individual’s chromosomes such that they were represented as mosaics of chunks of different ancestry from a set of donor groups that included all 18 World Regions together with other clusters from within West Eurasia. We then used summaries of the amount of genome-wide donor ancestry from these mosaics, together with information on the lengths and distributions of specific ancestral chunks, to infer whether admixture is likely to have occurred in a recipient group and to characterize the composition and proportion (α) that each donor group contributed to the sources of the admixture event (Supplemental Information). We can infer the date of admixture by modeling the decay of LD between ancestral chunks, which decreases more rapidly the longer ago admixture occurred [11, 22]. GLOBETROTTER reconstructs admixture sources as mixtures of the available donor groups, which allows one to infer the properties of admixture when the donor groups are themselves admixed, making it particularly suited to the current setting. We attempted to infer admixture in all 82 West Eurasian clusters, but, with the exception of a Finnish cluster (finni3) that contained both of the Finnish individuals in the analysis (together with a Norwegian), to allow the algorithm to concentrate on identifying admixture from genetically well-defined donor groups, we removed all clusters with fewer than five individuals from being admixture donors, all of which were sub-groups of larger populations (Table S3).

### Admixture Is Common in West Eurasia

The vast majority of clusters (78%; 64 out of 82) showed evidence of admixture, suggesting that admixture-facilitated gene flow is a fundamental property of almost all West Eurasian groups (Tables S4 and S5; Supplemental Information). Here, we discuss the broader patterns of ancestry across West Eurasia, with a more detailed assessment of admixture events provided in the Supplemental Information. Throughout, we refer to the inferred groups characterized by GLOBETROTTER as contributing to an admixture event as “sources” and the sampled groups contributing ancestry to these sources as “donors.” It is also important to note that in the discussion presented below, we use current-day geographic labels to describe ancestry of historical sources of admixture. When we describe the ancestry of a particular source as, for example, “Mongolian,” this is a convenient but less precise proxy for “ancestry in a historical group that is related to the ancestry that we observe in contemporary Mongolian populations today.” This shorthand aids reading, but one must bear in mind that while the inferred sources of admixture are likely to be closely related genetically to the true historical admixing groups, because of subsequent population movements and migration, they may be less closely related geographically to the original source of that ancestry.

**Figure 2:**
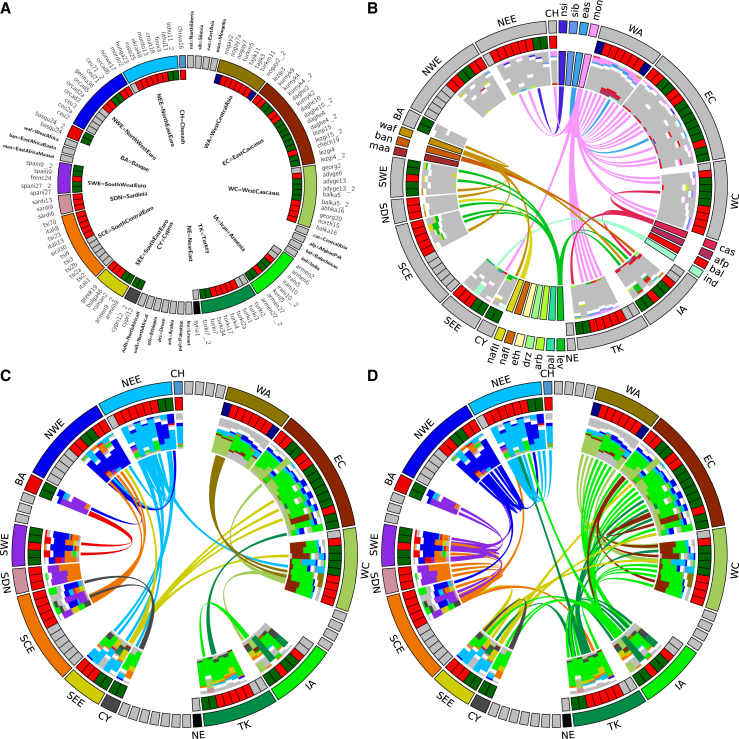
Summary of Eurasian Admixture Events Inferred by GLOBETROTTER (A) Key showing the position of each cluster in the circos plot. The inner circle describes the type of event inferred in each cluster: gray = no admixture; red = one date; green = one date multiway, blue = two dates. For the latter two types of events, two sets of sources are shown. Second event sources are suffixed with a 2. Clusters are ordered clockwise by increasing date within regions around the circle. Labels for plots B–D are shown in bold in (A) for West Eurasian source regions inside the circle and World Regions sources around the edge. (B) All events involving minor World Region sources. For each event, the two sources are shown as barplots; each source is split by whitespace, and the size of the two sources reflects the proportion that that source contributes to the admixture event. Each source is made up from a number of components whose colors reflect the World Region that the source component comes from. All Eurasian source components are grayed out. Although made up of components, each source can also be represented by a “best-matching” source, and the central links join the best-matching source (thick end of the link) to the recipient cluster (thin end). (C and D) Equivalent plots to (B) showing West Eurasian admixture components in color and World Region components in gray. Links in (C) join the best-matching minor West Eurasian sources to the clusters. Links in (D) join the best-matching major admixture source, which is always from West Eurasia, to the relevant cluster. Colors in (C) and (D) represent different regions to those in (B).

To visualize ancestry across West Eurasia, we constructed circos plots [23] where each segment of the circle represents a recipient group. These summaries describe the recent ancestry of the clusters: each admixture source is colored by contributions from different donor groups. We can then compare these mixed sources to the set of admixture donors to find the best-matching present-day donor group that is connected to events by links across the middle of the circles (Figures 2 and S3; Table S4). For any given event, based on the compositions of the sources, we identify the best-matching major admixture source, which is always most similar to a West Eurasian donor group, and the best-matching minor admixture source, which can be most similar to either a West Eurasian or World Region donor, and therefore define events in this way. The barplots in Figure 2B show that almost all of West Eurasia has some ancestry from the World Regions. Such World Region ancestry can be seen in the composition of sources involved in events in northern European groups (NWE and NEE), yet only three of the clusters containing individuals from this region derive ancestry from a source best matched by a World Region donor. Deconstruction of the admixture events in these northern European clusters shows that most mixing involves groups already present within West Eurasia (Figures 2C and S3). Assuming a generation time of 29 years [24], dates for these events center around the late first millennium CE, a time known to have involved significant upheaval in Europe (Figure 2B) [25].

### Recent Gene Flow into West Eurasia from Surrounding World Regions

**Figure 3:**
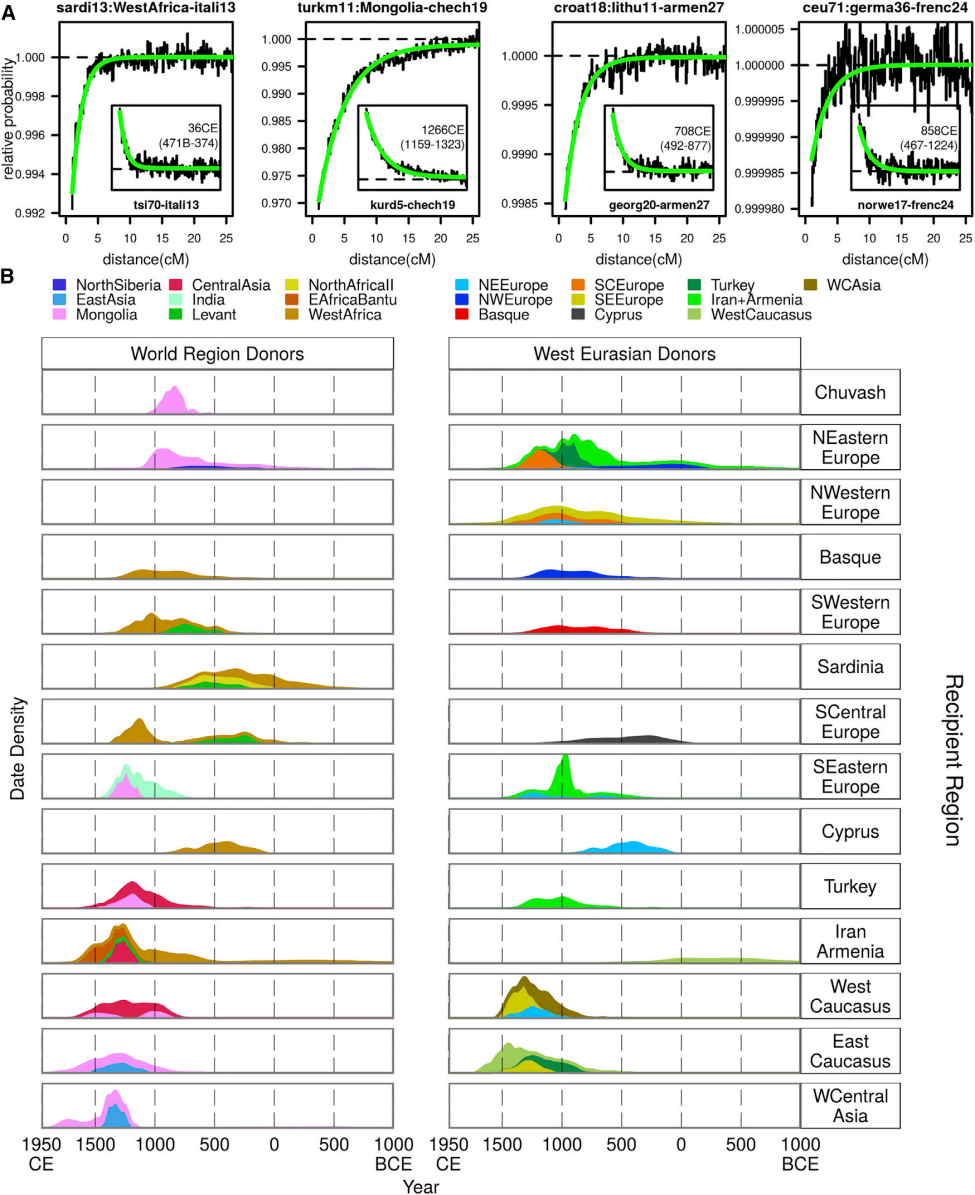
Dates of Eurasian Admixture Events Inferred by GLOBETROTTER (A) Example co-ancestry curves that we use to infer the date of admixture and composition of sources. For a given cluster, CHROMOPAINTER identifies the chunks of DNA within each individual’s genome that are most closely related ancestrally to each donor group. GLOBETROTTER measures the decay of association versus genetic distance between the chunks copied from a given pair of donor groups. Assuming a single pulse of admixture between two or more distinct admixing source groups, theoretical considerations predict that this decay will be exponentially distributed with rate equal to the time (in generations) that this admixture occurred [22]. GLOBETROTTER jointly fits an exponential distribution to the decay curves for all pairwise combinations of donor groups and determines the single best fitting rate, hence determining the most likely single admixture event and estimating the date it occurred. GLOBETROTTER aims to infer the haplotype composition of each source group for the admixture as a linear combination of those carried by sampled groups. This results in the admixed groups themselves automatically being represented in the same form—as a mixture of mixtures. The left-most plot of the four large plots shows the relative probability of jointly copying two chunks from West Africa and North Italian (itali13) donors, at varying genetic distances, in a Sardinian cluster (sardi13). The curves closely fit an exponential decay (green line) with a rate of 65 generations, or 36 CE. The negative slope for this WestAfrica-itali13 curve suggests that these donors contribute to different sides of an admixture event. The inset tsi70-itali13 curve has a positive slope, showing that tsi70 and itali13 contribute to the same side of the admixture event. We show similar pairs of curves for three other groups (turkm11, croat18, and ceu71) with varying dates and donors of admixture. (B) We define each admixture event by the West Eurasian region that the recipient group comes from (rows) and the identity of the best-matching current-day group to the minor admixture source (colors). We show dates separately for events involving World Region minor sources (left, events shown by links in Figure 2B) and West Eurasian minor sources (right, events shown by links in Figure 2C). For each region, all date bootstraps for events involving a best-matching source from the specified donor region are combined to generate a density. The integrals of the densities are proportional to the number of admixture events used to generate them.

**Figure 4:**
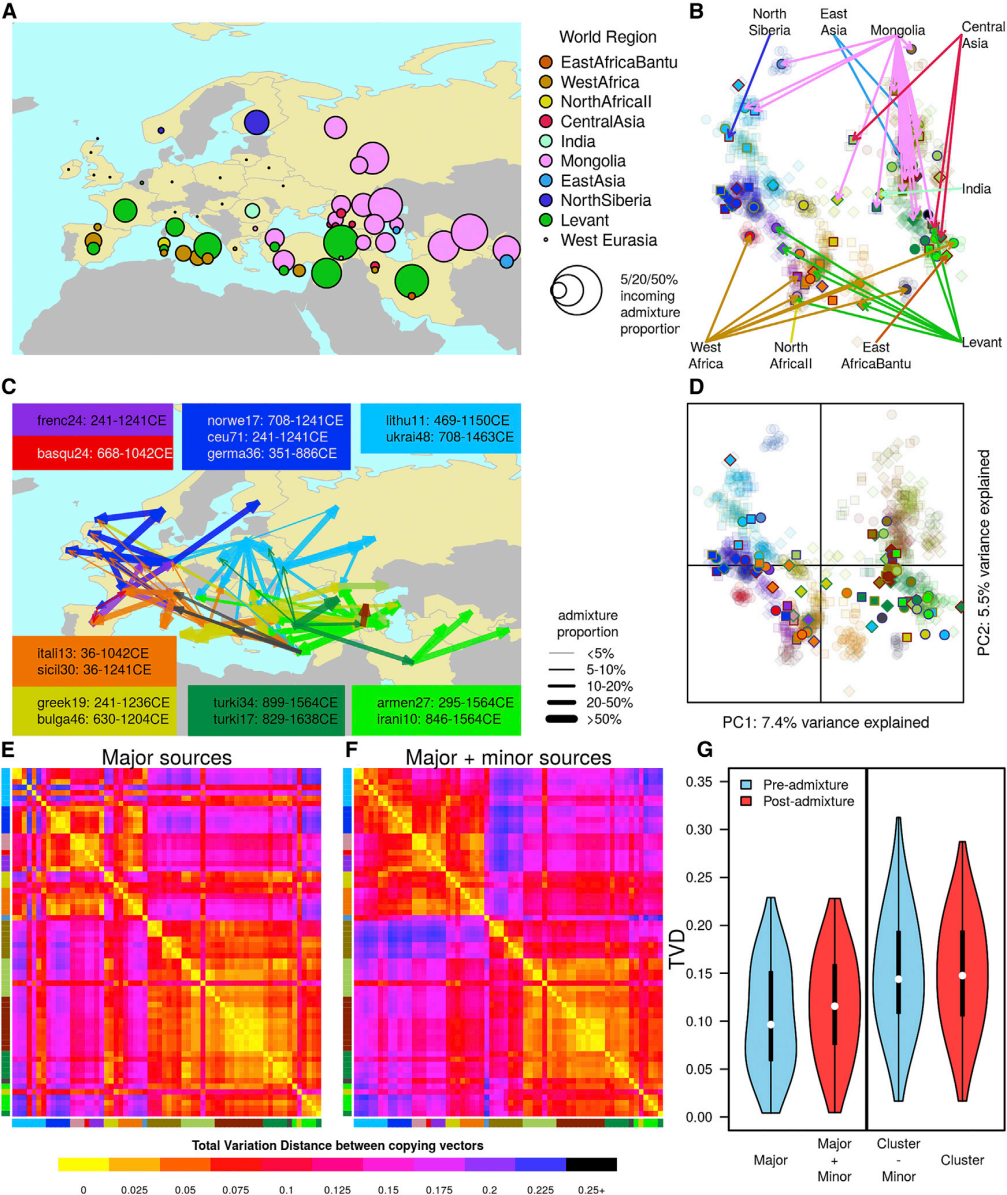
The Impact of Recent Admixture in West Eurasia (A) For each geographic sampling location, we estimated the proportion of ancestry coming from outside of West Eurasia by averaging GLOBETROTTER’s admixture inference across individuals from a sampling location. The sampling locations of each point are shown in Figure S4A; Caucasus populations are spread out to aid visibility. Points are stacked vertically in cases where multiple ancestries are present in a population. (B) Copying vectors of 82 West Eurasian fineSTRUCTURE clusters projected onto PCA based on the copying vectors of 1,000 West Eurasian individuals (faded colors; symbols and colors are as in Figure 1B); lines link World Region admixture sources to the clusters in which admixture from them is inferred. (C) Gene flow within West Eurasia is shown by lines linking the best-matching donor group to the sources of admixture with recipient clusters (arrowhead). Line colors represent the regional identity of the donor group, and line thickness represents the proportion of DNA coming from the donor group. Ranges of the dates (point estimates) for events involving sources most similar to selected donor groups are shown. (D and E) The pre-admixture structure of West Eurasian groups is shown by projecting all admixture source copying vectors that most closely match a West Eurasian group (n = 81) and the cluster copying vectors where we do not infer admixture (n = 18) onto the same PCA as (B). Heatmaps show pairwise total variation distance (TVD) between the Major admixture source copying vectors of all clusters where we infer admixture (n = 64; E) and copying vectors generated by combining the Major and Minor admixture sources at inferred admixture proportions. (F) In cases where we infer one date multiway or two dates, we show the major source for the first and/or most recent event only. Clusters are in the same order from top to bottom as in the tree in Figure 1, and axis colors describe the geographical origin of the cluster. (G) Violin plots comparing the distribution of TVD between the same two sets of copying vectors. The white point indicates the median value; the box shows the 25–75 percentiles; and the plots are truncated at the 2.5 and 97.5 percentiles. The colored shapes show kernel densities.

In contrast to relatively low levels in Northern Europe, ancestry from East Asia is much more visible in the West Central Asian, Caucasus, and Turkish clusters, where the influence of Mongolia (mon) in particular can be seen through the pink links and bars in Figure 2B and in Figure 4A. In West Central Asia (WA), some Central (cas) and East Asian (eas) ancestry is also present across this region. Within Anatolia (here defined as Armenia and Iran, IA, and Turkey, TK), West Central Asia (WA; including Nogai, Tajik, and Turkmen individuals), and several other groups from the Caucasus (EC and WC), events largely involve Asian sources, with the period after 1000 CE appearing to be important in the generation of the ancestry of this region (Figure 3B). Interestingly, the three events that do involve a Mongolian-like source in Northern Europe, in the Chuvash (CH; chuva16: 829 CE [627–940 CE]), Russians (russi25: 913 CE [754–1007 CE]), and Mordovians (mordo13: 792 CE [564–975 CE]) all date prior to 1000 CE, suggesting an origin from a different historical event to the more eastern groups (Figure 3B). Of the other Asian world regions, we only see direct admixture from North Siberia (nsib) into a Finnish cluster (finni3: 469 CE [213 BCE–1011 CE]; Figure 3B and Table S4) and from India (ind) into a cluster of two Romanians (roman2: 990 CE [741–1245 CE]), putatively of Romany origin. Nevertheless, observable ancestral components from Afghanistan and Pakistan groups (afp and bal) in WA, EC, WC, and IA suggests that ancestry from across Asia is shared with the more easterly West Eurasian groups.

Southern European groups (SEE, SCE, SDN, SWE, and BA) on the other hand derive ancestry from African and Near Eastern World Regions. In particular, ancestry from groups most similar to contemporary populations from in and around the Levant (lev; which we define as the World Region containing individuals from Syria, Palestine, Lebanon, Jordan, Saudi, Yemen, and Egypt) is present across Italy (SCE), Sardinia (SDN), France and Spain (SWE), and Armenia (IA; Figure 2B). Interestingly, North (nafII) and West (waf) African ancestry is also seen entering Southern Europe, suggesting a key role for the Mediterranean in supporting gene flow back into Europe [8, 26, 27]. Dates for the influx of this admixture are broad and generally fall within the first millennium CE (Figure 3B) although are more recent in BA and SWE, including French (frenc24: 728 CE [424–1011 CE]) and Spanish (spani27: 1042 CE [740–1201 CE]; spani9: 668 CE [286–876 CE]) clusters, consistent with migrations associated with the Arabic Conquest of the Iberian peninsula [8, 11, 28] and earlier movements in and around Italy [29].

### Movement within Europe during the Medieval Migration Period

When we consider the composition of sources from within West Eurasia (minor sources in Figure 2C and major sources in Figure 2D), while the majority of a group’s ancestry tends to come from its own regional area, there is a substantial contribution of both Northern European (light and dark blue) and Armenian groups (light green) to most WA, EC, WC, and TK clusters, as well as some clusters from both SEE and SCE. As previously reported [11], the formation of the Slavic people at around 1000 CE had a significant impact on the populations of Northern and Eastern Europe, a result that is supported by an analysis of identity by descent segments in European populations [10]. Here, despite characterizing populations by genetic similarity rather than geographic labels, we infer the same events involving a “Slavic” source (represented here by a cluster of Lithuanians; lithu11 and colored light blue) across all Balkan groups in the analysis (Greece, Bulgaria, Romania, Croatia, and Hungary) as well as in a large cluster of Germanic origin (germa36) and a composite cluster of eastern European individuals (ukrai48; Figures 4A and 4B). Dates for these events mostly overlap, although are older in Croatia and Greece, and appear to concentrate at the end of the first millennium CE (Figure 2B), a time known as the European Migration Period, or Völkerwanderung [25]. We additionally infer events during the period 300–1200 CE across Northern and Western Europe involving minor West Eurasian source groups from Europe (Figures 2D, 3B, and 4C). The date and composition of these events suggest a substantial amount of movement during the Völkerwanderung [25], providing persuasive evidence that this period had a visible effect on contemporary populations across Northern, Western, and Central Europe (Figure 4C).

### The Effect of Recent Admixture on Genomic Variation in West Eurasia

All peripheral populations analyzed have experienced recent admixture from World Regions (Figure 4A), and we also inferred recent mixing between many of the groups within West Eurasia (Figure 4C). We performed a variety of analyses using total variation distance (TVD) to understand and quantify the effect of these events on genetic variation (Figure 4 and Supplemental Information). Using the output of GLOBETROTTER, we considered “pre-admixture” variation in two ways: by using the inferred major source copying vectors directly and by removing the minor admixing source from the original cluster copying vector. Likewise, “post-admixture” variation can be inferred either by combining the inferred major and minor admixture sources in the appropriate admixture proportions or by using the cluster copying vectors directly. While the two sets of pre- and post-admixture copying vectors should be similar, in practice, they are unlikely to be identical, both because the admixture inference is unlikely to be perfect and because GLOBETROTTER is unable to fully account for genetic drift that may have occurred after admixture [11]. Comparisons of TVD between pairs of copying vectors inferred in these two ways show that when we re-generate a cluster from the admixture inference (Figure S4J), we systematically underestimate variation compared to the variation we observe when we use the contemporary clusters. In fact, for a given group, the differences between the two pre-admixture copying vectors and the two post-admixture copying vectors are highly correlated (Figure S4K), suggesting that the variation is mainly down to differences between the (observed) cluster copying vectors and GLOBETROTTER’s inferred sources of admixture. If this error is in part due to drift, then this suggests that drift after admixture may have acted to increase genetic differentiation.

When we compare the relative differences between pre- and post-admixture groups, we observe no appreciable difference between them, suggesting that admixture has not had a significant impact on genetic variation in West Eurasia (Figure 4G). Median TVD does, however, marginally decrease in the pre-admixture variation estimates (Figure 4G), which appears to be driven by differences between western (top left quadrant of Figures 4E and 4F) and eastern (lower right quadrant) West Eurasian groups. When we plot all admixture sources on a PCA based on contemporary individuals (Figure 4D), they tend to occur closer to the center of the plot, resembling the West Eurasian population structure inferred in a recent study of Bronze Age individuals [6]. Additional recent research using ancient DNA from multiple populations and time points in West Eurasia has demonstrated that there has been large-scale genetic turnover in Europe over the last 5,000 years [4, 5, 6, 30]. Our analysis supports this work by providing evidence that recent population movements have acted on top of this Bronze Age structure but also highlights a potential role for admixture and/or genetic drift in contributing to the genetic variation present in West Eurasia today.

Our results show that it is possible to draw complex inferences about recent human evolutionary past through the genomes of people alive today that are complementary to those made from ancient DNA. We caution that we are unlikely to have included individuals from all potential genetic donor groups to the current West Eurasian gene pool, and therefore, the gene flow events that we present should be viewed in the context of the dataset that we have used. Future work providing a better understanding of the phenotypic effects of World Region ancestry on contemporary populations as well as placing this work within the context of ancient DNA samples will further aid our understanding of Eurasian prehistory and disease. Nonetheless, the current analysis demonstrates that admixture has left a record in the genomes of all contemporary West Eurasians.

## Experimental Procedures

### Dataset

Our dataset included 40 newly genotyped individuals (20 each from Croatia and Daghestan) together with published data, choosing samples on the basis of shared genotyping platform (Illumina 550, 610, 660W) and relevance to the peopling of Western Eurasia [11, 15, 16, 17, 31] (Figure 1; Table S1). All datasets and genetic maps were based on build 36 of the human genome. We merged the datasets using PLINK (v.1.07) [32], and individuals and SNPs with call rates of less than 98% were dropped. Further quality control to remove cryptically related individuals based on identity by descent (IBD) and PCA was also performed. The final dataset contained 2,192 individuals from 144 populations typed on 477,812 SNPs (Table S1), which were computationally phased together using SHAPEITv1 [33]. Individuals who provided samples gave informed consent following ethical approval by the ethics committees at the various universities where the samples were collected.

### Defining Analysis Clusters

We ran fineSTRUCTURE [14] to cluster individuals and identified 18 World Regions based on this clustering (Figure 1; Table S2). Fixing these groups, we re-ran the algorithm twice, identifying the final list of 82 Eurasian clusters (Table S3) based on comparisons between these two runs. Clusters are therefore based on genetic similarity only (see also Figure S1). PCAs in Figures 1 and S4 were generated by performing a PCA on the CHROMOPAINTER chunkcounts matrix using the *prcomp* function in R [34]. Further details are described in the Supplemental Information.

### Inferring Complex Admixture with GLOBETROTTER

We describe the detailed process of inferring admixture with GLOBETROTTER in the Supplemental Information. Briefly, we first used CHROMOPAINTER, a chromosome-painting method that reconstructs each individual genome as a mosaic of all donor groups, to identify the subset of donors that share material with the recipient group. Next, because closely related individuals share long stretches of DNA, with the length of these chunks shortening as individuals become less related, we used the paintings to infer the distribution of ancestral chunks at different genetic distances along the genome, and build “coancestry curves” for each pair of putative donor populations (Figure 3A). Assuming a single pulse of admixture involving two genetically distinct sources, the exponential decay of these curves is proportional to the time since genetic material from the two donor groups came together and thus provides a date of the admixture event [11, 22]. Finally, we sequentially removed donor groups from the analysis where such curves were no different from background noise, a step that allowed us to (re-)assess the makeup of the contributing source groups and to identify whether two groups occur on the same side of an admixture event. We performed further tests on these curves, allowing us to assess whether admixture has occurred at multiple times in a group (i.e. we tried to fit multiple exponentials to the coancestry curves) and whether admixture occurred with more than two admixing source groups. We tested the robustness of the admixture inference by comparing these curves with those generated by considering CHROMOPAINTER painting samples from different individuals, leveraging the idea that ancestry LD characteristically decays within individual genomes much more strongly than when ancestry is measured in different individuals (Supplemental Information).

### Characterizing Admixture Events and Source Copying Vectors

In cases where we inferred admixture (p < 0.01), we then characterized the admixture as one date (1D), one date multiway (1MW), or multiple dates (2D). For each event in each cluster, we inferred the proportion, α and date(s), λ, of admixture together with a set of βs, which describe the composition of the admixing sources. 1D events have two admixing sources; 1MW and 2D events have four admixing sources. To infer copying vectors for the admixture sources, we took the β coefficients for a given source and multiplied each by their respective copying vectors (see the Supplemental Information for a detailed discussion of this approach). In Figure 4, to assay pre-admixture variation, we showed comparisons between major source copying vectors (Major) and clusters with admixture sources removed (Cluster − Minor), and for post-admixture variation, we use the inferred admixed group (Major + Minor) and contemporary cluster copying vectors.

We generated 100 date bootstraps by re-estimating the date of admixture sampling the painted samples from all individuals in a cluster with replacement. In the text, figures, and tables, we converted time in admixture in generations to historical time assuming a generation time of 29 years [24]. In Figure 3, date bootstraps are combined across all events involving best-matching sources from a given region and then grouped by the region that the target cluster comes from.

### Comparing Sets of Copying Vectors

We used TVD to compare copying vectors [20]. As the copying vectors are discrete probability distributions over the same set of donors, TVD is a natural metric for quantifying the difference between them. For a given pair of groups *A* and *B* with copying vectors describing the copying from *i* donors, *a*_i_ and *b*_*i*_, we can estimate TVD with the following equation:

$$\mathrm{TVD}=0.5\times \sum_{i=1}^{n}\left(\left|a_{i}-b_{i}\right|\right)$$

To compare variation in West Eurasia before and after admixture, we estimated TVD for each pair of copying vectors and show the distribution as violin plots (Figure 4G) and boxplots (Figure S4B).

## Author Contributions

C.C. and G.B.J.B. conceived and designed the research. G.H., S.M., and G.B.J.B. developed methods. G.B.J.B. and F.M. performed analyses. S.T., K.B., I.R., T.Z., C.H., D.T., S.K.-Y., D.N., P.A., F.C., F.B., V.R., G.L., C.B., J.B.C., A.H.-K., S.D., R.P., P.K., T.H., T.M., R.J.H., and J.F.W. provided DNA samples for genotyping. G.B.J.B. and C.C. wrote the paper, which was reviewed by all authors.

## Accession Numbers

The accession number for the Croatian samples genotyped for this study is GEO: GSE71603.

## Supplemental Information

Document S1. Supplemental Experimental Procedures, Figures S1–S4, and Tables S1–S4

Table S5. Results of All GLOBETROTTER Analyses Performed on 82 Eurasian Clusters, Related to Figure 4 Column headings are as in Table S4. All clusters were analyzed once using all clusters with at least five individuals as surrogates (main analysis). Others were also analyzed with bespoke masked analyses, as noted in the main text. The NULL procedure was run for each of these analyses for comparative purposes.

Document S2. Article plus Supplemental Information
