# The Role of Recent Admixture in Forming the Contemporary West Eurasian Genomic Landscape

**DOI:** 10.1016/j.cub.2015.10.037

**Published:** 2015-11-02

**Authors:** George B.J. Busby, Garrett Hellenthal, Francesco Montinaro, Sergio Tofanelli, Kazima Bulayeva, Igor Rudan, Tatijana Zemunik, Caroline Hayward, Draga Toncheva, Sena Karachanak-Yankova, Desislava Nesheva, Paolo Anagnostou, Francesco Cali, Francesca Brisighelli, Valentino Romano, Gerard Lefranc, Catherine Buresi, Jemni Ben Chibani, Amel Haj-Khelil, Sabri Denden, Rafal Ploski, Pawel Krajewski, Tor Hervig, Torolf Moen, Rene J. Herrera, James F. Wilson, Simon Myers, Cristian Capelli

(Current Biology *25*, 2518–2526; October 5, 2015)

Due to an author oversight during the production process, the Supplemental Information PDF originally published with this article online omitted the Supplemental References list. Also, the reference citations throughout the Supplemental Information PDF were incorrectly numbered as “[S?]” instead of “[S1],” “[S2],” etc. The PDF has now been updated online to include the Supplemental References list, and to correct the reference citations throughout. The authors apologize for the inconvenience.

