# Supplementary material for: The Role of Recent Admixture in Forming the Contemporary West Eurasian Genomic Landscape
Source: Curr Biol. 2015 Oct 5;25(19):2518–26. doi: 10.1016/j.cub.2015.08.007 (PMC4714572; doi:10.1016/j.cub.2015.08.007)
Supplement: Document S1. Supplemental Experimental Procedures, Figures S1–S4, and Tables S1–S4 [file mmc1.pdf]

Current Biology

Supplemental Information

# The Role of Recent Admixture in Forming the Contemporary West Eurasian Genomic Landscape

George B.J. Busby, Garrett Hellenthal, Francesco Montinaro, Sergio Tofanelli, Kazima Bulayeva, Igor Rudan, Tatijana Zemunik, Caroline Hayward, Draga Toncheva, Sena Karachanak-Yankova, Desislava Nesheva, Paolo Anagnostou, Francesco Cali, Francesca Brisighelli, Valentino Romano, Gerard Lefranc, Catherine Buresi, Jemni Ben Chibani, Amel Haj-Khelil, Sabri Denden, Rafal Ploski, Pawel Krajewski, Tor Hervig, Torolf Moen, Rene J. Herrera, James F. Wilson, Simon Myers, and Cristian Capelli

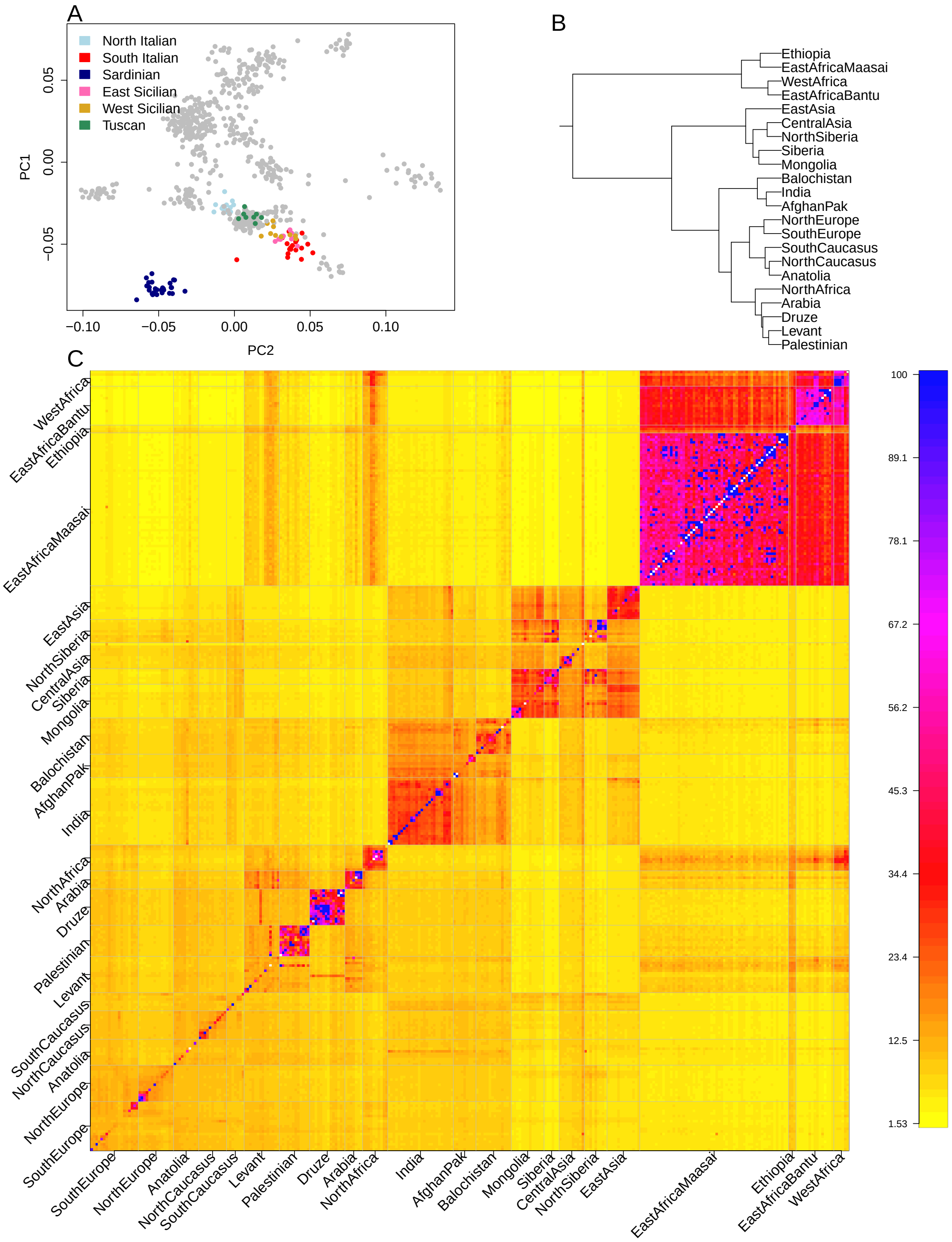


**Figure S1.** Identification of genetic populations and world regions, related to Figure 1. (A) PCA plot of European SNP genotypes with some Italian populations highlighted shows differentiation between Italian sub-populations (B) The collapsed fineSTRUCTURE tree generated by successively merging groups to generate world regions. (C) The CHROMOPAINTER chunkcount coancestry matrix ordered by the result from the full fineSTRUCTURE analysis based on the worldwide analysis of 2192 individuals which we use to define our analysis clusters. Each row of the heatmap represents a copying vector, with the number of chunks copied from each donor individual as columns. Individuals are ordered by world region and the heatmap is capped at 100 chunks.

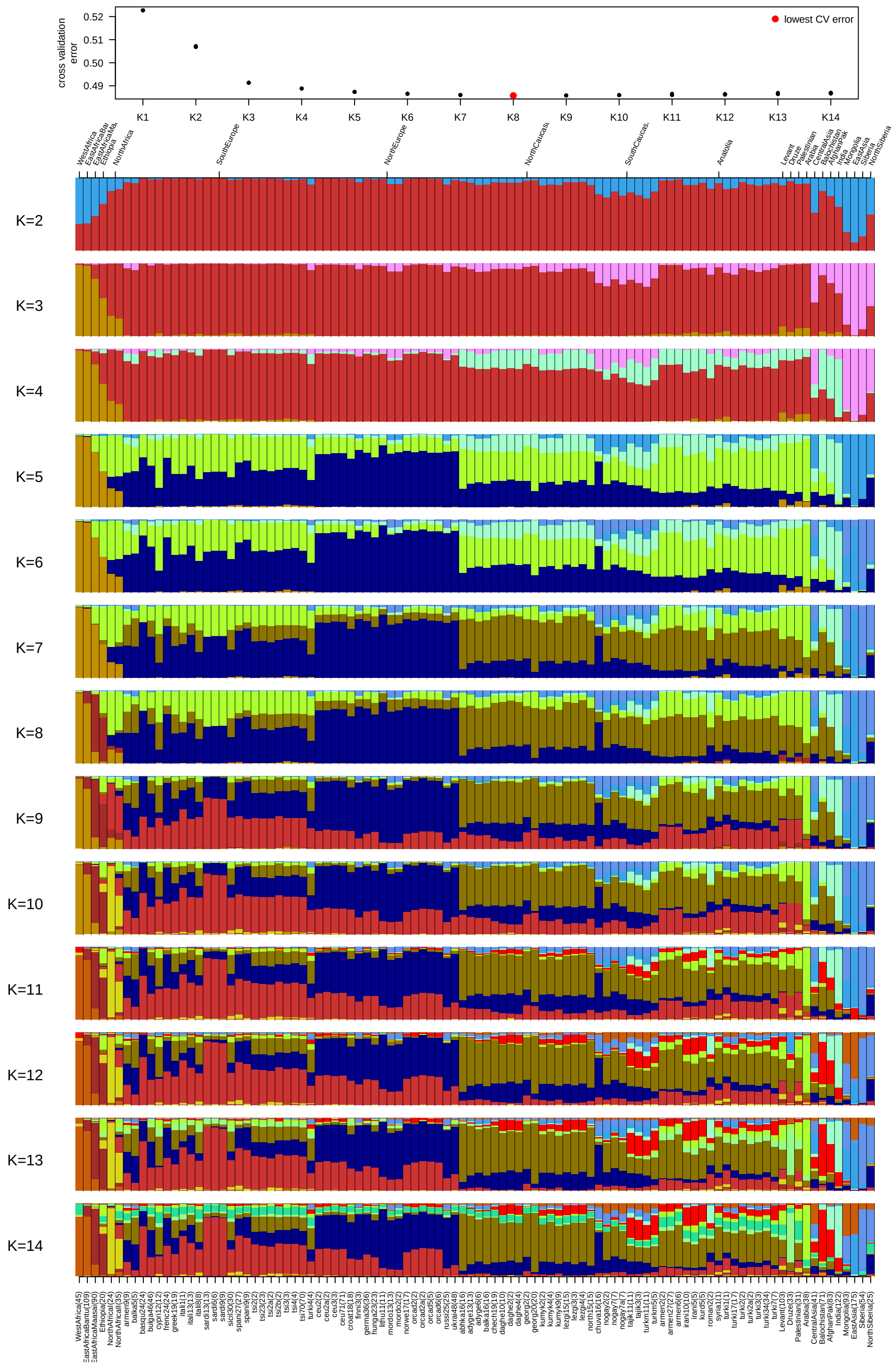


**Figure S2.** An ADMIXTURE analysis of the dataset showing the average admixture proportions for each cluster and world region. The top plot shows the cross validation error across multiple runs picking 8 as the optimum number of clusters. The number of individuals in each cluster world/region is in parentheses after the cluster/region name. We used an LD ($R^2$) threshold of 0.2.

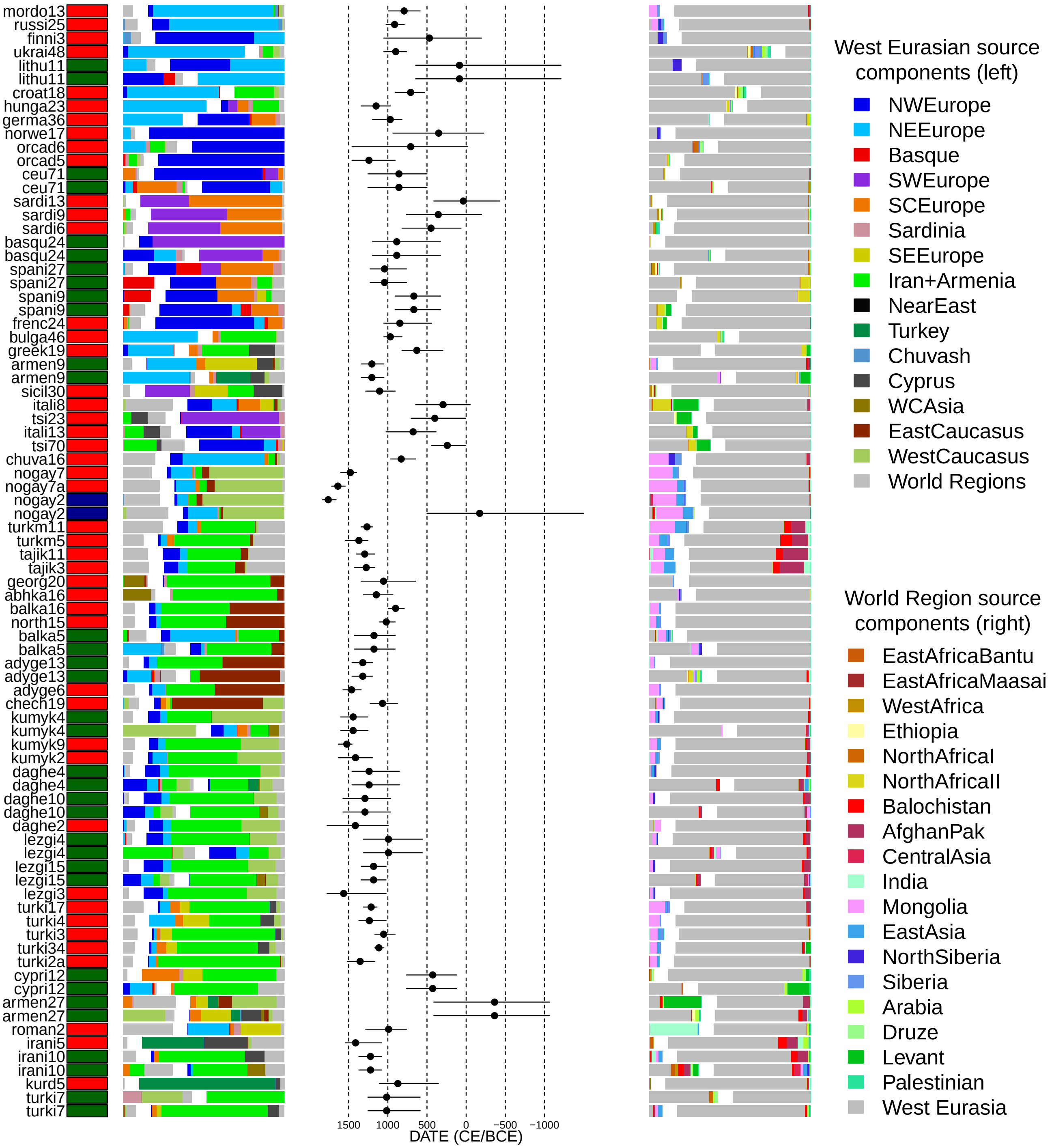


**Figure S3.** Proportions and dates of admixture shown in Figures 2 and 3. For each cluster we show the result of admixture inference, red = 1D (one date of admixture), darkgreen = 1MW (one date, multiple admixing groups), and darkblue = 2D (two admixture dates). Proportions of the two admixing sources of either side of an admixture event are shown as barplots with their components coloured by donor region. In the left-hand barplot, all non-West Eurasian components are greyed out, whereas the opposite is true for the right hand barplots. The colours of the bars represent the ancestry components detailed in the legends on the right. The date of admixture, with bootstrap CI is also shown in the central plot.

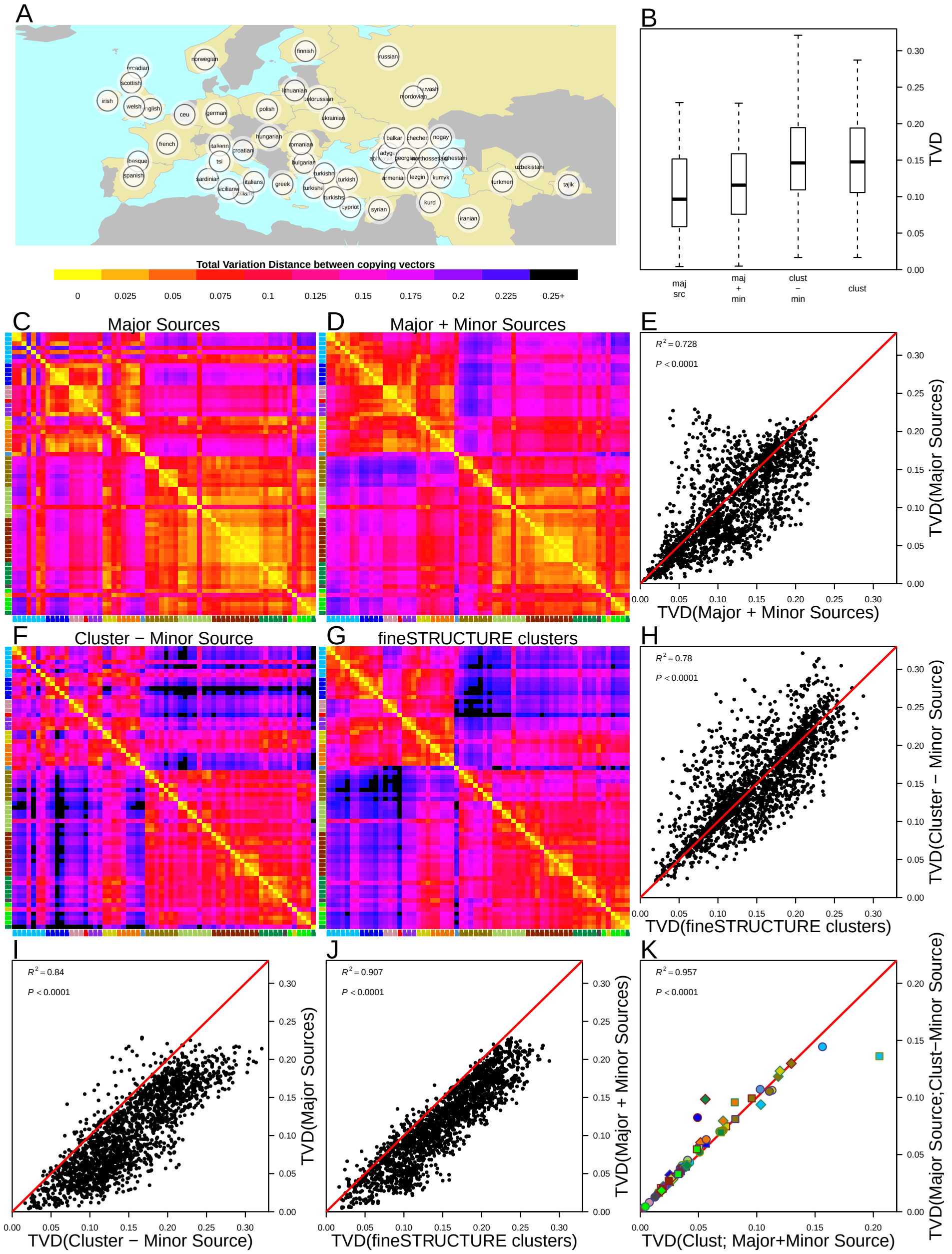


**Figure S4.** Additional plots related to Figure 4. (A) The geographic position of all populations used to generate Figure 4. For the 64 clusters where we infer admixture we show (B) boxplot showing the distibution of Total Variation Distance (TVD) for four sets of copying vectors: maj src = Major sources; maj + min = copying vectors generated by combining the major and minor source of admixture at inferred admixture proportions; clust - min = the fineSTUCTURE cluster copying vectors with the minor source of admixture removed; and clust = the fineSTRUCTURE copying vectors. (C,D,F,G) For the same four groups we show heatmaps of pairwise TVD. (E,H,I,J) pairwise comparisons of TVD computed separately for the four groups shows that variation tends to be higher when considering the fineSTRUCTURE clusters. (K) For each cluster where we infer admixture we show, the TVD between a copying vector generated from adding the major and minor sources together and the original fineSTRUCTURE cluster ("post-admixture"; x-axis) against the TVD of a copying vector generated by removing the minor source from the fineSTRUCTURE cluster copying vector and the major source of admixture ("pre-admixture"; y-axis). $R^2$ correlation coefficients and $P$-values (t-test, 62 degrees of freedom) are shown for all comparisons.

**Table S1.** Overview of sampled populations describing the continent, region, numbers of individuals used, and the source of any previously published datasets.

| Population | Continent | Region | n(pre-QC) | n(post-QC) | Source |
|---|---|---|---|---|---|
| bantusa | Africa | SubAfrica | 8 | 8 | Li, et al 2008 |
| luhya | Africa | SubAfrica | 110 | 94 | HAPMAP |
| maasai | Africa | SubAfrica | 156 | 97 | HAPMAP |
| mandenka | Africa | SubAfrica | 22 | 21 | Li, et al 2008 |
| yoruba | Africa | SubAfrica | 21 | 21 | Li, et al 2008 |
| ethiopiana | Africa | NorthAfrica | 7 | 7 | Behar, et al 2010 |
| ethiopiano | Africa | NorthAfrica | 7 | 7 | Behar, et al 2010 |
| ethiopiant | Africa | NorthAfrica | 5 | 5 | Behar, et al 2010 |
| egyptian | Africa | NorthAfrica | 12 | 12 | Behar, et al 2010 |
| moroccan | Africa | NorthAfrica | 25 | 25 | Hellenthal, et al 2014; Behar, et al 2010 |
| mozabite | Africa | NorthAfrica | 29 | 28 | Li, et al 2008 |
| tunisian | Africa | NorthAfrica | 16 | 9 | Hellenthal, et al 2014 |
| chechen | Eurasia | EastCaucasus | 20 | 20 | Yunusbayev, et al 2011 |
| daghestani/tabasaran | Eurasia | EastCaucasus | 20 | 20 | current study |
| kumyk | Eurasia | EastCaucasus | 14 | 14 | Yunusbayev, et al 2011 |
| lezgin | Eurasia | EastCaucasus | 18 | 18 | Behar, et al 2010 |
| abhkasian | Eurasia | WestCaucasus | 20 | 20 | Yunusbayev, et al 2011 |
| adygei | Eurasia | WestCaucasus | 17 | 17 | Yunusbayev, et al 2011 |
| balkar | Eurasia | WestCaucasus | 19 | 19 | Yunusbayev, et al 2011 |
| georgian | Eurasia | WestCaucasus | 20 | 20 | Yunusbayev, et al 2011 |
| northossetian | Eurasia | WestCaucasus | 15 | 15 | Yunusbayev, et al 2011 |
| armenian | Eurasia | Armenia/Iran | 35 | 35 | Yunusbayev, et al 2011 |
| iranian | Eurasia | Armenia/Iran | 20 | 19 | Behar, et al 2010 |
| kurd | Eurasia | Armenia/Iran | 6 | 6 | Yunusbayev, et al 2011 |
| turkishe | Eurasia | Turkey | 23 | 23 | Hodoğlugil et al 2012 |
| turkishn | Eurasia | Turkey | 20 | 20 | Hodoğlugil et al 2012 |
| turkishs | Eurasia | Turkey | 20 | 20 | Hodoğlugil et al 2012 |
| turkish | Eurasia | Turkey | 19 | 19 | Behar, et al 2010 |
| cypriot | Eurasia | Cyprus | 12 | 12 | Behar, et al 2010 |
| bedouin | Eurasia | NearEast | 46 | 39 | Li, et al 2008 |
| druze | Eurasia | NearEast | 42 | 41 | Li, et al 2008 |
| jordanian | Eurasia | NearEast | 20 | 19 | Li, et al 2008 |
| lebanese | Eurasia | NearEast | 8 | 5 | Behar, et al 2010 |
| palestinian | Eurasia | NearEast | 46 | 39 | Li, et al 2008 |
| saudi | Eurasia | NearEast | 20 | 19 | Behar, et al 2010 |
| syrian | Eurasia | NearEast | 16 | 15 | Behar, et al 2010 |
| uae | Eurasia | NearEast | 19 | 13 | Hellenthal, et al 2014 |
| yemeni | Eurasia | NearEast | 10 | 5 | Behar, et al 2010 |
| chuvash | Eurasia | Chuvash | 17 | 17 | Behar, et al 2010 |
| nogay | Eurasia | WestCentralAsia | 16 | 16 | Yunusbayev, et al 2011 |
| tajik | Eurasia | WestCentralAsia | 15 | 15 | Yunusbayev, et al 2011 |
| turkmen | Eurasia | WestCentralAsia | 15 | 10 | Yunusbayev, et al 2011 |
| hazara | Eurasia | CentralAsia | 22 | 20 | Li, et al 2008 |
| uygur | Eurasia | CentralAsia | 10 | 10 | Li, et al 2008 |
| uzbekistani | Eurasia | CentralAsia | 15 | 15 | Yunusbayev, et al 2011 |
| burusho | Asia | CentralAsia | 25 | 25 | Li, et al 2008 |
| kalash | Asia | CentralAsia | 23 | 1 | Li, et al 2008 |
| pathan | Asia | CentralAsia | 22 | 22 | Li, et al 2008 |
| sindhi | Asia | CentralAsia | 24 | 23 | Li, et al 2008 |
| balochi | Asia | CentralAsia | 24 | 23 | Li, et al 2008 |
| brahui | Asia | CentralAsia | 25 | 23 | Li, et al 2008 |
| makrani | Asia | CentralAsia | 25 | 20 | Li, et al 2008 |
| kyrgyz | Asia | CentralAsia | 16 | 16 | Hodoğlugil et al 2012 |
| cambodian | Asia | SouthAsia | 10 | 10 | Metspalu, et al 2011; Chaubey et al 2012 |
| brahmin | Asia | SouthAsia | 11 | 11 | Metspalu, et al 2011; Chaubey et al 2012 |
| gond | Asia | SouthAsia | 4 | 4 | Metspalu, et al 2011; Chaubey et al 2012 |
| kshatriya | Asia | SouthAsia | 7 | 7 | Metspalu, et al 2011; Chaubey et al 2012 |
| meena | Asia | SouthAsia | 1 | 1 | Metspalu, et al 2011; Chaubey et al 2012 |
| bengali | Asia | SouthAsia | 1 | 1 | Metspalu, et al 2011; Chaubey et al 2012 |
| bhunjia | Asia | SouthAsia | 1 | 1 | Metspalu, et al 2011; Chaubey et al 2012 |
| chamar | Asia | SouthAsia | 10 | 10 | Metspalu, et al 2011; Chaubey et al 2012 |

Continued on next page

Table S1 Continued from previous page

| Population | Continent | Region | n(pre-QC) | n(post-QC) | Source |
|---|---|---|---|---|---|
| chenchu | Asia | SouthAsia | 4 | 4 | Metspalu, et al 2011; Chaubey et al 2012 |
| dharkar | Asia | SouthAsia | 12 | 8 | Metspalu, et al 2011; Chaubey et al 2012 |
| dhurwa | Asia | SouthAsia | 1 | 1 | Metspalu, et al 2011; Chaubey et al 2012 |
| dusadh | Asia | SouthAsia | 10 | 7 | Metspalu, et al 2011; Chaubey et al 2012 |
| hakkipikki | Asia | SouthAsia | 4 | 3 | Metspalu, et al 2011; Chaubey et al 2012 |
| kanjar | Asia | SouthAsia | 8 | 5 | Metspalu, et al 2011; Chaubey et al 2012 |
| karnataka | Asia | SouthAsia | 9 | 8 | Behar, et al 2010 |
| kol | Asia | SouthAsia | 17 | 16 | Metspalu, et al 2011; Chaubey et al 2012 |
| kurmi | Asia | SouthAsia | 1 | 1 | Metspalu, et al 2011; Chaubey et al 2012 |
| kurumba | Asia | SouthAsia | 4 | 4 | Metspalu, et al 2011; Chaubey et al 2012 |
| lambadi | Asia | SouthAsia | 1 | 1 | Metspalu, et al 2011; Chaubey et al 2012 |
| malayan | Asia | SouthAsia | 1 | 1 | Behar, et al 2010 |
| mawasi | Asia | SouthAsia | 1 | 1 | Metspalu, et al 2011; Chaubey et al 2012 |
| meghawal | Asia | SouthAsia | 1 | 1 | Metspalu, et al 2011; Chaubey et al 2012 |
| muslim | Asia | SouthAsia | 5 | 5 | Metspalu, et al 2011; Chaubey et al 2012 |
| myanmar | Asia | SouthAsia | 3 | 3 | Behar, et al 2010 |
| nihali | Asia | SouthAsia | 2 | 2 | Metspalu, et al 2011; Chaubey et al 2012 |
| piramalaikallar | Asia | SouthAsia | 8 | 8 | Metspalu, et al 2011; Chaubey et al 2012 |
| sakd | Asia | SouthAsia | 4 | 4 | Behar, et al 2010 |
| tamilnadu | Asia | SouthAsia | 2 | 2 | Metspalu, et al 2011; Chaubey et al 2012 |
| tharus | Asia | SouthAsia | 2 | 2 | Metspalu, et al 2011; Chaubey et al 2012 |
| upcaste | Asia | SouthAsia | 5 | 5 | Metspalu, et al 2011; Chaubey et al 2012 |
| velamas | Asia | SouthAsia | 10 | 9 | Metspalu, et al 2011; Chaubey et al 2012 |
| han | Asia | EastAsia | 34 | 34 | Li, et al 2008 |
| hannchina | Asia | EastAsia | 10 | 10 | Li, et al 2008 |
| japanese | Asia | EastAsia | 28 | 28 | Li, et al 2008 |
| naga | Asia | EastAsia | 4 | 4 | Metspalu, et al 2011; Chaubey et al 2012 |
| naxi | Asia | EastAsia | 8 | 7 | Li, et al 2008 |
| tu | Asia | EastAsia | 10 | 10 | Li, et al 2008 |
| xibo | Asia | EastAsia | 9 | 9 | Li, et al 2008 |
| yi | Asia | EastAsia | 10 | 10 | Li, et al 2008 |
| dai | Asia | EastAsia | 10 | 10 | Li, et al 2008 |
| lahu | Asia | EastAsia | 8 | 6 | Li, et al 2008 |
| miao | Asia | EastAsia | 10 | 10 | Li, et al 2008 |
| she | Asia | EastAsia | 10 | 9 | Li, et al 2008 |
| tujia | Asia | EastAsia | 10 | 10 | Li, et al 2008 |
| buryat | Asia | EastAsia | 17 | 15 | Metspalu, et al 2011; Chaubey et al 2012 |
| daur | Asia | EastAsia | 9 | 9 | Li, et al 2008 |
| hezhen | Asia | EastAsia | 8 | 7 | Li, et al 2008 |
| mongolian | Asia | EastAsia | 19 | 19 | Li, et al 2008 |
| oroqen | Asia | EastAsia | 9 | 9 | Li, et al 2008 |
| yakut | Asia | EastAsia | 25 | 25 | Li, et al 2008 |
| altai | Asia | Siberia | 13 | 13 | Rasmussen, et al 2010 |
| burya | Asia | Siberia | 2 | 2 | Rasmussen, et al 2010 |
| tuva | Asia | Siberia | 16 | 13 | Rasmussen, et al 2010 |
| chukchi | Asia | Siberia | 15 | 5 | Rasmussen, et al 2010 |
| dolgan | Asia | Siberia | 7 | 7 | Rasmussen, et al 2010 |
| evenk | Asia | Siberia | 16 | 12 | Rasmussen, et al 2010 |
| ket | Asia | Siberia | 2 | 2 | Rasmussen, et al 2010 |
| koryake | Asia | Siberia | 18 | 5 | Rasmussen, et al 2010 |
| selkup | Asia | Siberia | 10 | 10 | Rasmussen, et al 2010 |
| yukagir | Asia | Siberia | 9 | 4 | Rasmussen, et al 2010 |
| nganassan | Asia | Siberia | 15 | 10 | Rasmussen, et al 2010 |
| basque | Europe | Basque | 24 | 24 | Li, et al 2008 |
| finnish | Europe | NEEurope | 2 | 2 | Hellenthal, et al 2014 |
| belorussian | Europe | NEEurope | 10 | 9 | Behar, et al 2010 |
| lithuanian | Europe | NEEurope | 10 | 10 | Behar, et al 2010 |
| mordovian | Europe | NEEurope | 15 | 15 | Behar, et al 2010 |
| polish | Europe | NEEurope | 18 | 17 | Hellenthal, et al 2014 |
| russian | Europe | NEEurope | 25 | 25 | Li, et al 2008 |
| ukrainian | Europe | NEEurope | 20 | 20 | Yunusbayev, et al 2011 |
| german | Europe | NWEurope | 30 | 30 | current study |
| ceu | Europe | NWEurope | 59 | 59 | HAPMAP |
| english | Europe | NWEurope | 8 | 8 | Hellenthal, et al 2014 |

Continued on next page

Table S1 Continued from previous page

| Population | Continent | Region | n(pre-QC) | n(post-QC) | Source |
|---|---|---|---|---|---|
| irish | Europe | NWEurope | 7 | 7 | Hellenthal, et al 2014 |
| norwegian | Europe | NWEurope | 18 | 18 | Hellenthal, et al 2014 |
| scottish | Europe | NWEurope | 8 | 6 | Hellenthal, et al 2014 |
| welsh | Europe | NWEurope | 4 | 4 | Hellenthal, et al 2014 |
| orcadian | Europe | NWEurope | 15 | 15 | Li, et al 2008 |
| sardinian | Europe | Sardinia | 28 | 28 | Li, et al 2008 |
| italiann | Europe | SCEurope | 12 | 12 | Li, et al 2008 |
| tsi | Europe | SCEurope | 102 | 98 | HAPMAP |
| tuscan | Europe | SCEurope | 8 | 8 | Li, et al 2008 |
| bulgarian | Europe | SEEurope | 31 | 31 | Hellenthal, et al 2014; Yunusbayev, et al 2011 |
| croatian | Europe | NEEurope | 20 | 19 | current study |
| hungarian | Europe | NEEurope | 20 | 19 | Behar, et al 2010 |
| romanian | Europe | SEEurope | 16 | 16 | Behar, et al 2010 |
| greek | Europe | SEEurope | 22 | 20 | Hellenthal, et al 2014 |
| italians | Europe | SCEurope | 18 | 18 | Hellenthal, et al 2014 |
| siciliane | Europe | SCEurope | 10 | 10 | Hellenthal, et al 2014 |
| sicilianw | Europe | SCEurope | 10 | 10 | Hellenthal, et al 2014 |
| french | Europe | SWEurope | 28 | 28 | Li, et al 2008 |
| spanish | Europe | SWEurope | 36 | 34 | Hellenthal, et al 2014; Behar, et al 2010 |
| 144 | 4 | 21 | 2422 | 2192 | |

**Table S2.** Composition of the World Regions identified by the fineSTRUCTURE analysis. For each World Region we report the geographic origin of all individuals within the group. The n(by population) column displays the population followed by the number of individuals from that population.

| WorldRegion | n(total) | n(by population) |
|---|---|---|
| Palestinian | 31 | palestinian31 |
| Levant | 103 | jordanian19 bedouin18 syrian13 egyptian11 druze8 palestinian8 saudi7 lebanese5 uae5 yemeni5 iranian4 |
| Druze | 33 | druze33 |
| Arabia | 38 | bedouin21 saudi12 uae5 |
| NorthAfricaI | 24 | mozabite24 |
| NorthAfricaII | 35 | moroccan24 tunisian9 mozabite2 |
| Armenia/Iran | 126 | armenian32 turkishe20 turkish18 iranian15 turkishn15 turkishs15 kurd6 romanian2 syrian2 kumyk1 |
| NorthCaucasus | 159 | chechen20 daghestani20 abhkasian19 georgian19 lezgin18 balkar17 adygei16 northossetian15 kumyk13 turkish1 turkishe1 |
| WestCentralAsia | 62 | chuvash16 nogay16 tajik15 turkmen10 uzbekistani4 hazara1 |
| SouthEurope | 363 | tsi98 spanish34 bulgarian31 sardinian28 french25 basque24 greek20 italians18 romanian13 cypriot12 italiann12 siciliane10 sicilianw10 tuscan8 turkishn5 turkishs5 armenian2 balkar2 turkishe2 abhkasian1 adygei1 ceu1 georgian1 |
| NorthEurope | 290 | ceu58 german30 russian25 ukrainian20 croatian19 hungarian19 norwegian18 polish17 mordovian15 orcadian15 lithuanian10 belorussian9 english8 irish7 scottish6 welsh4 french3 finnish2 armenian1 chukchi1 chuvash1 koryake1 romanian1 |
| AfghanPak | 83 | burusho25 pathan22 sindhi18 brahmin7 kshatriya5 balochi2 gond1 kalash1 meena1 uae1 |
| India | 122 | kol16 chamar10 velamas9 dharkar8 karnataka8 piramalaikallar8 dusadh7 kanjar5 muslim5 upcaste5 brahmin4 chenchu4 kurumba4 sakd4 gond3 hakkipikki3 myanmar3 kshatriya2 nihali2 tamilnadu2 tharus2 bengali1 bhunjia1 dhurwa1 kurmi1 lambadi1 malayan1 mawasi1 meghawal1 |
| Balochistan | 71 | brahui23 balochi21 makrani20 sindhi5 uae2 |
| Mongolia | 93 | buryat15 kyrgyz14 altai13 tuva13 mongolian12 daur9 oroqen7 hezhen6 burya2 nganassan1 uygur1 |
| Siberia | 54 | yakut23 evenk12 nganassan7 dolgan5 koryake2 oroqen2 yukagir2 chukchi1 |
| NorthSiberia | 25 | selkup10 chukchi3 dolgan2 ket2 koryake2 nganassan2 yakut2 yukagir2 |
| CentralAsia | 41 | hazara19 uzbekistani11 uygur9 kyrgyz2 |
| EastAsia | 175 | han34 japanese28 cambodian10 dai10 hannchina10 miao10 tu10 tujia10 yi10 she9 xibo9 mongolian7 naxi7 lahu6 naga4 hezhen1 |
| EastAfricaBantu | 109 | luhya94 bantusa8 maasai7 |
| WestAfrica | 45 | mandenka21 yoruba21 mozabite2 moroccan1 |
| EastAfricaMaasai | 90 | maasai90 |
| Ethiopia | 20 | ethiopiana7 ethiopiano7 ethiopiant5 egyptian1 |

**Table S3.** The final fineSTRUCTURE clusters used in the analysis. ClusterName refers to a unique short name given to each cluster which is named for the population that contributes the most individuals to a cluster; the Region and total number of individuals, which is also referred to in the Cluster Name are also listed. As with Table S2, the n(by population) column refers to the geographic population origin of the individuals in each cluster, with a numerical suffix representing the number of individuals from that population.

| ClusterName | Region | n(total) | n(by population) |
|---|---|---|---|
| armen2 | Armenia/Iran | 2 | armenian2 |
| armen27 | Armenia/Iran | 27 | armenian24 syrian1 turkishn1 turkishs1 |
| armen9 | Armenia/Iran | 9 | armenian2 bulgarian2 turkishe2 turkishn2 turkishs1 |
| armen6 | Armenia/Iran | 6 | armenian6 |
| irani10 | Armenia/Iran | 10 | iranian10 |
| irani5 | Armenia/Iran | 5 | iranian5 |
| kurd5 | Armenia/Iran | 5 | kurd5 |
| basqu24 | Basque | 24 | basque24 |
| chuva16 | Chuvash | 16 | chuvash16 |
| cypri12 | Cyprus | 12 | cypriot12 |
| chech19 | EastCaucasus | 19 | chechen19 |
| daghe2* | EastCaucasus | 2 | daghestani2 |
| daghe4† | EastCaucasus | 4 | daghestani4 |
| daghe10† | EastCaucasus | 10 | daghestani9 lezgin1 |
| kumyk2 | EastCaucasus | 2 | kumyk2 |
| kumyk4 | EastCaucasus | 4 | kumyk3 chechen1 |
| kumyk9 | EastCaucasus | 9 | kumyk9 |
| lezgi15 | EastCaucasus | 15 | lezgin10 daghestani5 |
| lezgi3 | EastCaucasus | 3 | lezgin3 |
| lezgi4 | EastCaucasus | 4 | lezgin4 |
| syria1 | NearEast | 1 | syrian1 |
| croat18 | NEEurope | 18 | croatian18 |
| finni3 | NEEurope | 3 | finnish2 norwegian1 |
| hunga23 | NEEurope | 23 | hungarian19 armenian1 croatian1 german1 romanian1 |
| lithu11 | NEEurope | 11 | lithuanian9 belorussian1 polish1 |
| mordo13 | NEEurope | 13 | mordovian13 |
| mordo2 | NEEurope | 2 | mordovian2 |
| russi25 | NEEurope | 25 | russian25 |
| ukrai48 | NEEurope | 48 | ukrainian20 polish16 belorussian8 chukchi1 chuvash1 koryake1 lithuanian1 |
| nogay2 | WestCentralAsia | 2 | nogay2 |
| nogay7 | WestCentralAsia | 7 | nogay7 |
| nogay7a | WestCentralAsia | 7 | nogay7 |
| tajik11 | WestCentralAsia | 11 | tajik10 turkmen1 |
| tajik3 | WestCentralAsia | 3 | tajik3 |
| turkm11 | WestCentralAsia | 11 | turkmen4 uzbekistani4 tajik2 hazara1 |
| turkm5 | WestCentralAsia | 5 | turkmen5 |
| ceu2 | NWEurope | 2 | ceu2 |
| ceu2a | NWEurope | 2 | ceu2a |
| ceu3 | NWEurope | 3 | ceu3 |
| ceu71 | NWEurope | 71 | ceu43 english8 irish7 scottish6 welsh4 french2 german1 |
| germa36 | NWEurope | 36 | german28 ceu8 |
| norwe17 | NWEurope | 17 | norwegian17 |
| orcad2 | NWEurope | 2 | orcadian2 |
| orcad2a | NWEurope | 2 | orcadian2a |
| orcad5 | NWEurope | 5 | orcadian5 |
| orcad6 | NWEurope | 6 | orcadian6 |
| sardi13 | Sardinia | 13 | sardinian13 |
| sardi6 | Sardinia | 6 | sardinian6 |
| sardi9 | Sardinia | 9 | sardinian9 |
| itali13 | SCEurope | 13 | italiann12 french1 |
| itali1 | SCEurope | 1 | italians1 |
| itali8 | SCEurope | 8 | italians8 |
| sicil30 | SCEurope | 30 | siciliane10 sicilianw10 italians9 greek1 |

Continued on next page

*Individuals from Daghestan belong to the Tabasaran ethnic group

Table S3 continued from previous page

| ClusterName | Region | n(total) | n(by population) |
|---|---|---|---|
| tsi2 | SCEurope | 2 | tsi2 |
| tsi23 | SCEurope | 23 | tsi23 |
| tsi2a | SCEurope | 2 | tsi2a |
| tsi2b | SCEurope | 2 | tsi2b |
| tsi3 | SCEurope | 3 | tsi3 |
| tsi4 | SCEurope | 4 | tsi4 |
| tsi70 | SCEurope | 70 | tsi62 tuscan8 |
| bulga46† | SEEurope | 46 | bulgarian29 romanian13 turkishs3 turkishn1 |
| greek19 | SEEurope | 19 | greek19 |
| roman2 | SEEurope | 2 | romanian2 |
| frenc24 | SWEurope | 24 | french23 ceu1 |
| spani27 | SWEurope | 27 | spanish27 |
| spani9 | SWEurope | 9 | spanish7 french2 |
| turki2 | Turkey | 2 | turkish1 turkishe1 |
| turki2a | Turkey | 2 | turkish1 turkishe1a |
| turki34 | Turkey | 34 | turkishe14 turkish13 turkishn6 turkishs1 |
| turki3 | Turkey | 3 | turkishe3 |
| turki4 | Turkey | 4 | turkishn2 turkishs2 |
| turki7 | Turkey | 7 | turkishn3 turkish2 kurd1 turkishe1 |
| turki1 | Turkey | 1 | turkishs1 |
| turki17 | Turkey | 17 | turkishs11 turkishn5 turkish1 |
| abhka16 | WestCaucasus | 16 | abhkasian15 georgian1 |
| adyge13 | WestCaucasus | 13 | adygei12 turkishe1 |
| adyge6 | WestCaucasus | 6 | adygei4 balkar1 turkish1 |
| balka16 | WestCaucasus | 16 | balkar16 |
| balka5 | WestCaucasus | 5 | balkar2 abhkasian1 adygei1 georgian1 |
| georg20 | WestCaucasus | 20 | georgian16 abhkasian4 |
| georg2 | WestCaucasus | 2 | georgian2 |
| north15 | WestCaucasus | 15 | northossetian15 |

†Turkish individuals in this cluster likely have ancestors that recently moved to Turkey from Bulgaria or Romania and are most probably of Bulgarian / Romanian origin

**Table S4.** The final results of the GLOBETROTTER analysis on 82 Eurasian Clusters. $Analysis$ refers to whether the main or masked analysis was used to produce the final result. Admixture $P$-values are based on 100 bootstrap replicates of the NULL procedure. Our resulting inference, $res$ can be: 1D (two admixing sources at a single date); 1MW (multiple admixing sources at a single date); 2D (admixture at multiple dates); NA (no-admixture); U (uncertain). max($R_1$) refers to the $R^2$ goodness-of-fit for a single date of admixture, taking the maximum value across all inferred coancestry curves. $FQ_1$ is the fit of a single admixture event (i.e. the first principal component, reflecting admixture involving two sources) and $FQ_2$ is the fit of the first two principal components capturing the admixture event(s) (the second component might be thought of as capturing a second, less strongly-signalled event. $M$ is the additional $R^2$ explained by adding a second date versus assuming only a single date of admixture; we use values above 0.35 to infer multiple dates (although see Supplementary Text for details). As well as the final result, for each event we show the inferred dates, $\alpha$s and best matching sources for 1D, 1MW, and 2D inferences. Inferred dates are in years(+ 95% CI; B=BCE, otherwise CE); the proportion of admixture from the minority source (source 1) is represented by $\alpha$. Date confidence intervals are based on 100 bootstrap replicates of the date inference (see Supplementary Experimental Procedures for details)

| Cluster | Analysis | $P$ | res | max($R_1$) | $FQ_1$ | $FQ_2$ | $M$ | $1D$ | $1D$(CI) | $1D\alpha1$ | $1D$ src1 | $1D$ src2 |
|---|---|---|---|---|---|---|---|---|---|---|---|---|
| finni3 | main | <0.01 | 1D | 0.557 | 0.995 | 1 | 0.12 | 469 | (213B-1011) | 0.12 | NorthSiberia | germa36 |
| tajik3 | ncauc | <0.01 | 1D | 0.947 | 0.996 | 1 | 0.07 | 1276 | (1135-1388) | 0.18 | EastAsia | irani10 |
| daghe4 | ecauc | <0.01 | 1MW | 0.853 | 0.954 | 0.998 | 0.06 | 1239 | (823-1442) | 0.05 | EastAsia | armen27 |
| nogay2 | ncauc | <0.01 | 2D | 0.953 | 0.999 | 1 | 0.40 | | | | | |
| nogay7a | ncauc | <0.01 | 1D(2D) | 0.97 | 0.999 | 1 | 0.70 | 1638 | (1515-1684) | 0.25 | Mongolia | balka5 |
| lezgi3 | ecauc | <0.01 | 1D | 0.827 | 0.993 | 0.998 | 0.28 | 1564 | (1001-1758) | 0.04 | Mongolia | armen27 |
| kumyk9 | ecauc | <0.01 | 1D | 0.98 | 0.995 | 0.999 | 0.28 | 1524 | (1423-1592) | 0.08 | Mongolia | armen27 |
| nogay7 | ncauc | <0.01 | 1D(2D) | 0.979 | 0.997 | 1 | 0.48 | 1479 | (1360-1564) | 0.20 | Mongolia | balka5 |
| adyge6 | wcauc | <0.01 | 1D | 0.963 | 0.99 | 1 | 0.26 | 1463 | (1299-1543) | 0.08 | Mongolia | kumyk9 |
| kumyk4 | ecauc | <0.01 | 1MW(2D) | 0.918 | 0.942 | 0.996 | 0.39 | 1443 | (1235-1580) | 0.07 | Mongolia | armen27 |
| daghe2 | ecauc | <0.01 | 1D | 0.659 | 0.981 | 0.998 | 0.35 | 1415 | (976-1734) | 0.08 | Mongolia | armen27 |
| kumyk2 | ecauc | <0.01 | 1D | 0.867 | 0.985 | 0.993 | 0.14 | 1413 | (1172-1591) | 0.07 | Mongolia | armen27 |
| turkm5 | ncauc | <0.01 | 1D | 0.966 | 0.997 | 1 | 0.33 | 1369 | (1226-1508) | 0.14 | Mongolia | irani10 |
| turki2a | turki | <0.01 | 1D | 0.898 | 1 | 1 | 0.17 | 1354 | (1146-1500) | 0.07 | Mongolia | armen27 |
| adyge13 | wcauc | <0.01 | 1MW(2D) | 0.938 | 0.969 | 0.996 | 0.44 | 1320 | (1156-1427) | 0.04 | Mongolia | turki34 |
| tajik11 | ncauc | <0.01 | 1D(2D) | 0.973 | 0.994 | 1 | 0.50 | 1295 | (1152-1374) | 0.17 | Mongolia | irani10 |
| daghe10 | ecauc | <0.01 | 1MW | 0.89 | 0.934 | 0.998 | 0.33 | 1292 | (940-1536) | 0.04 | Mongolia | armen27 |
| turkm11 | ncauc | <0.01 | 1D | 0.985 | 1 | 1 | 0.24 | 1266 | (1159-1323) | 0.27 | Mongolia | irani10 |
| turki4 | turki | <0.01 | 1D(2D) | 0.935 | 0.985 | 1 | 0.45 | 1236 | (986-1329) | 0.08 | Mongolia | sicil30 |
| turki17 | turki | <0.01 | 1D | 0.988 | 1 | 1 | 0.30 | 1213 | (1096-1272) | 0.14 | Mongolia | armen27 |
| armen9 | anat | <0.01 | 1MW(2D) | 0.954 | 0.931 | 1 | 0.57 | 1204 | (1021-1307) | 0.06 | Mongolia | bulga46 |
| lezgi15 | ecauc | <0.01 | 1MW | 0.933 | 0.901 | 0.998 | 0.12 | 1182 | (980-1322) | 0.04 | Mongolia | armen27 |
| turki34 | turki | <0.01 | 1D | 0.991 | 1 | 1 | 0.34 | 1115 | (1016-1151) | 0.08 | Mongolia | armen27 |
| chech19 | main | <0.01 | 1D | 0.953 | 0.976 | 0.999 | 0.28 | 1068 | (844-1209) | 0.11 | Mongolia | lezgi15 |
| turki3 | turki | <0.01 | 1D | 0.935 | 1 | 1 | 0.17 | 1053 | (877-1149) | 0.10 | Mongolia | armen27 |
| north15 | wcauc | <0.01 | 1D | 0.973 | 0.991 | 0.999 | 0.31 | 1020 | (866-1090) | 0.08 | Mongolia | kumyk9 |
| lezgi4 | ecauc | <0.01 | 1MW | 0.761 | 0.914 | 0.993 | 0.09 | 992 | (529-1282) | 0.06 | Mongolia | armen27 |
| russi25 | main | <0.01 | 1D | 0.958 | 0.999 | 1 | 0.30 | 913 | (754-1007) | 0.10 | Mongolia | ukrai48 |
| balka16 | wcauc | <0.01 | 1D | 0.968 | 0.997 | 0.999 | 0.17 | 901 | (748-976) | 0.08 | Mongolia | armen27 |
| chuva16 | main | <0.01 | 1D | 0.968 | 1 | 1 | 0.33 | 829 | (627-940) | 0.22 | Mongolia | ukrai48 |
| mordo13 | main | <0.01 | 1D | 0.927 | 1 | 1 | 0.20 | 792 | (564-975) | 0.07 | Mongolia | ukrai48 |
| irani10 | main | <0.01 | 1MW | 0.942 | 0.96 | 0.989 | 0.30 | 1221 | (1063-1330) | 0.09 | CentralAsia | turki7 |
| balka5 | wcauc | <0.01 | 1MW | 0.912 | 0.867 | 1 | 0.22 | 1177 | (876-1389) | 0.16 | CentralAsia | bulga46 |
| roman2 | main | <0.01 | 1D | 0.815 | 1 | 1 | 0.10 | 990 | (741-1245) | 0.34 | India | bulga46 |
| frenc24 | main | <0.01 | 1D | 0.777 | 0.993 | 0.998 | 0.18 | 846 | (424-1011) | 0.12 | Levant | ceu71 |
| sardi6 | sard | <0.01 | 1D | 0.631 | 0.993 | 1 | 0.05 | 449 | (40-786) | 0.07 | Levant | itali13 |
| tsi23 | italy | <0.01 | 1D | 0.744 | 0.999 | 1 | 0.08 | 400 | (33B-686) | 0.29 | Levant | frenc24 |
| itali8 | main | <0.01 | 1D | 0.73 | 0.988 | 1 | 0.07 | 295 | (72B-604) | 0.34 | Levant | itali13 |
| armen27 | anat | <0.01 | 1MW | 0.548 | 0.958 | 0.993 | 0.04 | 363B | (1085B-382) | 0.36 | Levant | kumyk9 |
| sardi9 | sard | <0.01 | 1D | 0.74 | 0.996 | 1 | 0.06 | 356 | (214B-736) | 0.09 | NorthAfricaII | itali13 |
| irani5 | anat | <0.01 | 1D(2D) | 0.955 | 0.994 | 1 | 0.37 | 1412 | (1054-1526) | 0.03 | EastAfricaBantu | turki7 |
| sicil30 | italy | <0.01 | 1D(2D) | 0.961 | 0.984 | 1 | 0.50 | 1105 | (882-1250) | 0.05 | WestAfrica | greek19 |
| spani27 | spain | <0.01 | 1MW(2D) | 0.961 | 0.907 | 0.999 | 0.47 | 1042 | (740-1201) | 0.07 | WestAfrica | frenc24 |
| basqu24 | main | <0.01 | 1MW | 0.722 | 0.703 | 0.997 | 0.06 | 886 | (283-1162) | 0.01 | WestAfrica | spani9 |
| kurd5 | anat | <0.01 | 1D | 0.812 | 0.994 | 0.999 | 0.05 | 872 | (312-1069) | 0.01 | WestAfrica | turki7 |
| cypri12 | main | <0.01 | 1MW | 0.906 | 0.971 | 0.999 | 0.06 | 427 | (107-734) | 0.03 | WestAfrica | armen27 |
| sardi13 | sard | <0.01 | 1D | 0.869 | 0.983 | 0.999 | 0.07 | 36 | (471B-374) | 0.02 | WestAfrica | itali13 |
| hunga23 | main | <0.01 | 1D | 0.888 | 0.999 | 1 | 0.27 | 1150 | (948-1326) | 0.43 | sicil30 | lithu11 |
| germa36 | main | <0.01 | 1D | 0.711 | 0.995 | 1 | 0.07 | 969 | (778-1160) | 0.41 | lithu11 | frenc24 |
| bulga46 | main | <0.01 | 1D | 0.944 | 1 | 1 | 0.22 | 968 | (800-1028) | 0.49 | armen27 | lithu11 |
| ukrai48 | main | <0.01 | 1D | 0.934 | 0.988 | 1 | 0.26 | 899 | (726-1025) | 0.17 | turki34 | lithu11 |
| croat18 | main | <0.01 | 1D | 0.85 | 1 | 1 | 0.03 | 708 | (492-877) | 0.34 | armen27 | lithu11 |
| greek19 | main | <0.01 | 1D | 0.837 | 0.999 | 1 | 0.11 | 630 | (280-781) | 0.35 | lithu11 | cypri12 |
| norwe17 | main | <0.01 | 1D | 0.539 | 0.998 | 1 | 0.06 | 351 | (262B-893) | 0.08 | mordo13 | ceu71 |
| lithu11 | slav | <0.01 | 1MW | 0.331 | 0.962 | 0.994 | 0.01 | 85 | (1240B-623) | 0.22 | russi25 | hunga23 |
| spani9 | spain | <0.01 | 1MW | 0.725 | 0.844 | 0.999 | 0.08 | 668 | (286-876) | 0.19 | basqu24 | itali13 |
| orcad5 | orcad | <0.01 | 1D | 0.322 | 0.998 | 0.999 | 0.06 | 1241 | (876-1442) | 0.14 | sicil30 | norwe17 |
| ceu71 | main | <0.01 | 1MW(2D) | 0.81 | 0.962 | 0.994 | 0.61 | 858 | (467-1224) | 0.11 | sicil30 | germa36 |
| turki7 | turki | <0.01 | 1MW | 0.526 | 0.965 | 0.994 | 0.04 | 1015 | (561-1234) | 0.47 | armen27 | kurd5 |
| orcad6 | orcad | <0.01 | 1D | 0.282 | 0.987 | 0.996 | 0.05 | 708 | (65B-1421) | 0.37 | armen9 | norwe17 |
| itali13 | italy | <0.01 | 1D | 0.606 | 0.986 | 1 | 0.04 | 677 | (362-989) | 0.33 | cypri12 | frenc24 |
| tsi70 | italy | <0.01 | 1D | 0.901 | 0.989 | 1 | 0.08 | 241 | (16B-417) | 0.42 | cypri12 | ceu71 |
| georg20 | wcauc | <0.01 | 1D | 0.763 | 0.995 | 0.998 | 0.16 | 1055 | (603-1321) | 0.17 | nogay7 | armen27 |
| abhka16 | wcauc | <0.01 | 1D | 0.889 | 0.996 | 0.999 | 0.34 | 1147 | (906-1274) | 0.22 | nogay7a | armen27 |
| syria1 | main | 1 | NA | 0.876 | 0.969 | 1 | 0.18 | 1080 | (1051-1051) | 0.11 | Ethiopia | balka16 |
| turki2 | turki | 1 | NA | 0.845 | 1 | 1 | 0.02 | 958 | | 0.07 | Mongolia | armen27 |
| orcad2a | main | 1 | NA | | | | | | | 1.00 | orcad5 | |
| orcad2 | main | 1 | NA | | | | | | | 1.00 | orcad5 | |

| Cluster | $1MW$ $\alpha$ | $1MW$ src3 | $1MW$ src4 | $2D1$ | $2D1$ (CI) | $2D1\alpha$ | $2D1$ src1 | $2D1$ src2 | $2D2$ | $2D1$(CI) | $2D2\alpha$ | $2D2$ src1 | $2D2$ src2 |
|---|---|---|---|---|---|---|---|---|---|---|---|---|---|
| daghe4 | 0.48 | armen9 | turki34 | | | | | | | | | | |
| nogay2 | | | | 1761 | (1621-1815) | 0.25 | Mongolia | adyge6 | 173B | (1541B-478) | 0.31 | Mongolia | balka5 |
| kumyk4 | 0.50 | north15 | armen9 | | | | | | | | | | |
| adyge13 | 0.36 | armen9 | kumyk9 | | | | | | | | | | |
| daghe10 | 0.36 | armen9 | turki34 | | | | | | | | | | |
| armen9 | 0.49 | ukrai48 | turki7 | | | | | | | | | | |
| lezgi15 | 0.35 | armen9 | turki34 | | | | | | | | | | |
| lezgi4 | 0.49 | turki34 | armen9 | | | | | | | | | | |
| irani10 | 0.34 | Levant | turki17 | | | | | | | | | | |
| balka5 | 0.36 | mordo13 | armen27 | | | | | | | | | | |
| armen27 | 0.35 | georg20 | turki7 | | | | | | | | | | |
| spani27 | 0.22 | basqu24 | itali13 | | | | | | | | | | |
| basqu24 | 0.42 | germa36 | spani27 | | | | | | | | | | |
| cypri12 | 0.23 | hunga23 | armen27 | | | | | | | | | | |
| lithu11 | 0.41 | orcad5 | russi25 | | | | | | | | | | |
| spani9 | 0.15 | Levant | frenc24 | | | | | | | | | | |
| ceu71 | 0.44 | itali13 | norwe17 | | | | | | | | | | |
| turki7 | 0.09 | CentralAsia | kurd5 | | | | | | | | | | |
| syria1 | 0.47 | balka16 | cypri12 | 1835 | | 0.49 | balka16 | armen27 | 1048 | | 0.03 | WestAfrica | armen27 |
| turki2 | 0.28 | hunga23 | armen27 | 1038 | | 0.06 | Mongolia | armen27 | 597 | | 0.13 | Siberia | cypri12 |

Continued on next page

**Table S4 Continued from previous page**

| Cluster | Analysis | $P$ | res | max($R_1$) | $FQ_1$ | $FQ_2$ | $M$ | $1D$ | $1D$(CI) | $1D\alpha1$ | $1D$ src1 | $1D$ src2 | $1MW$ $\alpha$ | $1MW$ src3 | $1MW$ src4 | $2D1$ | $2D1$ (CI) | $2D1\alpha$ | $2D1$ src1 | $2D1$ src2 | $2D2$ | $2D1$(CI) | $2D2\alpha$ | $2D2$ src1 | $2D2$ src2 |
|---|---|---|---|---|---|---|---|---|---|---|---|---|---|---|---|---|---|---|---|---|---|---|---|---|---|
| itali1 | italy | 1 | NA | 0.409 | 0.939 | 0.996 | 0.04 | 1183 | (1154-1154) | 0.40 | sardi13 | cypri12 | 0.17 | sardi9 | greek19 | 1350 | | 0.4 | sardi13 | armen9 | 162 | | 0.18 | NorthAfricaII | spani9 |
| turki1 | turki | 1 | NA | 0.959 | 0.954 | 1 | 0.73 | 1803 | (1774-1774) | 0.07 | WestAfrica | kumyk9 | 0.23 | CentralAsia | cypri12 | 1870 | (1841-1841) | 0.07 | WestAfrica | kumyk9 | 1137 | (1108-1108) | 0.13 | Mongolia | cypri12 |
| tsi2 | main | <0.01 | U | 0.404 | 0.928 | 1 | 0.04 | 582 | (29-964) | 0.14 | balka16 | tsi23 | 0.45 | hunga23 | tsi23 | 1318 | | 0.24 | balka5 | tsi23 | 550 | | 0.1 | Levant | tsi23 |
| ceu2a | main | <0.01 | U | 0.484 | 0.996 | 0.998 | 0.04 | 1010 | (423B-1429) | 0.10 | finni3 | ceu71 | 0.32 | frenc24 | ceu71 | 1625 | | 0.46 | frenc24 | orcad6 | 1162 | | 0.12 | hunga23 | ceu71 |
| armen6 | main | <0.01 | U | 0.134 | 0.987 | 0.998 | 0.03 | 143B | (2881B-1326) | 0.42 | georg20 | sicil30 | 0.41 | irani5 | greek19 | 1344 | | 0.31 | armen9 | armen27 | 1604B | | 0.43 | abhka16 | cypri12 |
| georg2 | main | <0.01 | U | 0.622 | 0.999 | 1 | 0.19 | 1785 | (1605-1853) | 0.27 | hunga23 | georg20 | 0.13 | nogay7 | georg20 | 1908 | | 0.28 | ukrai48 | georg20 | 763 | | 0.2 | mordo13 | georg20 |
| tsi2a | main | <0.01 | U | 0.286 | 0.999 | 1 | 0.13 | 907B | (1946B-565) | 0.39 | Levant | norwe17 | 0.37 | mordo13 | tsi23 | 1619 | | 0.41 | frenc24 | tsi23 | 494B | | 0.16 | Levant | frenc24 |
| tsi4 | main | <0.01 | U | 0.357 | 0.991 | 0.999 | 0.11 | 91 | (688B-569) | 0.16 | Levant | tsi23 | 0.39 | mordo13 | tsi23 | 1580 | | 0.38 | mordo13 | tsi23 | 410 | | 0.06 | NorthAfricaII | tsi23 |
| tsi3 | main | <0.01 | U | 0.277 | 0.985 | 0.997 | 0.03 | 208 | (315B-693) | 0.23 | Levant | tsi23 | 0.45 | balka5 | tsi23 | 1547 | | 0.42 | armen9 | tsi23 | 7B | | 0.22 | Levant | tsi23 |
| armen2 | main | <0.01 | U | 0.155 | 0.917 | 0.969 | 0.04 | 1594 | (691-1892) | 0.15 | Levant | armen27 | 0.48 | armen27 | armen27 | 1892 | | 0.44 | armen27 | irani5 | 1649 | | 0.16 | Levant | armen27 |
| mordo2 | main | <0.01 | U | 0.583 | 0.924 | 0.996 | 0.08 | 558 | (179-843) | 0.12 | Mongolia | ukrai48 | 0.30 | finni3 | mordo13 | 1804 | | 0.28 | finni3 | mordo13 | 804 | | 0.05 | Mongolia | mordo13 |
| ceu2 | main | 0.59 | U | 0.0523 | 1 | 1 | 0.01 | 1825 | (10949B-1892) | 0.06 | orcad5 | ceu71 | 0.37 | orcad6 | ceu71 | 1869 | | 0.06 | orcad5 | ceu71 | 12008B | | 0.5 | bulga46 | orcad5 |
| tsi2b | main | <0.01 | U | 0.491 | 0.993 | 0.999 | 0.09 | 1512 | (1133-1670) | 0.31 | spani27 | tsi23 | 0.04 | Levant | tsi23 | 1679 | | 0.26 | spani27 | tsi23 | 1250 | | 0.39 | orcad5 | tsi70 |
| ceu3 | main | <0.01 | U | 0.108 | 0.936 | 0.973 | 0.03 | 347B | (2002B-1892) | 0.32 | spani27 | finni3 | 0.48 | orcad5 | frenc24 | 1921 | | 0.14 | spani27 | ceu71 | 1237B | | 0.35 | sardi9 | finni3 |

**Table S5.** Results of All GLOBETROTTER Analyses Performed on 82 Eurasian Clusters, Related to Figure 4. Column headings are as in Table S4. All clusters were analyzed once using all clusters with at least five individuals as surrogates (main analysis). Others were also analyzed with bespoke masked analyses, as noted in the main text. The NULL procedure was run for each of these analyses for comparative purposes.

# Supplemental Experimental Procedures

# S1 Dataset and identification of genetic populations

## S1.1 Outlier removal with PLINK IBD and PCA

We used pairwise IBD analysis to remove potentially related individuals from further analysis. Using PLINK, we compared the proportion of SNPs that were IBD for each individual in a population with every other individual from their population using the pi-hat statistic. Evolutionary history, genetic drift, and relative isolation will lead to differences in the background level of IBD in different populations. To take this into account, we chose a variable threshold of exclusion for putatively related individuals. We used the larger value of the 97.5% quantile of population IBD or 0.2, as the threshold for exclusion, and for all pairs above this threshold, dropped the individual with the lower genotyping rate from further analysis. We next used smartpca in the EIGENSOFT package [S1] to perform PCA on the pruned dataset. Individuals were split into broad geographical regions (Europe; Near East; Asia; Africa) and separate runs of the program were performed. Each region contained individuals typed from different studies on different chips, and visual inspection of PC1 v PC2 plots allowed a qualitative assessment of the merging process. For European populations, smartpca was run with no outlier iterations and all individuals were kept for futher analysis; for the remaining three regions, three outlier iterations were performed, with the resulting outliers dropped from the full analysis. The final dataset contained 2,192 individuals from 144 populations typed on 477,812 SNPs (Table S1).

## S1.2 Phasing

We used SHAPEITv1 [S2] to phase the data. SHAPEITv1 conditions the underlying hidden Markov model (HMM) from ref. [S3] on all available haplotypes to quickly estimate haplotypic phase from genotype data. We split our dataset (in binary PLINK format) by chromosome and phased all individuals simulataneously, and used the most likely pairs of haplotypes (using the *–output-max* option) for each individual for downstream applications. Based on the numbers of individuals from Europe, Asia and Africa in our dataset and the instructions of SHAPEITv1 available at the time of phasing, we used an estimated effective population size of 13,887. (1189 Asians; Asian Ne: 14,269; 334 Africans; African Ne 17,469; 669 Europeans; European Ne: 11,418.)

## S1.3 Visualising broad-scale population structure with ADMIXTURE

We used ADMIXTURE [S4] to describe the genome-wide ancestry of our dataset and by way of comparison of our method to commonly used procedures (Figure S2). We thinned the dataset to remove the effect of LD by removing SNPs whose pairwise correlation across the sample was greater than 0.2. This thinned dataset contained 135,101 SNPs, and ADMIXTURE was run for all values of $K \in [2, ..., 14]$.

## S1.4 Rationale for using genomic data to group individuals

The major aim of our paper is to explore the different ancestral contributions to different “groups” of individuals across Western Eurasia. Often, researchers group individuals based on labels associated with geography (e.g. population), or less often a cultural label such as religion, language or life history. Whilst this is generally satisfactory, it is far from perfect. What constitutes a “population” has been, and still is, a topic of much debate [S5]. However, we nevertheless believe that it is important to consider how to split a dataset into constituent groups when investigating human evolutionary history. Take Italy

as an example. Figure S1A is a PCA of Europe with different population labels assigned to individuals, which are each represented by a point, who all fall under the general geographic descriptive label of Italy (HAPMAP TSI individuals are included in the PCA, but are not coloured). From this plot it is clear that Northern and Southern Italians are genetically distinct, which may potentially be due to different ancestries. Sardinia and Sicily are Italian islands, so fall under the geographic population label of Italy, but (as has been known for a long time [S6]) Sardinians are clearly distinct from the rest of Italy, whilst for Sicilians and Southern Italians it is not as clear-cut. Whilst this sort of difference is not necessarily present across all European countries, this example serves to show us that geographic labels can be improved upon. With this in mind, we constructed new groups of individuals based on an objective assessment of genetic similarity alone.

## S1.5 Grouping individuals on the basis of genetic similarity

We used an inferential framework for investigating population structure from haplotypic data [S7]. Initially, haplotypic chromosomes are "painted" sequentially using an updated implementation of a model initially introduced by Li & Stephens [S3] and which is exploited by the CHROMOPAINTER package [S7]. The Li & Stephens copying model explicitly relates linkage disequilibrium to the underlying recombination process and CHROMOPAINTER uses an approximate method to reconstruct each "recipient" individual's haplotypic genome as a series of recombination "chunks" from a set of sample "donor" individuals. The aim of this approach is to identify, at each SNP as we move along the genome, the closest relative genome among the members of the donor sample. Because of recombination, the identity of the closest relative will change depending on the admixture history between individual genomes. Even distantly related populations share some genetic ancestry since most human genetic variation is shared, [S8,S9] but the amount of shared ancestry can differ widely. We use the term "painting" here to refer to the application of a different label to each of the donors, such that – conceptually – each donor is represented by a different colour. Donors may be coloured individually, or in groups based on *a priori* defined labels, such as the geographic population that they come from. By recovering the changing identity of the closest ancestor along chromosomes we can understand the varying contributions of different donor groups to a given population, and by understanding the distribution of these chunks we can begin to uncover the historical relationships between groups.

CHROMOPAINTER reconstructs each recipient individual's haplotypes as mosaics of a set of donors and efficiently summarises this ancestry painting in the form of a 'copying vector', which is the total proportion of genome-wide DNA (either by total expected number of shared chunks, or total expected length of chunks) copied from each labelled donor group (i.e. colour). These copying vectors contain a rich summary of the (genome-wide) relationships between individuals, and similarities in these vectors imply shared ancestral history [S7]. We therefore decided to use these copying vectors and fineSTRUCTURE to group individuals based on this measurement of shared ancestral history.

## S1.6 Using CHROMOPAINTER to generate a 'coancestry matrix' for fineSTRUCTURE

### S1.6.1 Estimating Ne and $\theta$ using Expectation-Maximisation

We performed an initial CHROMOPAINTER run to estimate the two nuisance parameters, Ne and $\theta$ using the expectation-maximisation (EM) option. (These are not Ne and $\theta$ in the traditional sense but refer to parameters from the Li and Stephens model that are used to estimate the recombination rate distribution underlying the model.) We note the process of estimating them here and their values for reproducibility. The EM algorithm iterates over the data to find the local optimum values for these parameters, given the data. Ne is the 'recombination scaling constant' and is directly related to the effective

population size. It is used by CHROMOPAINTER to convert the values of the genetic distance between SNPs (taken from the genetic map) to the population-scaled values for these distances required by the algorithm. We used the human genome build 36 genetic map downloaded from the HAPMAP website (http://hapmap.ncbi.nlm.nih.gov/downloads/recombination/). $\theta$ is the per site mutation rate parameter and is used by the CHROMOPAINTER HMM to allow for imperfect copying between haplotypes.

We used CHROMOPAINTER with 10 Expectation-Maximisation (E-M) steps to jointly estimate the program's parameters Ne and $\theta$, repeating this separately for chromosomes 1, 5, 8, 12, 17, 22 and weight-averaging (using centimorgan sizes) the Ne and $\theta$ from the final E-M step across the six chromosomes with the following command line:

```
chromopainter -g <infile> -r <recomrates> -f <donorfile> -o <outfile>
        -a 0 0 -s 0 -i 10 -in -iM
```

Due to the exhaustive nature of this estimation, we averaged these values across a subset of populations (Armenian, Bedouin, Bulgarian, German, Mandenka, Mozabite, Palestinian). We used the global values estimated from these populations of 318.8 and 0.0002 for Ne (-n flag) and $\theta$ (-M flag) respectively

## S1.7 Using the coancestry matrix to identify clusters with fineSTRUCTURE

We then ran CHROMOPAINTER on each chromosome separately for each individual as a recipient, using every other individual (i.e. 2191) as donors with the following command line:

```
chromopainter -g <infile> -r <recomrates> -f <donorfile> -o <outfile>
        -a 0 0 -s 0 -M 0.0002 -n 318.8
```

This generated a 2192 v 2192 matrix of copying vectors that was then passed to fineSTRUCTURE to cluster individuals on the basis of the similarities of these copying vectors. fineSTRUCTURE is a model based Bayesian clustering algorithm that efficiently uses the output of CHROMOPAINTER to identify population structure. Individuals are initially assumed to have independent ancestry proportions (i.e. copying vectors), but because historical relationships among individuals result in correlations in their copying vectors, individuals can be grouped together on the basis of this similarity. At each iteration, a series of splits and merges are performed on random samples of individuals, such that clusters with higher partition probability are kept at the end of each iteration. We ran fineSTRUCTURE for 10 million iterations, sampling every 10,000, using the following command:

```
finestructure -X -Y -x 0 -y 1e7 -z 1e5 <chunkcounts> <mcmcfile>
```

As the aim of this step was to group individuals into major world regions, we checked pairwise coincidence across all 10 million iterations. Pairwise coincidence (i.e. the number of times a pair of individuals are placed in the same cluster) within and across the two runs was high indicating that individuals tended to occur in the same clusters across all 10 million iterations of the algorithm.

We used the fineSTRUCTURE run with the highest posterior probability to produce a tree relating these clusters by running the maximum a posteriori (MAP) state from the initial run and used 10 million iterations of the tree building model and a very large value for the *maxtreestates* (t) option, to ensure that a large number of trees were considered at each iteration, using the following command:

```
finestructure -X -Y -m T -t 1e8 -x 1e6 <chunkcounts> <mcmcfile> <treefile>
```

To find the tree, fineSTRUCTURE starts from the MAP state and successively merges clusters, choosing the merge giving the highest probability for the merge at each step, which results in a bifurcating tree

relating the clusters [S7]. Bipartition uncertainties are produced for the nodes of the tree, which are the proportion of MCMC samples for which all individuals on one side of the split merge with each other prior to merging with any individual from the other side of the split.

## S1.8 Identifying donor World Regions using fineSTRUCTURE

We used the full fineSTRUCTURE analysis to group individuals based on their position in the tree (Fig. S1C). We found 301 clusters in total and visually inspected the tree to identify 22 major clades on the tree and successively merged all non-European clusters to reduce the number from 2192 to 22 world-wide clades, containing individuals that broadly matched geographical regions of the world, and that we use to represent the donor genomes of these different regions (Table S2; Fig. S1B).

The purpose of this step was to identify fineSTRUCTURE "continents" (which we term "world regions") to be used for a second run of the algorithm. The processing time of the algorithm is directly related to the number of individuals included in the analysis, so reducing the number of individuals speeds up the analysis. Furthermore, fineSTRUCTURE initially uses a prior that assumes that all individuals are equally distant from each other, which in the case of worldwide populations is likely to be untrue: European populations are more closely related to each other than to African populations, for example. The result is that not all of the substructure is identified in one run. We therefore generated a set of world-regions, which combine all of the copying vectors from the individuals within them to look like (re-weighted) normal individuals but cannot be split and do not contribute to parameter inference, and can thus be considered as copying vectors that contain the average of the individuals within them. They can therefore be included in the algorithm at minimal extra computational cost and exist primarily to provide chunks to (and from) the remaining groups. We additionally use these world regions, together with the Eurasian clusters, throughout the subsequent analysis as our donor (and in the case of the Eurasian clusters recipient) groups in the GLOBETROTTER analysis. Noting that the North African World Region contained the drifted Mozabites together with Tunisians, and Moroccans, we split the North Africa World Region into two, keeping the Mozabites separate from the other North Africans.

## S1.9 Identifying the final Eurasian clusters using fineSTRUCTURE

We performed 2 runs of the fineSTRUCTURE algorithm using the 18 non-Eurasian world regions as 'continents' with the 1000 Eurasian individuals belonging to the 5 world regions (Anatolia, SouthCaucasus, NorthCaucasus, SouthEurope, NorthEurope), using the following command:

```
finestructure -X -Y -m T -t 10e6 -x 1e6 -F <world_regions> <chunkcounts>
        <mcmcfile> <treefile>
```

The two runs of fineSTRUCTURE gave very similar results, inferring 82 and 83 clusters. 74 of the clusters were identical, with the remaining clusters containing cases where at most two individuals were swapped between clusters. Given the high similarity between the clusters, we chose the run with the highest posterior probability containing 100 clusters (82 Eurasian plus 18 world regions) as the final groups for the analysis (Table S3). We use these 82 clusters with GLOBETROTTER to assess the presence of admixture across Europe, and subsequently to infer the proportion, timing, and identity of admixture in these groups.

## S1.10 Results of the fineSTRUCTURE analysis

Figure S1 shows summary results of the fineSTRUCTURE analysis. The heatmap clearly suggests that different European groups copy different amounts of their genome from different parts of the world.

These differences are subtle however, and are often not much more than a few percent. The aim of our analysis is to use GLOBETROTTER to try to explain these differences and to assess the evidence that genetic admixture may have caused them.

## S1.11 Principal components analysis of the CHROMOPAINTER coancestry matrix

As final justification for the use of fineSTRUCTURE clusters we show a visualisation of a principal components of the CHROMOPAINTER chunklengths coancestry matrix (Fig. S4B-D). The PCA is somewhat similar to PCAs based on genotype data [S10,S11] although the inclusion of Caucasus and Turkish individuals causes the plot to differ from the familiar map of Europe. The points are labelled with the same labels as Figure 1 in the main paper (which is also reproduced in the legend). Whilst generally individuals with the same label (i.e. from the same fineSTRUCTURE cluster) appear close to each other on the plot, some clusters are more spread out. The first two (ten) PCs explain 14.4% (35.4%) of the variance in the data suggesting that not all variation described by the clustering is captured by the highest PCs.

## S2 Description of ADMIXTURE analysis

We used ADMIXTURE [S4] to initially survey the dataset for evidence of admixture. We performed 10 independent runs of the algorithm on the pruned dataset of 132K SNPs (see Experimental Procedures), with different randomly selected seeds at all values of $K \in [2, ..., 14]$. Multiple runs at the same $K$ were merged with CLUMPP [S12] using 10,000 iterations of the LargeKGreedy option. Figure S2 shows the results of this analysis, with bars showing the averaged admixture proportions from each of the $K$ inferred groups across all individuals from a given cluster or world region. Admixture is largely visible in all clusters (and some of the world regions) at higher values of $K$.

In particular, West Eurasian populations tend to be some mixture of 3 main ancestral components (dark blue, dark green, light green), with clear examples of admixture from components outside of these main three (for example the light blue 'Middle Eastern' component present in most European groups), with the Caucasus tending to be a mixture of the same components, with a comparatively larger amount of dark green ancestry, compare to the dark blue/light green of Europe.

# S3 GLOBETROTTER: description of method to infer admixture

## S3.1 The GLOBETROTTER pipeline

We used GLOBETROTTER to characterise admixture in Eurasian populations. This procedure aims to identify and date admixture among clusters in our dataset, as well as identify the admixing groups involved and the proportions of DNA contributed from each group. The analysis presented here follows closely that of Hellenthal et al [S13] with the key difference that we (a) initially use fineSTRUCTURE to group individuals based on genetics alone; and (b) combine non-Eurasian individuals into broad geographic "world regions" as outlined above. We describe some of the testing and validation performed previously in Section S3.2 below.

To run GLOBETROTTER, we must generate a copying vector for each donor group and a set of painted chromosomes for each recipient group.

#### Using CHROMOPAINTER to generate DONOR copying vectors for the 18 non-Eurasian world regions and 82 Eurasian clusters

To estimate a set of copying vectors ($f^i$ in the notation of Hellenthal et al [S13]) for $j$ donor groups, for each recipient cluster $k$ we used the original coancestry matrix (where we painted all 2192 with every other) and summed the contributions from each individual in each of the $j \neq k \in [1, ..., K]$ non-recipient groups separately (i.e. 18 non-West Eurasian world regions and 82 West Eurasian clusters), producing a $j \neq k \in [1, ..., K]$ element copying vector for each donor group in the analysis. Note that we therefore generate a separate set of copying vectors for each recipient group, describing the amount of copying from each $j \neq k$ donor groups to that recipient group. In practice, this meant that we estimated the mean copying from each of the $j \neq k$ (i.e. 99) donor groups across all individuals in each recipient group to produce a single donor copying-vector for each of the 100 groups in the analysis.

#### Using CHROMOPAINTER to generate painting samples of the 82 RECIPIENT Eurasian clusters

We calculated cluster-specific values of $N_e$ and $\theta$ by performing a 'leave-one-out' procedure where each individual from a given cluster $k$ is allowed to copy from every other individual with the same cluster label and $n_j - 1$ randomly chosen individuals from each donor cluster $j \neq k \in [1, ..., K]$ ($n_j$ is the number of individuals with cluster label $j$). Because then there are $n_j$ samples to copy from each cluster $j \neq k \in [1, ..., K]$, while only $n_k - 1$ samples to copy from their own cluster (as they cannot be used to paint them-self), we avoid this reduction by 1 causing problems later, by instead removing one individual from each of the other clusters $j \neq k \in [1, ..., K]$ when painting. Thus all individuals in the dataset copy from the same number of individuals from each labelled cluster ensuring that each individual from each cluster copies from the exact same number of individuals from every other cluster label including their own.

Accounting for the fact that each recipient group is now copying from a different (i.e. $j \neq k$) set of donors, we used the cluster-specific values of Ne and $\theta$ calculated above, and re-ran CHROMOPAINTER for each of the $K$ (82) Eurasian clusters with all individuals from the other $j \neq k$ clusters and World Regions (99) as donors, this time without allowing individuals to copy from other individuals in the same cluster, and generating 10 painting samples for each recipient cluster.

```
chromopainter -g <infile> -r <recomrates> -f <donorfile> -o <outfile>
              -s 10 -M mu -n Ne
```

## S3.2 Characterising admixture events

To test the robustness of the admixture inference, we use the idea that in truly admixed populations, ancestry segments producing "admixture LD" occur within individual genomes, resulting in ancestry LD characteristically decaying within individual genomes much more strongly than when ancestry is measured in different individuals. To construct a test of this based on our method's inference of genetic ancestry, we first generate "across-individual" coancestry curves, by considering CHROMOPAINTER painting samples from different individuals (using the NULL procedure outlined in the GLOBETROTTER manual and here [S13]), and use these to normalise our original coancestry curves. We re-infer admixture using this "NULL" individual, generating 100 date bootstraps, and test to see if the results are different from the non-NULL inference. Specifically, we assess evidence of any admixture, by obtaining an empirical $P$-value as $P$ = $D$/101, where $D$ is the number of NULL date bootstraps with a date $\lambda$, where $\lambda \leq 1$ or $\lambda \geq 400$, rejecting the null of no admixture only if $P < 0.01$. We also compare how well the modelled date of admixture (i.e. the coancestry curve $R^2$) changes between the normal and NULL run of the algorithm, and reject the null of no-admixture only when this reduction is less than 1/3. In practice, this meant that we have little confidence in admixture inference in most clusters containing small numbers of individuals ($n$ <4). Admixture in such clusters is defined as uncertain (U) or, when $P$ >0.01, as no-admixture (NA).

A key aspect of GLOBETROTTER is that we do not need to identify *a priori* the admixing source groups: all that is needed is a set of surrogate groups from which to infer admixture events. Moreover, this approach allows us to define source groups as mixtures of these available surrogate groups. We ran GLOBETROTTER under a number of different scenarios, changing the identity of the available donor groups (outlined in full in Supplemental Experimental Procedures Section S4). We note that in general GLOBETROTTER will identify the most recent admixture event.

## S3.3 Validation of GLOBETROTTER

We reported the full description of the GLOBETROTTER method, and our extensive testing of its robustness to different conditions in real and simulated data, in Hellenthal et al [S13] and its associated Supplementary Material. We also conducted comparisons of GLOBETROTTER to other available methods, such as ROLLOFF and ADMIXTURE, in that publication. We re-iterate here several key aspects of the validation we performed.

### S3.3.1 GLOBETROTTER simulations undertaken by Hellenthal et al 2014

To test GLOBETROTTER under diverse single, complex, and no-admixture scenarios, incorporating many of the complexities (such as unsampled or admixed donor groups) likely to be present in real data, we simulated admixture scenarios involving real (but hidden to our analysis) human populations [S13] and populations generated under a coalescent framework [S14] incorporating inferred [S15–18] past demographic events.

Admixture was simulated between 7 and 160 generations [200 to 4400 years] ago, with admixture fractions 3 to 50% and genetic differentiation ($F_{ST}$) between the admixing groups varying from 0.018 (similar to Europe versus Central Asia) to 0.185 (similar to West Africa versus Europe). Results are detailed in Hellenthal et al 2014 [S13]. All populations simulated without admixture, including those with long-term migration, showed no admixture evidence ($P$ >0.1). Power to detect admixture ($P$ <0.01) when present was 94%, and 95% of our 95% bootstrapped confidence intervals (CIs) contained the true admixture date, including cases with two distinct incidents of admixture or multiple groups admixing simultaneously.

In our simulations, inferred source accuracy was very high, with, for example, the mixture representation predicting a haplotype composition more correlated to the true, typically unsampled, source

population than to any single sampled population >80% of the time. However, source accuracy was lower for admixing sources contributing only 5% of DNA, with around 40% of such scenarios yielding elevated (>25%) rates of falsely inferring multiple admixture times and/or admixing groups.

Further testing demonstrated robustness of GLOBETROTTER, in simulations and real data, to haplotypic phase inference approach used, inclusion/exclusion of particular chromosomes, genetic map chosen to provide genetic distances, and the presence of population bottlenecks since admixture, whereas GLOBETROTTER admixture dating was improved relative to ROLLOFF [S19].

Even so, there are multiple settings that we believe are challenging for GLOBETROTTER:

1. although the admixing sources need not be sampled—often impossible because of genetic drift, extinction, or later admixture into the sources themselves—source inference is improved when more similar extant groups are sampled, and GLOBETROTTER may miss events where we lack any extant group that can separate sources.

2. sampling of several genetically very similar groups can mask admixture events they share. Similarly, a caveat is that where genuine, recent bidirectional gene flow has occurred, admixture fractions are difficult to define and interpret. However, date estimation is predicted to still be useful, and in real data the majority of our inferred events do not appear to be bidirectional in this manner.

3. even in theory our approach finds it challenging to distinguish distinct continuous "pulses" of admixture and continuous migration over some time frame, because of the difficulty of separating exponential mixtures [S20]. If the time frame were narrow, we expect to infer a single admixture time within the range of migration dates. Where we infer two admixture dates, in particular with the same source groups, the exponential decay signal could also be consistent with more continuous migration, and so we conservatively refer to this as admixture at multiple dates.

4. we only attempt to analyze populations with signals consistent with at most three groups admixing and infer at most two admixture times, and we can provide only less precise inference of sources for the weaker or older admixture signal in these complex cases.

#### S3.3.2 Other considerations

We note from the above that the presence of admixed donor groups, as is the case in the current analysis should not affect the power of GLOBETROTTER to identify admixture events. In fact, the program is designed to account for this situation by identifying admixture sources as mixtures of the available donor groups.

The variation in the size of the different donor groups in our analysis is large. This may have an effect on the chromosome painting step of the analysis, as larger groups will provide a greater pool of donor individuals for the recipient individuals to copy from. We try to mitigate against this by using fineSTRUCTURE to initially group together individuals who are statistically indistinguishable from each other, from a genetic point of view. The effect of this step is to standardise individuals into groups that are similarly (un-)related to each other. In this situation, individuals will be equally likely to copy from all individuals from a group, irrespective of group size, because they have, at a given position of genome, the similar haplotype to other individuals in their group.

### S3.4 Running GLOBETROTTER

We used GLOBETROTTER (v2) as per the author's guidelines, initially using the samples and donor copying vectors described above and using all clusters with more than 5 individuals as surrogates. A full

description of the GLOBETROTTER algorithm is provided in Hellenthal et al 2014 [S13]. A brief description of the use of GLOBETROTTER in the current context is provided in the Experimental Procedures section of the main paper.

We performed several further analyses where we removed different groups from being putative admixture surrogates. Whilst our fineSTRUCTURE analysis generated independent clusters of individuals, it is still the case that Eurasian groups are in fact very similar. Including very closely related groups as surrogates is not recommended as there needs to be sufficient differences between the surrogates and target such that the target can be reconstructed as a mixture of surrogates. Put in a different way, if a surrogate copying vector is so similar to the target copying vector that they are very highly correlated then the inference procedure will not work.

## S3.5 Selecting surrogate clusters for use with GLOBETROTTER

When characterising admixture, GLOBETROTTER uses a set of surrogate populations, whose copying vectors are used to describe the composition of the admixing sources. Whilst theoretically any sampled population who has been painted with the same set of donors can be used, we decided to limit the surrogate populations such that two analyses were performed for most clusters:

1. **main analysis** in this analysis, we used all clusters containing at least 5 individuals as surrogate groups. The only exception to this was the finni3 cluster, containing one Norwegian and both of the Finnish individuals included in the analysis, and which we kept in the analysis to include a representative Scandinavian cluster.

2. **masked analyses** using the main analysis surrogate set meant including related groups in the admixture analysis of some of the clusters. For example, with fineSTRUCTURE, we inferred three Sardinian clusters, each of which contained more than five individuals and which were all therefore included as surrogates in the main analysis. In previous work [S13], we showed that masking closely related groups from the analysis can uncover subtle admixture signals.

Practically to achieve this we split the clusters into European and non-European west Eurasian groups and re-ran the inference removing all clusters from the same fineSTRUCTURE clade in the non-European west Eurasian clusters as defined in Figure 1 and Table S3, (i.e. the Caucasus, Anatolia, and Turkey), whilst for European clusters, we re-ran only those clusters where we inferred multiple clusters from the same geographic population. In Tables S4 and S5, the **Analysis** column records whether the main or masked analysis was used and whether the null procedure was additionally used. Note in these analyses, we did not repaint the individuals, but excluded these groups as surrogates. Specifically, we ran the following masked analyses:

1. *slav* masked lithu11 from ukrai48, mask ukrai48 from lithu11

2. *orcad* the four Orcadian clusters were masked as donors from each other

3. Southern Europe

   (a) *italy* we reran the the Italian clusters disallow all other mainland Italian clusters being surrogates

   (b) *sard* we masked the Sardininian groups from each other

   (c) *spani* we masked the other Spanish cluster from both of these clusters

4. *turk* we re-ran GLOBETROTTER for each Turkish cluster disallowing all other Turkish clusters from being surrogates

5. *anat* we re-ran GLOBETROTTER for each Armenian/Iran cluster disallowing all other Anatolian clusters from being surrogates

6. *wca* we re-ran GLOBETROTTER for each West Central Asia cluster disallowing all other North Caucasus clusters from being surrogates

7. *ecauc* we re-ran GLOBETROTTER for each East Caucasus cluster disallowing all other East Caucasus clusters from being surrogates

8. *wcauc* we re-ran GLOBETROTTER for each West Caucasus cluster disallowing all other West Caucasus clusters from being surrogates

# S4 Results of GLOBETROTTER admixture analysis on 82 Eurasian Groups

## S4.1 Obtaining an admixture result with GLOBETROTTER

For completeness, we include the results of all GLOBETROTTER analyses performed on the dataset as well as the final set of results that we use to inform the Results and Discussion in the main paper. Further information and a version of the program available to download can be found at www.paintmychromosomes.com. In each run of the program, GLOBETROTTER produces one of the following characterisations of admixture:

1. *no admixture* (NA): we generate an admixture $P$ value by running 100 date bootstraps using the null procedure and compute the number of these bootstraps where the inferred data is $> 400$ and $< 1$. If the $P$ value $\geq 0.01$, we infer no admixture.
2. *uncertain* (U): admixture is detected but difficult to describe (combined fit quality for two events "fit.quality.2events" $< 0.985$, or $R^2$ coefficient of determination of the coancestry curves $< 0.2$)
3. *one-date* (1D): a single date of admixture between two sources (combined fit quality for two events $\geq 0.985$; two-date score "maxScore.2events" < 0.35; fit-quality for a single event "fit.quality.1event" $\geq 0.975$)
4. *one-date-multiway* (1MW):a single date of admixture between more than two sources (combined fit quality for two events $\geq 0.985$; two-date score $< 0.35$; fit-quality for a single event $< 0.975$)
5. *multiple-dates* (2D): two (or more) distinct dates of admixture between two or more sources (combined fit quality for two events $\geq 0.985$; two-date score $\geq 0.35$)

Additionally, for all events where we infer multiple dates, we checked the results of 100 two date bootstraps. In such cases, if the lower bound of the more recent admixture date bootstrap interval was $\leq 3$ generations, we switched the final result to either 1D, if the fit quality for the first event $< 0.975$ or 1MW if $\geq 0.975$.

## S4.2 Summary of the GLOBETROTTER results tables

To identify our final results used in the paper, we chose the masked analysis in preference to the main analysis, unless the event inferred in the masked analysis was *uncertain*. In such cases we instead used the result from the main analysis. The final results are shown in Table S4. We also provide the full output from all main and masked runs as well as associated runs in Table S5. We also report the results of running GLOBETROTTER with the null procedure for these analyses. In general, the results of these two analyses were qualitatively similar: the effect of removing closely related clusters from the analysis tends to result in the next most similar cluster being chosen as the best matching source.

As noted above, in some cases where the initial GLOBETROTTER results was inferred to be two dates it was necessary to alter the admixture event inference based on the date bootstraps. In these cases the **res** column contains both the original result (in parentheses) as well as the final result used in the paper. In such cases we include this information in the final table for reference.

In general, when reading the GLOBETROTTER results tables one should identify the admixture result, from the **res** column and then identify the set of columns header with this value to find the detailed admixture characteriations

## S4.3 Inferring admixture source copying vectors

GLOBETROTTER estimates the proportion that each source contributes to an admixture event $\alpha$ together with the proportions that all donor copying vectors contribute to each source, which we term $\beta$. For each admixing source, the $\beta$s sum to one. We can thus recreate the genomic identity for each of the the admixing sources on either side of an event in the form of a copying vector. To do so, for each group involved in the mixture of a source, we take their original copying vector and multiply this by $\beta$ to generate an 'inferred' copying vector for each source involved in an event. Therefore, in addition to the inference of admixing proportions and dates, we are also able to gain insight into the genetic identity of the sources of admixture.

We can use these source copying vectors in a number of ways. We can compare them to the set of donor (in this case, the fineSTRUCTURE cluster) copying vectors to identify the closest matching contemporary group to the admixture source, which we do to find the "best-matching" donor for each admixture source in Tables S4 and S5 and the figures in the main paper. We can also compare them both to each other and to different sets of copying vectors to provide further understanding on the variation present in the dataset. This approach appeals because it allows us to characterise the genetic profile of the sources of admixture back in time, when they are unlikely to be most similar to a single contemporary group, so viewing them as mixtures is a more appropriate method of viewing these events that happened in the past.

In practice, to compute an inferred source copying vector, we use the $\beta$s and our original copying vectors (e.g. $f^{donor}$) and generate the genetic profile of the admixture source simply as the product of the $\beta$ coefficients and these $f$s. For example, if an admixture source, $f^{source}$, is inferred to be made up of 50% East Asia ($\beta_{EastAsia}$=0.5) and 50% Mongolia ($\beta_{Mongolia}$=0.5), then:

$$f^{source} = \beta_{EastAsia} \times f^{EastAsia} + \beta_{Mongolia} \times f^{Mongolia} = 0.5 \times f^{EastAsia} + 0.5 \times f^{Mongolia}$$

More formally, we have mixture coefficients $\beta_1, \beta_2, ..., \beta_K$ corresponding to the mixing coefficients for populations $1 \leq l \leq K$, where $\beta_l > 0$, so:

$$f = \sum_{l=1}^{K} \beta_l f^l$$

For each event we can generate a major and minor source copying vector in this way, which, as before we normalise to sum to 1, and which is of exactly the same form as copying vectors of both present-day individuals and present-day clusters. This also allows us to (a) project the source genetic profiles onto PCA space computed from the present-day individuals, an aspect which we utilise in Figure 4, and (b) estimate the variation present within groups of copying vectors both before and after admixture.

## S4.4 Inferring admixture source copying vectors prior to admixture

We show above the procedure for using GLOBETROTTER to infer a genetic profile for the sources of admixture. In the current setting we are interested in characterising the genetic profile of our Eurasian groups prior to admixture. There are two ways to generate such profiles. The first is to generate the major admixture source exactly as described above:

$$f^{PRE} = f^{MAJOR} = \sum_{l=1}^{K} \beta_l^{MAJOR} f^l$$

where $\beta_l^{MAJOR}$ are the mixture coefficients for the $l$ donor groups involved in the major admixture source

mixture inferred by GLOBETROTTER. However, because we model admixture between a major and minor source mixing at proportion $\alpha$, an alternative estimate of the pre-admixture source copying vector can be made by "removing" the minor source of admixture from the original (post-admixture) fineSTRUCTURE cluster copying vector:

$$f^{PRE} = f^{POST} - (\alpha \times f^{MINOR})$$

The estimate of $f^{POST}$, which is in practice the cluster copying vector inferred from the painting, implicitly includes a drift component. As such, our estimates of the pre-admixture source group copying vectors inferred in these two ways should be similar, but are unlikely to be identical. This is because, in the second case, we incorporate an error term into our estimate. That is, our estimate of the pre-admixture source copying vector is in fact:

$$f^{PRE} = f^{POST} - (\alpha \times f^{MINOR}) = f^{MAJOR} + \epsilon$$

The main two sources of the error ($\epsilon$) here are likely to be imperfect characterisation of the admixture process and genetic drift specific to the cluster being considered ($f^{POST}$), which is unlikely to be well identified in the GLOBETROTTER admixture source inference. At present we are unable to fully model this error, but we are able to account for it when we compare copying vectors to each other.

## S4.5 Estimating diversity in West Eurasia before and after admixture

Although, as outlined above, we identified two alternative approaches for estimating the pre-admixture genetic profile of a cluster, we are still interested in attempting to quantify the change in diversity before and after admixture. We can therefore perform an analysis where we compare the two different pre-admixture sources to separate estimates of the current post-admixture West Eurasian diversity. For the 64 clusters where we inferred admixture we computed the total variation distance (TVD; see below) [S21] for all pairs of copying vectors in the the following four groups (for clusters with multiple sources or dates, we only used the sources from the first event):

1. pre-admixture 1: $f^{MAJOR}$ – the major sources of admixture
2. pre-admixture 2: $f^{POST} - \alpha f^{MINOR}$ – the minor source of admixture "removed" from the cluster copying vector
3. post-admixture 1: $(1-\alpha) \times f^{MAJOR} + \alpha \times f^{MINOR}$ – the admixed group inferred by GLOBETROTTER
4. post-admixture 2: $f^{POST}$ – the fineSTRUCTURE cluster copying vector

In order to disregard the additional error (drift) components that will not be characterised well by GLOBETROTTER, we compared (a) the average TVD (pre-admixture1) with TVD (post-admixture1) and (b) TVD (pre-admixture2) with TVD (post-admixture2). We report (a) in Figure 4 and (a) and (b) in Figure S4. In general we see that variation is greater when we consider the fineSTRUCTURE clusters (e.g. Figure S4I and Figure S4J), but that in general there are no large differences between the TVD estimated across copying vectors within the four groups (Figure S4B), with the caveat that there is perhaps a suggestion that the TVD amongst the major sources of admixture is less than in the other groups. Figure S4K shows that when we compare, for each of the 64 groups where we infer admixture, the TVD between the two methods of inferring "pre-admixture" sources (x-axis) and the two methods for inferring "post-admixture" groups, the variation is highly correlated. This suggests that the differences between the two estimation methods are likely to be because of the same error.

### S4.5.1 Computing TVD

We used Total Variation Distance (TVD) to compare copying vectors [S21]. As the copying vectors are discrete probability distributions over the same set of donors, TVD is a natural metric for quantifying the difference between them. For a given pair of groups $A$ and $B$ with copyinig vectors describing the copying from $i$ donors, $a_i$ and $b_i$ we can estimate TVD with the following equation:

$$TVD = 0.5 \times \sum_{i=1}^{n}(|a_i - b_i|)$$

## S4.6 The landscape of admixture in West Eurasia

To generate panel (A) Figure 4 in the main text, we took the all individuals from each geographic sampling location and assigned ancestry to them based on the results of GLOBETROTTER. The location of the points are shown in Figure S4A. In locations containing individuals from multiple clusters, we averaged these proportions across all individuals within a location to arrive at the final estimates of ancestry for a given location. To generate panel (C) we show arrows from admixture donor groups to recipients, where the admixture donors are sources where the best-matching coying vector is a West Eurasian group. Panel (D) is an analysis that plots the copying vector of each of these West Eurasian sources on a PCA of contemporary West Eurasians.

# S5 Additional discussion of results

In the following section we first provide a brief, direct, comparison of admixture events that we infer in Sardinia and the Balkans with previous studies using different methods and datasets. We then discuss the inferred admixture events from our study in more detail. Except in a couple of cases, we restrict the following discussion to the 57 recipient clusters containing at least four individuals.

## S5.1 Comparison to previous results

1. Moorjani et al [S19], who use a method based on allele frequency comparisons, and not haplotypes (ROLLOFF), found evidence for sub-Saharan African admixture in Sardinia 71$\pm$28 generations ago, at a proportion of 3%. These are the same Sardinians included in our analysis. In the largest Sardinian (sardi13) cluster in our analysis we infer West African admixture 66 (53-82) generations ago at a proportion of 2%.

2. Ralph and Coop [S9] using a method that infers tracts of ancestry by inferring Identity by Descent (IBD) along chromosomes, showed evidence that individuals from the Balkans have a high number of shared ancestors, with the length of these shared IBD tracts consistent with common ancestry from the Migration Period. We also find admixture events dating to this period in groups from the Balkans, for example in groups from Hungary (hunga23), Bulgaria (bulga46) and Croatia (croat18), in which we infer north east European ancestry flowing into more southerly regions. Ralph and Coop use a different dataset (POPRES) and different methods to produce qualitatively similar results to us.

## S5.2 Continuous low level African admixture in the Mediterranean and Anatolia

We infer West African admixture across broad date ranges, but at low admixture proportions (admixture $\alpha < 0.07$; Figs. 2 and S3) in several Mediterranean groups, consistent with a long term movement between sub-Saharan Africa and southern Europe [S22,S23]. Specific West African admixture dating to the Arabic conquest of the Mediterranean [S24] is seen in Spanish (spani27: 1042 (740-1201CE)), Southern Italian and Sicilian (sicil30: 1105 (882-1250CE)), and Basque (basqu24: 886 (283-1162CE)) clusters. Earlier African admixture at low admixture proportion is inferred in the Cypriots (cypri12: 427(107-734CE)), and a Sardinian cluster (sardi13: 36 (458BCE-430CE); $\alpha = 0.02$). This latter event is consistent with the occurrence of A3b2-M13 (0.6%) and E1a-M44 (0.4%) African Y chromosome lineages in Sardinia [S25]. and the dating is more compatible with documented exchanges between the island and *Mauretania Cesariensis* in Roman times (2$^{nd}$ century BCE to 2$^{nd}$ century CE) than later displacements of northern-African males to Sardinia at the time of the Vandals rule (5$^{th}$ century CE) [S24].

Two Iranic clusters show evidence of African admixture, from West Africa in a Kurdish cluster (kurd5: 872CE (312-1069CE)), and from East Africa in an Iranian cluster (irani5: 1412CE (1054CE-1526CE)). The low admixture proportion ($\alpha = 0.01$) in the Kurdish cluster suggests a very subtle event, the dates of which roughly align with the Arabic conquest of the Mediterranean and the increased movement of sub-Saharan African slaves in the region [S13]. Admixture is more recent in the Iranian cluster, and similarly at a low admixture proportion ($\alpha = 0.03$), but the distinct eastern origin of the African ancestry in this group suggests a different route of African admixture into the region, across the Indian Ocean and Arabia. We observe subtly different signatures of admixture in each of the three Sardinian clusters, as already discussed from West Africa, but also from North Africa, specifically Tunisian and Moroccan sources (sardi9: 356 (507BCE-756CE); $\alpha = 0.09$), and also from the Levant source at a

proportion of $\alpha = 0.07$ (sardi6: 449 (11BCE-755CE)). The dates for these events overlap, consistent with African ancestry originating from a number of different sources. Previous admixture analysis of these individuals found evidence for a small amount of sub-Saharan admixture [S19,S26] around 40 generations ago, which we corroborate here, with the additional suggestion of multiple complex African ancestral histories within individuals from the island.

## S5.3 A key role for the Levant in the genetic history of the Mediterranean

Early admixture involving source groups most similar to contemporary populations from in and around the Levant (which we define as the World Region containing individuals from Syria, Palestine, Lebanon, Jordan, Saudi, Yemen and Egypt) is seen at high proportions in several clusters from Italy dating to the first half of the first millennium CE, from Southern Italy (itali8: 295CE (72BCE-604CE); $\alpha = 0.34$), Tuscany (tsi23: 400CE(30BCE-686); $\alpha = 0.29$), and Sardinia, as well as in a large cluster from Armenia at an early date (armen27: 363BCE(1085BCE-383CE)). Traces of Phoenician ancestry (1200-300BCE) have been observed using uni-parental markers from populations around the Mediterranean [S27] which, based on the dates, is unlikely to be the source of ancestry we observe here. Instead these events loosely coincide with the formation of the pan-Mediterranean Roman Empire [S24], which may also have allowed increased gene flow from east to west Mediterranean. A significant amount of ancestry ($\alpha = 0.36$) in a large Armenian cluster (armen27) is the result of a complex ancient event involving multiple admixture sources from the Caucasus, Turkey, and the Levant. Armenia was at various times across this period part of Roman, Christian, Parthian and Arabic empires. Whilst it is impossible to identify a particular event that caused the admixture that we see, the proportion, timing and identity of the source groups in this event are consistent with Armenia's geographic and political position at a junction between Europe and west Asia. Similarly, we also infer a complex event involving the Levant, Central Asia and Turkish sources in an Iranian cluster (irani10), although dates for this event are more recent (1221CE (1063-1330CE)) and therefore more consistent with influx on ancestry from the Near East during and after the Mongolian expansions into western Eurasia. We infer more recent Levant admixture in the French (frenc24: 728(424-1011CE)) and in a complex multiway event in a Spanish cluster (spani9: 668 (286-876CE)). The dates and sources of admixture in these cases are consistent with movements of Middle Eastern and North African individuals during the Islamic Conquest of Spain [S24], and suggest a legacy of this key moment in southern European history in the genomes of French as well as Spanish populations.

## S5.4 Multiple waves of admixture from east Asia

The Caucasus contain many linguistically diverse but genetically homogeneous populations [S28]. The historical influence of Asia is particularly clear in the Caucasus and Anatolia, where most events involve Mongolian sources and occur after 1000CE (Figs. 2 and S3, Table S4). Two clusters from Turkey (turki34, turki17) show clear evidence of admixture from Mongolia, which is also present in several groups from the steppe east of the Caucasus, in clusters containing individuals from Tajikistan (tajik11: 1295(1152-1374CE)) and Turkmenistan (turkm11: 1266(1159-1323CE); turkm5: 1369 (1226-1508CE)). Dates for these events centre around 1250CE, suggesting further evidence of the large-scale impact of Chinnghid Mongolian nomads in Western Eurasia [S13,S29] in a wide set of populations. The more recent events involving Mongolian sources in Eastern and Northern Caucasus clusters containing Nogay, Kumyk, Lezgin, and Tabasaran individuals centre around 1400-1500CE which may related to the post-Mongol Timurud dynasty which ruled Central Asia and the Caucaus between 1360 and 1425CE [S30].

Among the Caucasus clusters where we do not specifically infer Mongolian admixture, we find evidence of events, often involving multiple admixture sources including Central Asian (Uzbek and Hazara) sources, in clusters containing Turkish (turki7: 1015 (541-1309CE)), Balkasian (balka5: 1177 (876-1389CE)), and Iranian (irani10: 1221 (1063-1330CE)) individuals. The dates for these events, which peak around 750 years ago, together with the non-Mongolian source groups, suggest a role for secondary movement into this area at the time of the Mongolian expansion, but from Asian groups with distinct non-Mongolian ancestry.

A cluster of two individuals from Romania (roman2: 990 (741-1245CE)) have clear evidence of an admixture event involving a source most similar to India. Romany gypsies have been shown to have ancestry from the India subcontinent, which has been dated to an event between 780 and 900 years ago [S31,S32]. Our date of 800 years ago, or 1200CE broadly agrees with these inferences. The identity of these two individuals as Romani is not possible from the data associated with the samples, but if confirmed, shows that in certain settings GLOBETROTTER can produce accurate historical inference even with limited sample sizes.

Among the northern Europeans, the Finnish (finni3) show evidence of an admixture event involving a minority source most similar to contemporary North Siberians (469CE (213BCE-1011CE)). Finns are thought to have originated from the northward migration, and subsequent contact, between Central Europeans and indigenous Scandinavian hunter-gatherers closely related to the Saami [S33]. The Saami are closely related to the individuals that make up the North Siberian world region, and whilst our confidence in this admixture date is low because of the small size of the cluster, the event we see is likely to represent this key period in Finnish history. Within our dataset, only the Finnish, Hungarians and Mordovians speak Finno-Ugric languages, the latter of which we group into two clusters (mordo13: 792 (564-975CE); mordo2: 558 (179-843CE)) and, together with the Russians (russi25: 913(754-1007CE)) and Chuvash (chuva16: 829 (627-940CE)) populations, infer admixture at approximately the same time (500-900CE) involving Mongolian, Central European, and Finnish donors. In a recent analysis that reconstructed the ancestry of Eurasia on the basis of ancient DNA [S34], the ancestry of these groups could not be explained without a putative stream of recent Asian admixture, a scenario which we confirm in our analysis. As such, the Asian admixture in these groups is unlikely to be associated with the Mongolian expansion described above and may instead be related to earlier Turkic movements, involving the Huns and Avars [S29], but separate to the event inferred in the Finnish.

## S5.5 Admixture within Europe: the Medieval Migration Period

A separate set of events involves admixture between groups within West Eurasia. As we previously reported [S13], the formation of the Slavic people at around 1000CE had a significant impact on the populations of northern and eastern Europe, a result that is supported by a related but different analysis [S9]. We infer events involving a “Slavic” source (represented here by a cluster of Lithuanians; lithu11) across all Balkan groups in the analysis (Greece, Bulgaria, Romania, Croatia, and Hungary) as well as in a large cluster of Germanic origin (germa36) and a composite cluster of eastern European individuals (ukrai48). Dates for these events mostly overlap, although are older in Croatia and Greece, and appear to concentrate on the end of the first millenium CE (Figure 3), a time known as the European Migration Period, or *Völkerwanderung* [S35]. We additionally infer events during this period in the Spanish (spani9: 668 (286-876CE)), involving Basque- and northern Italian-like sources, in the British (ceu71: 858 (467-1224CE) involving German-, Central and Southern Italian-, and Norwegian-like sources , in the Orcadians (orcad5: 1241 (889-1412CE); orcad6: 708 (94BCE-1399)) involving Norwegian-, Southern Italian-, and Armenian-like sources, in the Norwegians (norwe17: 351 (262BCE-893CE)) involving Mordovian- and British- like sources, in the northern Italians (itali13: 677 (362-989CE)) involving Cypriot- and French-like sources, and in a large cluster of Tuscans (tsi70: 241 (16BCE-417CE)) involv-

ing Cypriot- and British-like sources. Interestingly, these groups contain individuals that are largely from north-western and central European regions with historically attested influences from different groups during the *Völkerwanderung* [S35], suggesting that this period had a further visible effect on the contemporary populations across Northern and Central Europe.

## Supplemental References


[S1] Price AL, Patterson NJ, Plenge RM, Weinblatt ME, Shadick NA, et al. (2006) Principal components analysis corrects for stratification in genome-wide association studies. Nat Genet 38: 904–909.

[S2] Delaneau O, Marchini J, Zagury JF (2012) A linear complexity phasing method for thousands of genomes. Nature Methods 9: 179–181.

[S3] Li N, Stephens M (2003) Modeling linkage disequilibrium and identifying recombination hotspots using single-nucleotide polymorphism data. Genetics 165: 2213–2233.

[S4] Alexander DH, Novembre J, Lange K (2009) Fast model-based estimation of ancestry in unrelated individuals. Genome Res 19: 1655 –1664.

[S5] Lawson DJ (2015) Populations in Statistical Genetic Modelling and Inference. In: Kreager P, Winney B, Ulijaszek S, Capelli C, editors, Population in the Human Sciences: Concepts, Models, Evidence, Also available as: eBook.

[S6] Cavalli-Sforza LL, Menozzi P, Piazza A (1994) The history and geography of human genes. Princeton University Press.

[S7] Lawson DJ, Hellenthal G, Myers S, Falush D (2012) Inference of Population Structure using Dense Haplotype Data. PLoS Genet 8: e1002453.

[S8] Consortium TIH (2010) Integrating common and rare genetic variation in diverse human populations. Nature 467: 52–58.

[S9] Ralph P, Coop G (2013) The Geography of Recent Genetic Ancestry across Europe. PLoS Biol 11: e1001555.

[S10] Novembre J, Johnson T, Bryc K, Kutalik Z, Boyko A, et al. (2008) Genes mirror geography within Europe. Nature 456: 98–101.

[S11] Lao O, Lu TT, Nothnagel M, Junge O, Freitag-Wolf S, et al. (2008) Correlation between Genetic and Geographic Structure in Europe. Current Biology 18: 1241–1248.

[S12] Jakobsson M, Rosenberg NA (2007) CLUMPP: a cluster matching and permutation program for dealing with label switching and multimodality in analysis of population structure. Bioinformatics 23: 1801–1806.

[S13] Hellenthal G, Busby GBJ, Band G, Wilson JF, Capelli C, et al. (2014) A Genetic Atlas of Human Admixture History. Science 343: 747–751.

[S14] Chen GK, Marjoram P, Wall JD (2009) Fast and flexible simulation of DNA sequence data. Genome Res 19: 136–142.

[S15] Gronau I, Hubisz MJ, Gulko B, Danko CG, Siepel A (2011) Bayesian inference of ancient human demography from individual genome sequences. Nat Genet 43: 1031–1034.

[S16] Li H, Durbin R (2011) Inference of human population history from individual whole-genome sequences. Nature 475: 493–496.

[S17] Gutenkunst RN, Hernandez RD, Williamson SH, Bustamante CD (2009) Inferring the Joint Demographic History of Multiple Populations from Multidimensional SNP Frequency Data. PLoS Genet 5: e1000695.

[S18] Keinan A, Mullikin JC, Patterson N, Reich D (2007) Measurement of the human allele frequency spectrum demonstrates greater genetic drift in East Asians than in Europeans. Nat Genet 39: 1251–1255.

[S19] Moorjani P, Patterson N, Hirschhorn JN, Keinan A, Hao L, et al. (2011) The History of African Gene Flow into Southern Europeans, Levantines, and Jews. PLoS Genet 7: e1001373.

[S20] Henn BM, Botigué LR, Gravel S, Wang W, Brisbin A, et al. (2012) Genomic Ancestry of North Africans Supports Back-to-Africa Migrations. PLoS Genet 8: e1002397.

[S21] Leslie S, Winney B, Hellenthal G, Davison D, Boumertit A, et al. (2015) The fine-scale genetic structure of the British population. Nature 519: 309–314.

[S22] Auton A, Bryc K, Boyko AR, Lohmueller KE, Novembre J, et al. (2009) Global distribution of genomic diversity underscores rich complex history of continental human populations. Genome Research 19: 795–803.

[S23] Botigué LR, Henn BM, Gravel S, Maples BK, Gignoux CR, et al. (2013) Gene flow from North Africa contributes to differential human genetic diversity in southern Europe. PNAS 110: 11791–11796.

[S24] Roberts J (2007) The New Penguin History of the World. London, UK: Penguin Books, 5th edition.

[S25] Francalacci P, Morelli L, Angius A, Berutti R, Reinier F, et al. (2013) Low-Pass DNA Sequencing of 1200 Sardinians Reconstructs European Y-Chromosome Phylogeny. Science 341: 565–569.

[S26] Loh PR, Lipson M, Patterson N, Moorjani P, Pickrell JK, et al. (2013) Inferring Admixture Histories of Human Populations Using Linkage Disequilibrium. Genetics 193: 1233–1254.

[S27] Zalloua P, Platt D, El Sibai M, Khalife J, Makhoul N, et al. (2008) Identifying Genetic Traces of Historical Expansions: Phoenician Footprints in the Mediterranean. American Journal of Human Genetics 83: 633–642.

[S28] Yunusbayev B, Metspalu M, Järve M, Kutuev I, Rootsi S, et al. (2011) The Caucasus as an asymmetric semipermeable barrier to ancient human migrations. Molecular Biology and Evolution .

[S29] Atwood C P (2004) Encyclopedia of Mongolia and the Mongol Empire. New York, USA: Facts on File, Inc.

[S30] Beckwith CI (2006) Empires of the Silk Road: A History of Central Eurasia from the Bronze Age to the Present. Princeton, US: Princeton University Press.

[S31] Mendizabal I, Lao O, Marigorta U, Wollstein A, Gusmão L, et al. (2012) Reconstructing the Population History of European Romani from Genome-wide Data. Current Biology 22: 2342–2349.

[S32] Moorjani P, Patterson N, Loh PR, Lipson M, Kisfali P, et al. (2013) Reconstructing Roma History from Genome-Wide Data. PLoS ONE 8: e58633.

[S33] Huyghe JR, Fransen E, Hannula S, Van Laer L, Van Eyken E, et al. (2011) A genome-wide analysis of population structure in the Finnish Saami with implications for genetic association studies. Eur J Hum Genet 19: 347–352.

[S34] Lazaridis I, Patterson N, Mittnik A, Renaud G, Mallick S, et al. (2014) Ancient human genomes suggest three ancestral populations for present-day Europeans. Nature 513: 409–413.

[S35] Heather P (2009) Empires and Barbarians: migration, development and the birth of Europe. London, UK: Macmillan.
