# Supplementary material for: The Role of Recent Admixture in Forming the Contemporary West Eurasian Genomic Landscape
Source: Curr Biol. 2015 Oct 5;25(19):2518–26. doi: 10.1016/j.cub.2015.08.007 (PMC4714572; doi:10.1016/j.cub.2015.08.007)
Supplement: Document S2. Article plus Supplemental Information [file mmc3.pdf]

Report

Current Biology

The Role of Recent Admixture in Forming the Contemporary West Eurasian Genomic Landscape

Highlights

- Recent admixture events involved outside groups at the edges of West Eurasia
- Admixture within Europe tended to fall within the European Migration Period
- West Eurasian genetic structure today is likely to have been maintained by admixture

Authors

George B.J. Busby, Garrett Hellenthal, Francesco Montinaro, ..., James F. Wilson, Simon Myers, Cristian Capelli

Correspondence

george@well.ox.ac.uk (G.B.J.B.),
cristian.capelli@zoo.ox.ac.uk (C.C.)

In Brief

Using cutting edge statistical machinery, Busby et al. show that recent admixture is ubiquitous across West Eurasia, with the majority of populations showing evidence of population mixing. Dating of these admixture events demonstrates that the Medieval Migration Period was a key period in establishing the current West Eurasian genetic landscape.

Accession Numbers

GSE71603

Busby et al., 2015, Current Biology *25*, 2518–2526
October 5, 2015 ©2015 Elsevier Ltd All rights reserved
http://dx.doi.org/10.1016/j.cub.2015.08.007

Current Biology
Report

The Role of Recent Admixture in Forming the Contemporary West Eurasian Genomic Landscape

George B.J. Busby,[1,2,*] Garrett Hellenthal,[3] Francesco Montinaro,[1] Sergio Tofanelli,[4] Kazima Bulayeva,[5] Igor Rudan,[6] Tatijana Zemunik,[7] Caroline Hayward,[8] Draga Toncheva,[9] Sena Karachanak-Yankova,[9] Desislava Nesheva,[9] Paolo Anagnostou,[10,11] Francesco Cali,[12] Francesca Brisighelli,[1,13] Valentino Romano,[14] Gerard Lefranc,[15] Catherine Buresi,[15] Jemni Ben Chibani,[16] Amel Haj-Khelil,[16] Sabri Denden,[16] Rafal Ploski,[17] Pawel Krajewski,[18] Tor Hervig,[19] Torolf Moen,[20] Rene J. Herrera,[21] James F. Wilson,[6,8] Simon Myers,[2,22] and Cristian Capelli[1,*]

[1]Department of Zoology, University of Oxford, South Parks Road, Oxford OX1 3PS, UK
[2]Wellcome Trust Centre for Human Genetics, University of Oxford, Roosevelt Drive, Oxford OX3 7BN, UK
[3]UCL Genetics Institute, University College London, Gower Street, London WC1E 6BT, UK
[4]Department of Biology, Università di Pisa, Via Ghini 13, 56126 Pisa, Italy
[5]N.I.Vavilov Institute of General Genetics, 3 Gubkin Street, Moscow 119991, Russia
[6]Centre for Global Health Research, Usher Institute of Population Heath Sciences and Informatics, University of Edinburgh, Teviot Place, Edinburgh EH8 9AG, UK
[7]Department of Medical Biology, School of Medicine Split, Soltanska 2, Split 21000, Croatia
[8]MRC Human Genetics Unit, Institute of Genetics and Molecular Medicine (IGMM), University of Edinburgh, Western General Hospital, Crewe Road, Edinburgh EH4 2XU, UK
[9]Department of Medical Genetics, National Human Genome Center, Medical University Sofia, Sofia 1431, Bulgaria
[10]Department of Environmental Biology, Università La Sapienza, Roma 00185, Italy
[11]Istituto Italiano di Antropologia, Roma 00185, Italy
[12]Laboratorio di Genetica Molecolare, IRCCS Associazione Oasi Maria SS, Troina 94018, Italy
[13]Forensic Genetics Laboratory, Institute of Legal Medicine, Università Cattolica del Sacro Cuore, Rome 00168, Italy
[14]Dipartimento di Fisica e Chimica, Università di Palermo, Palermo 90128, Italy
[15]Institute of Human Genetics, CNRS UPR 1142, and Montpellier University, Place Eugene Bataillon, 34095 Montpellier Cedex 5, France
[16]Laboratory of Biochemistry and Molecular Biology, Faculty of Pharmacy, 1 Avenue Avicenne, 5019 Monastir, Tunisia
[17]Department of Medical Genetics, Warsaw Medical University, 3c Pawinskiego Street, Warsaw 02-106, Poland
[18]Department of Forensic Medicine, Warsaw Medical University, 1 Oczki Street, Warsaw 02-007, Poland
[19]Department of Clinical Science, University of Bergen, Bergen 5021, Norway
[20]NTNU, Trondheim 7491, Norway
[21]Department of Human and Molecular Genetics, Florida International University, University Park, Miami, FL 33174, USA
[22]Department of Statistics, University of Oxford, South Parks Road, Oxford OX1 3TG, UK
*Correspondence: george@well.ox.ac.uk (G.B.J.B.), cristian.capelli@zoo.ox.ac.uk (C.C.)
http://dx.doi.org/10.1016/j.cub.2015.08.007

SUMMARY

Over the past few years, studies of DNA isolated from human fossils and archaeological remains have generated considerable novel insight into the history of our species. Several landmark papers have described the genomes of ancient humans across West Eurasia, demonstrating the presence of large-scale, dynamic population movements over the last 10,000 years, such that ancestry across present-day populations is likely to be a mixture of several ancient groups [1–7]. While these efforts are bringing the details of West Eurasian prehistory into increasing focus, studies aimed at understanding the processes behind the generation of the current West Eurasian genetic landscape have been limited by the number of populations sampled or have been either too regional or global in their outlook [8–11]. Here, using recently described haplotype-based techniques [11], we present the results of a systematic survey of recent admixture history across Western Eurasia and show that admixture is a universal property across almost all groups. Admixture in all regions except North Western Europe involved the influx of genetic material from outside of West Eurasia, which we date to specific time periods. Within Northern, Western, and Central Europe, admixture tended to occur between local groups during the period 300 to 1200 CE. Comparisons of the genetic profiles of West Eurasians before and after admixture show that population movements within the last 1,500 years are likely to have maintained differentiation among groups. Our analysis provides a timeline of the gene flow events that have generated the contemporary genetic landscape of West Eurasia.

RESULTS AND DISCUSSION

The Genetic Structure of West Eurasia

Previous analyses of population structure have shown that despite high genetic similarity, European genetic structure is clinal and therefore heavily influenced by geography [12, 13]. But Eurasian populations are also genetically heterogeneous;

 ©2015 Elsevier Ltd All rights reserved

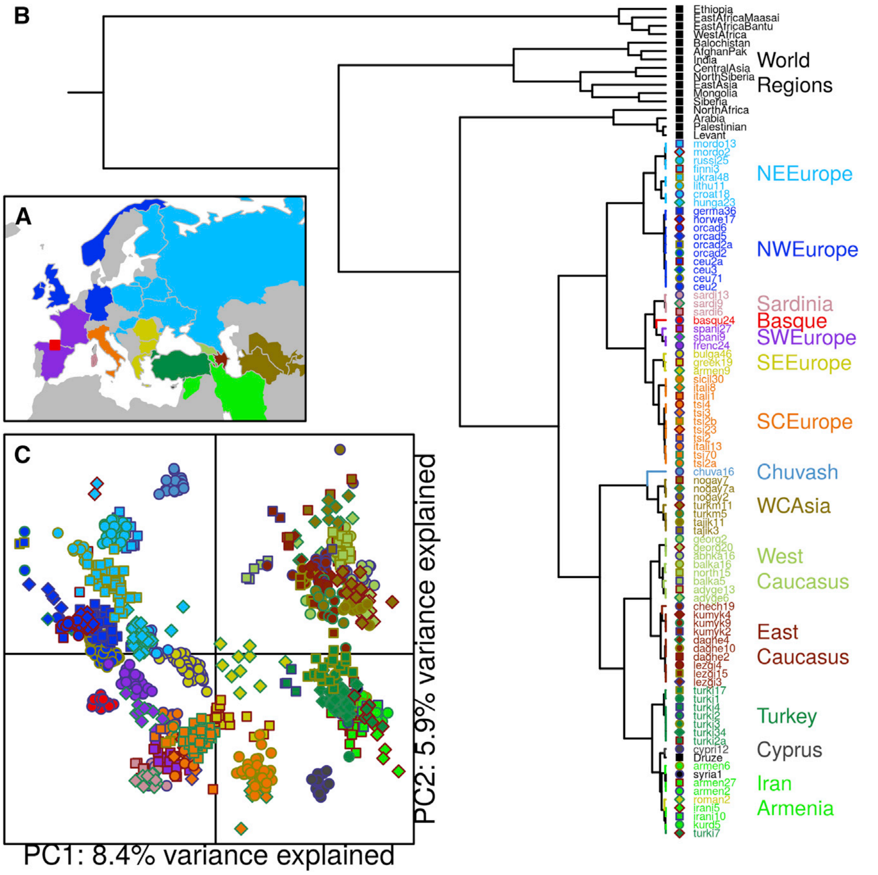

Figure 1. Structure and Relationships in West Eurasia

(A) Map of West Eurasia showing the colors assigned to each major West Eurasian region. A full list of geographical populations used in the analysis is shown in Table S1.

(B) The fineSTRUCTURE tree of the dataset showing the clusters used in the analysis. Cluster labels contain an alphabetical prefix relating to the geographical population label of the majority of individuals within a cluster. The numerical suffix describes the total number of individuals within a cluster. A full description of the identity of the individuals in each cluster is shown in Table S3.

(C) Principal-component analysis (PCA) of the chunkcounts coancestry matrix used in fineSTRUCTURE. Each point is an individual and is labeled according to the fineSTRUCTURE cluster that it groups into as in (B).

in some countries (e.g., Sardinia) multiple genetic clusters of individuals can be inferred from genetic data, whereas in others (e.g., Basques), individuals are more similar [14]. To gain insight into the historical processes behind population genetic patterns in West Eurasia, we compiled a dataset of 1,235 phased West Eurasian genomes from 63 populations combined with 957 individuals from an additional 87 worldwide populations (Table S1) [11, 15–17]. We accounted for potential substructure within groups with the same geographic population label by performing an analysis of population structure using the fineSTRUCTURE [14] genetic clustering algorithm, which identifies groups of individuals who are statistically indistinguishable from each other from a genetic point of view (Figure 1 and Supplemental Information). This approach gains power over traditional methods of defining population structure like ADMIXTURE [18] or principal-component analysis (PCA) [19] by explicitly modeling the correlation structure among nearby SNPs due to linkage disequilibrium (LD) [20]. Moreover, because this clustering brings together individuals on the basis of shared ancestry, there should be a reduction in the noise in the admixture inference process that can come from having individuals with different ancestries within the same geographic population [21]. The algorithm additionally reconstructs the hierarchical relationships between the clusters, in the form of a tree, that allowed us to redefine West Eurasia as the monophyletic clade of 82 fineSTRUCTURE clusters containing 1,000 individuals (Figures 1 and S1B), which incorporates all of mainland Europe, Sardinia, Sicily, Cyprus, western Russia, the Caucasus, Turkey, and Iran, and some individuals from Tajikistan and Turkmenistan. Similarly, we defined 18 World Regions containing sets of related clusters within different broad geographic regions of the world. In general, within West Eurasia, we see that often the majority of individuals with the same geographic population label fall into the same genetic cluster (for example, the Basques, Greeks, and Mordovians), although sometimes individuals from larger geographic populations, such as Spain and HAPMAP CEU individuals (Northern and Western Europeans), are split into several clusters, which likely represents true substructure. In other cases, such substructure is not evident, and individuals from multiple populations are merged (for example Poland, Ukraine, Belorussia), suggesting that geographic population labels do not always describe genetic similarity, further motivating our genetic clustering approach (see also Figure S1A).

We investigated the effect of admixture—the process of mixing of haplotypes between genetically differentiated ancestral groups—in each of these clusters using GLOBETROTTER [11, 20]. First, we painted each recipient individual's chromosomes such that they were represented as mosaics of chunks of different ancestry from a set of donor groups that included all 18 World Regions together with other clusters from within West Eurasia. We then used summaries of the amount of genome-wide donor ancestry from these mosaics, together with information on the lengths and distributions of specific ancestral chunks, to infer whether admixture is likely to have occurred in a recipient group and to characterize the composition and proportion (α) that each donor group contributed to the sources of the admixture event (Supplemental Information). We can infer the date of admixture by modeling the decay of LD between ancestral chunks, which decreases more rapidly the longer ago admixture occurred [11, 22]. GLOBETROTTER reconstructs admixture sources as mixtures of the available donor groups, which allows one to infer the properties of admixture

 ©2015 Elsevier Ltd All rights reserved

when the donor groups are themselves admixed, making it particularly suited to the current setting. We attempted to infer admixture in all 82 West Eurasian clusters, but, with the exception of a Finnish cluster (finni3) that contained both of the Finnish individuals in the analysis (together with a Norwegian), to allow the algorithm to concentrate on identifying admixture from genetically well-defined donor groups, we removed all clusters with fewer than five individuals from being admixture donors, all of which were sub-groups of larger populations (Table S3).

Admixture Is Common in West Eurasia

The vast majority of clusters (78%; 64 out of 82) showed evidence of admixture, suggesting that admixture-facilitated gene flow is a fundamental property of almost all West Eurasian groups (Tables S4 and S5; Supplemental Information). Here, we discuss the broader patterns of ancestry across West Eurasia, with a more detailed assessment of admixture events provided in the Supplemental Information. Throughout, we refer to the inferred groups characterized by GLOBETROTTER as contributing to an admixture event as "sources" and the sampled groups contributing ancestry to these sources as "donors." It is also important to note that in the discussion presented below, we use current-day geographic labels to describe ancestry of historical sources of admixture. When we describe the ancestry of a particular source as, for example, "Mongolian," this is a convenient but less precise proxy for "ancestry in a historical group that is related to the ancestry that we observe in contemporary Mongolian populations today." This shorthand aids reading, but one must bear in mind that while the inferred sources of admixture are likely to be closely related genetically to the true historical admixing groups, because of subsequent population movements and migration, they may be less closely related geographically to the original source of that ancestry.

To visualize ancestry across West Eurasia, we constructed circos plots [23] where each segment of the circle represents a recipient group. These summaries describe the recent ancestry of the clusters: each admixture source is colored by contributions from different donor groups. We can then compare these mixed sources to the set of admixture donors to find the best-matching present-day donor group that is connected to events by links across the middle of the circles (Figures 2 and S3; Table S4). For any given event, based on the compositions of the sources, we identify the best-matching major admixture source, which is always most similar to a West Eurasian donor group, and the best-matching minor admixture source, which can be most similar to either a West Eurasian or World Region donor, and therefore define events in this way. The barplots in Figure 2B show that almost all of West Eurasia has some ancestry from the World Regions. Such World Region ancestry can be seen in the composition of sources involved in events in northern European groups (NWE and NEE), yet only three of the clusters containing individuals from this region derive ancestry from a source best matched by a World Region donor. Deconstruction of the admixture events in these northern European clusters shows that most mixing involves groups already present within West Eurasia (Figures 2C and S3). Assuming a generation time of 29 years [24], dates for these events center around the late first millennium CE, a time known to have involved significant upheaval in Europe (Figure 2B) [25].

Recent Gene Flow into West Eurasia from Surrounding World Regions

In contrast to relatively low levels in Northern Europe, ancestry from East Asia is much more visible in the West Central Asian, Caucasus, and Turkish clusters, where the influence of Mongolia (mon) in particular can be seen through the pink links and bars in Figure 2B and in Figure 4A. In West Central Asia (WA), some Central (cas) and East Asian (eas) ancestry is also present across this region. Within Anatolia (here defined as Armenia and Iran, IA, and Turkey, TK), West Central Asia (WA; including Nogai, Tajik, and Turkmen individuals), and several other groups from the Caucasus (EC and WC), events largely involve Asian sources, with the period after 1000 CE appearing to be important in the generation of the ancestry of this region (Figure 3B). Interestingly, the three events that do involve a Mongolian-like source in Northern Europe, in the Chuvash (CH; chuva16: 829 CE [627–940 CE]), Russians (russi25: 913 CE [754–1007 CE]), and Mordovians (mordo13: 792 CE [564–975 CE]) all date prior to 1000 CE, suggesting an origin from a different historical event to the more eastern groups (Figure 3B). Of the other Asian world regions, we only see direct admixture from North Siberia (nsib) into a Finnish cluster (finni3: 469 CE [213 BCE–1011 CE]; Figure 3B and Table S4) and from India (ind) into a cluster of two Romanians (roman2: 990 CE [741–1245 CE]), putatively of Romany origin. Nevertheless, observable ancestral components from Afghanistan and Pakistan groups (afp and bal) in WA, EC, WC, and IA suggests that ancestry from across Asia is shared with the more easterly West Eurasian groups.

Southern European groups (SEE, SCE, SDN, SWE, and BA) on the other hand derive ancestry from African and Near Eastern World Regions. In particular, ancestry from groups most similar to contemporary populations from in and around the Levant (lev; which we define as the World Region containing individuals from Syria, Palestine, Lebanon, Jordan, Saudi, Yemen, and Egypt) is present across Italy (SCE), Sardinia (SDN), France and Spain (SWE), and Armenia (IA; Figure 2B). Interestingly, North (nafII) and West (waf) African ancestry is also seen entering Southern Europe, suggesting a key role for the Mediterranean in supporting gene flow back into Europe [8, 26, 27]. Dates for the influx of this admixture are broad and generally fall within the first millennium CE (Figure 3B) although are more recent in BA and SWE, including French (frenc24: 728 CE [424–1011 CE]) and Spanish (spani27: 1042 CE [740–1201 CE]; spani9: 668 CE [286–876 CE]) clusters, consistent with migrations associated with the Arabic Conquest of the Iberian peninsula [8, 11, 28] and earlier movements in and around Italy [29].

Movement within Europe during the Medieval Migration Period

When we consider the composition of sources from within West Eurasia (minor sources in Figure 2C and major sources in Figure 2D), while the majority of a group's ancestry tends to come from its own regional area, there is a substantial contribution of both Northern European (light and dark blue) and Armenian groups (light green) to most WA, EC, WC, and TK clusters, as well as some clusters from both SEE and SCE. As previously reported [11], the formation of the Slavic people at around 1000 CE had a significant impact on the populations of Northern and Eastern Europe, a result that is supported by an analysis of

 ©2015 Elsevier Ltd All rights reserved

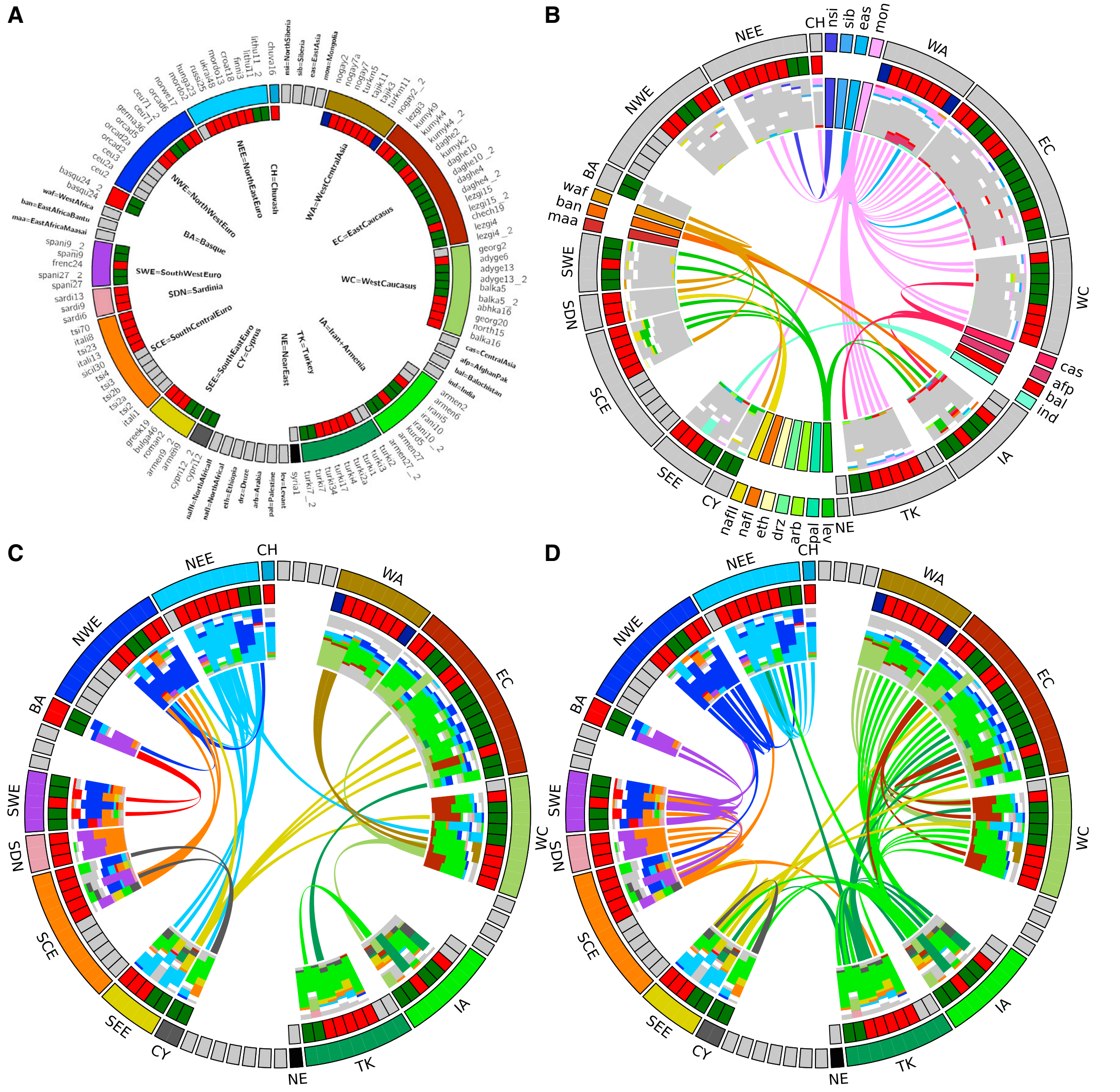

Figure 2. Summary of Eurasian Admixture Events Inferred by GLOBETROTTER

(A) Key showing the position of each cluster in the circos plot. The inner circle describes the type of event inferred in each cluster: gray = no admixture; red = one date; green = one date multiway, blue = two dates. For the latter two types of events, two sets of sources are shown. Second event sources are suffixed with a 2. Clusters are ordered clockwise by increasing date within regions around the circle. Labels for plots B–D are shown in bold in (A) for West Eurasian source regions inside the circle and World Regions sources around the edge.

(B) All events involving minor World Region sources. For each event, the two sources are shown as barplots; each source is split by whitespace, and the size of the two sources reflects the proportion that that source contributes to the admixture event. Each source is made up from a number of components whose colors reflect the World Region that the source component comes from. All Eurasian source components are grayed out. Although made up of components, each source can also be represented by a "best-matching" source, and the central links join the best-matching source (thick end of the link) to the recipient cluster (thin end).

(C and D) Equivalent plots to (B) showing West Eurasian admixture components in color and World Region components in gray. Links in (C) join the best-matching minor West Eurasian sources to the clusters. Links in (D) join the best-matching major admixture source, which is always from West Eurasia, to the relevant cluster. Colors in (C) and (D) represent different regions to those in (B).

identity by descent segments in European populations [10]. Here, despite characterizing populations by genetic similarity rather than geographic labels, we infer the same events involving a "Slavic" source (represented here by a cluster of Lithuanians; lithu11 and colored light blue) across all Balkan groups in the analysis (Greece, Bulgaria, Romania, Croatia, and Hungary) as well as in a large cluster of Germanic origin (germa36) and a composite cluster of eastern European individuals (ukrai48; Figures 4A and 4B). Dates for these events mostly overlap, although are older in Croatia and Greece, and appear to concentrate at the end of the first millennium CE (Figure 2B), a time known as the European Migration Period, or Völkerwanderung [25]. We additionally infer events during the period 300–1200 CE across Northern and Western Europe involving minor West Eurasian

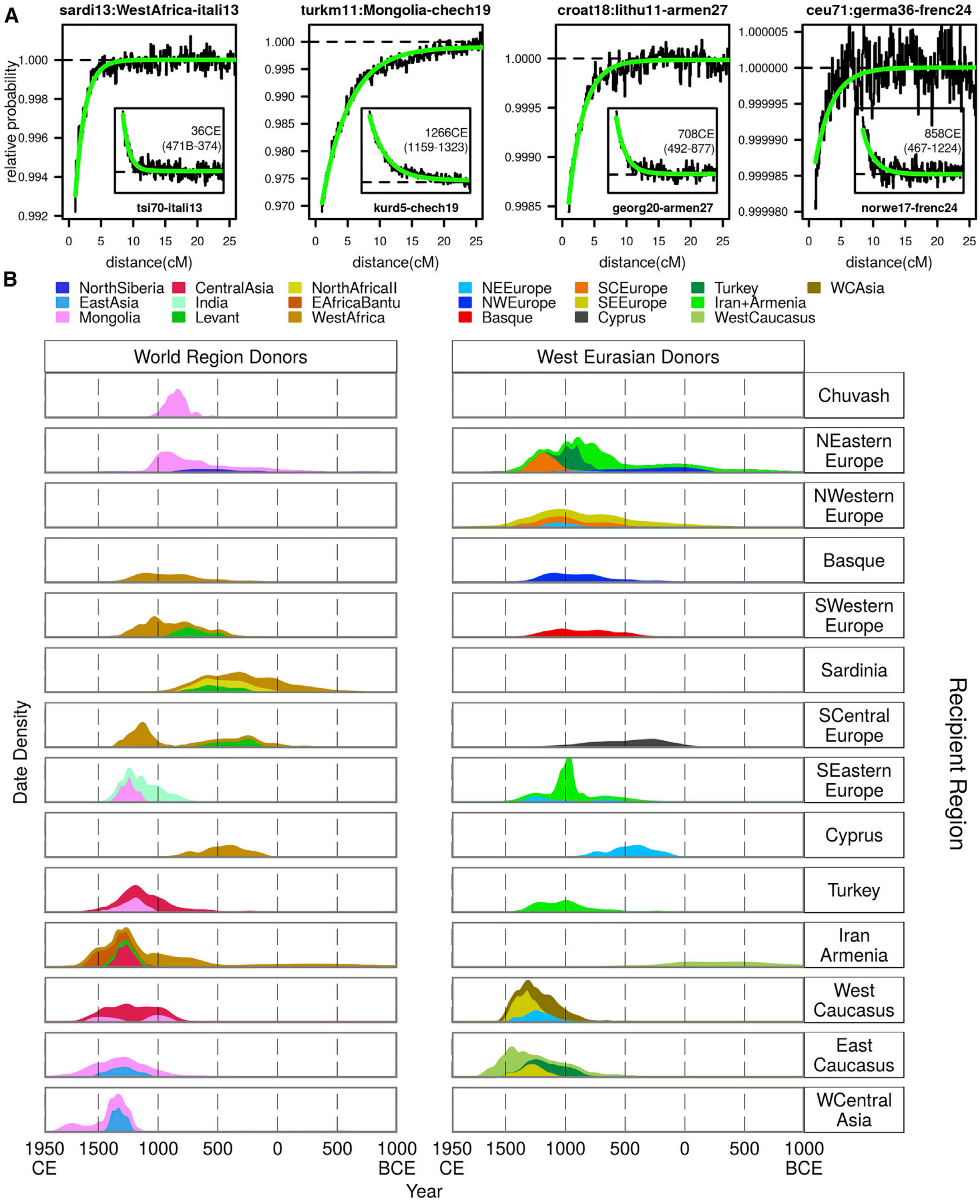

Figure 3. Dates of Eurasian Admixture Events Inferred by GLOBETROTTER

(A) Example co-ancestry curves that we use to infer the date of admixture and composition of sources. For a given cluster, CHROMOPAINTER identifies the chunks of DNA within each individual's genome that are most closely related ancestrally to each donor group. GLOBETROTTER measures the decay of association versus genetic distance between the chunks copied from a given pair of donor groups. Assuming a single pulse of admixture between two or more distinct admixing source groups, theoretical considerations predict that this decay will be exponentially distributed with rate equal to the time (in generations) that this admixture occurred [22]. GLOBETROTTER jointly fits an exponential distribution to the decay curves for all pairwise combinations of donor groups and determines the single best fitting rate, hence determining the most likely single admixture event and estimating the date it occurred. GLOBETROTTER aims to infer the haplotype composition of each source group for the admixture as a linear combination of those carried by sampled groups. This results in the admixed groups themselves automatically being represented in the same form—as a mixture of mixtures. The left-most plot of the four large plots shows the relative probability of jointly copying two chunks from West Africa and North Italian (itali13) donors, at varying genetic distances, in a Sardinian cluster (sardi13). The curves closely fit an exponential decay (green line) with a rate of 65 generations, or 36 CE. The negative slope for this WestAfrica-itali13 curve suggests that these

(legend continued on next page)

 ©2015 Elsevier Ltd All rights reserved

source groups from Europe (Figures 2D, 3B, and 4C). The date and composition of these events suggest a substantial amount of movement during the Völkerwanderung [25], providing persuasive evidence that this period had a visible effect on contemporary populations across Northern, Western, and Central Europe (Figure 4C).

The Effect of Recent Admixture on Genomic Variation in West Eurasia

All peripheral populations analyzed have experienced recent admixture from World Regions (Figure 4A), and we also inferred recent mixing between many of the groups within West Eurasia (Figure 4C). We performed a variety of analyses using total variation distance (TVD) to understand and quantify the effect of these events on genetic variation (Figure 4 and Supplemental Information). Using the output of GLOBETROTTER, we considered "pre-admixture" variation in two ways: by using the inferred major source copying vectors directly and by removing the minor admixing source from the original cluster copying vector. Likewise, "post-admixture" variation can be inferred either by combining the inferred major and minor admixture sources in the appropriate admixture proportions or by using the cluster copying vectors directly. While the two sets of pre- and post-admixture copying vectors should be similar, in practice, they are unlikely to be identical, both because the admixture inference is unlikely to be perfect and because GLOBETROTTER is unable to fully account for genetic drift that may have occurred after admixture [11]. Comparisons of TVD between pairs of copying vectors inferred in these two ways show that when we re-generate a cluster from the admixture inference (Figure S4J), we systematically underestimate variation compared to the variation we observe when we use the contemporary clusters. In fact, for a given group, the differences between the two pre-admixture copying vectors and the two post-admixture copying vectors are highly correlated (Figure S4K), suggesting that the variation is mainly down to differences between the (observed) cluster copying vectors and GLOBETROTTER's inferred sources of admixture. If this error is in part due to drift, then this suggests that drift after admixture may have acted to increase genetic differentiation.

When we compare the relative differences between pre- and post-admixture groups, we observe no appreciable difference between them, suggesting that admixture has not had a significant impact on genetic variation in West Eurasia (Figure 4G). Median TVD does, however, marginally decrease in the pre-admixture variation estimates (Figure 4G), which appears to be driven by differences between western (top left quadrant of Figures 4E and 4F) and eastern (lower right quadrant) West Eurasian groups. When we plot all admixture sources on a PCA based on contemporary individuals (Figure 4D), they tend to occur closer to the center of the plot, resembling the West Eurasian population structure inferred in a recent study of Bronze Age individuals [6]. Additional recent research using ancient DNA from multiple populations and time points in West Eurasia has demonstrated that there has been large-scale genetic turnover in Europe over the last 5,000 years [4–6, 30]. Our analysis supports this work by providing evidence that recent population movements have acted on top of this Bronze Age structure but also highlights a potential role for admixture and/or genetic drift in contributing to the genetic variation present in West Eurasia today.

Our results show that it is possible to draw complex inferences about recent human evolutionary past through the genomes of people alive today that are complementary to those made from ancient DNA. We caution that we are unlikely to have included individuals from all potential genetic donor groups to the current West Eurasian gene pool, and therefore, the gene flow events that we present should be viewed in the context of the dataset that we have used. Future work providing a better understanding of the phenotypic effects of World Region ancestry on contemporary populations as well as placing this work within the context of ancient DNA samples will further aid our understanding of Eurasian prehistory and disease. Nonetheless, the current analysis demonstrates that admixture has left a record in the genomes of all contemporary West Eurasians.

EXPERIMENTAL PROCEDURES

Dataset

Our dataset included 40 newly genotyped individuals (20 each from Croatia and Daghestan) together with published data, choosing samples on the basis of shared genotyping platform (Illumina 550, 610, 660W) and relevance to the peopling of Western Eurasia [11, 15–17, 31] (Figure 1; Table S1). All datasets and genetic maps were based on build 36 of the human genome. We merged the datasets using PLINK (v.1.07) [32], and individuals and SNPs with call rates of less than 98% were dropped. Further quality control to remove cryptically related individuals based on identity by descent (IBD) and PCA was also performed. The final dataset contained 2,192 individuals from 144 populations typed on 477,812 SNPs (Table S1), which were computationally phased together using SHAPEITv1 [33]. Individuals who provided samples gave informed consent following ethical approval by the ethics committees at the various universities where the samples were collected.

Defining Analysis Clusters

We ran fineSTRUCTURE [14] to cluster individuals and identified 18 World Regions based on this clustering (Figure 1; Table S2). Fixing these groups, we re-ran the algorithm twice, identifying the final list of 82 Eurasian clusters (Table S3) based on comparisons between these two runs. Clusters are therefore based on genetic similarity only (see also Figure S1). PCAs in Figures 1 and S4 were generated by performing a PCA on the CHROMOPAINTER chunk-counts matrix using the *prcomp* function in R [34]. Further details are described in the Supplemental Information.

Inferring Complex Admixture with GLOBETROTTER

We describe the detailed process of inferring admixture with GLOBETROTTER in the Supplemental Information. Briefly, we first used CHROMOPAINTER, a chromosome-painting method that reconstructs each individual genome as

donors contribute to different sides of an admixture event. The inset tsi70-itali13 curve has a positive slope, showing that tsi70 and itali13 contribute to the same side of the admixture event. We show similar pairs of curves for three other groups (turkm11, croat18, and ceu71) with varying dates and donors of admixture. (B) We define each admixture event by the West Eurasian region that the recipient group comes from (rows) and the identity of the best-matching current-day group to the minor admixture source (colors). We show dates separately for events involving World Region minor sources (left, events shown by links in Figure 2B) and West Eurasian minor sources (right, events shown by links in Figure 2C). For each region, all date bootstraps for events involving a best-matching source from the specified donor region are combined to generate a density. The integrals of the densities are proportional to the number of admixture events used to generate them.

 ©2015 Elsevier Ltd All rights reserved

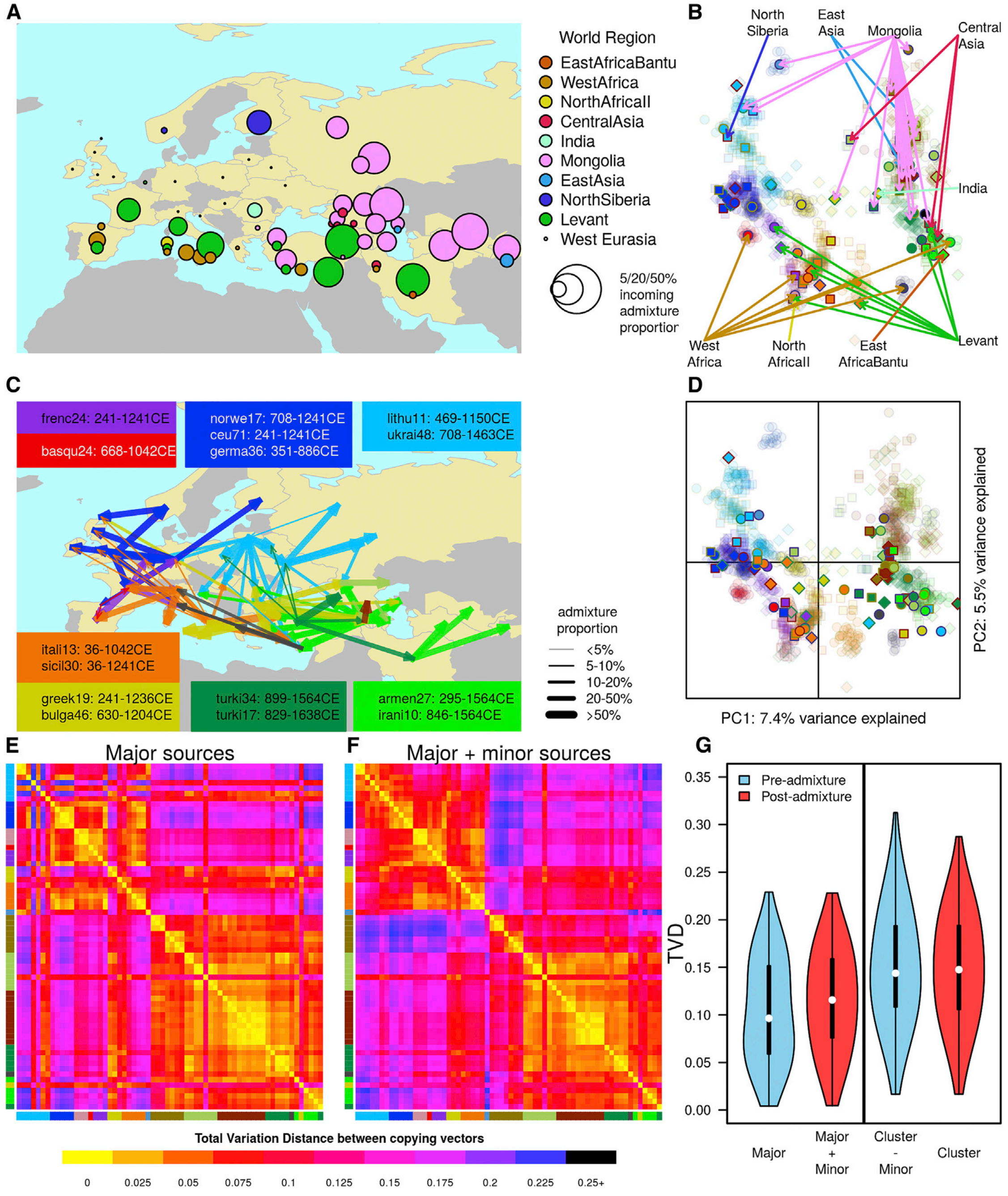

Figure 4. The Impact of Recent Admixture in West Eurasia

(A) For each geographic sampling location, we estimated the proportion of ancestry coming from outside of West Eurasia by averaging GLOBETROTTER's admixture inference across individuals from a sampling location. The sampling locations of each point are shown in Figure S4A; Caucasus populations are spread out to aid visibility. Points are stacked vertically in cases where multiple ancestries are present in a population.

(B) Copying vectors of 82 West Eurasian fineSTRUCTURE clusters projected onto PCA based on the copying vectors of 1,000 West Eurasian individuals (faded colors; symbols and colors are as in Figure 1B); lines link World Region admixture sources to the clusters in which admixture from them is inferred.

(C) Gene flow within West Eurasia is shown by lines linking the best-matching donor group to the sources of admixture with recipient clusters (arrowhead). Line colors represent the regional identity of the donor group, and line thickness represents the proportion of DNA coming from the donor group. Ranges of the dates (point estimates) for events involving sources most similar to selected donor groups are shown.

(D and E) The pre-admixture structure of West Eurasian groups is shown by projecting all admixture source copying vectors that most closely match a West Eurasian group (n = 81) and the cluster copying vectors where we do not infer admixture (n = 18) onto the same PCA as (B). Heatmaps show pairwise total variation distance (TVD) between the Major admixture source copying vectors of all clusters where we infer admixture (n = 64; E) and copying vectors generated by combining the Major and Minor admixture sources at inferred admixture proportions.

(legend continued on next page)

 ©2015 Elsevier Ltd All rights reserved

a mosaic of all donor groups, to identify the subset of donors that share material with the recipient group. Next, because closely related individuals share long stretches of DNA, with the length of these chunks shortening as individuals become less related, we used the paintings to infer the distribution of ancestral chunks at different genetic distances along the genome, and build "coancestry curves" for each pair of putative donor populations (Figure 3A). Assuming a single pulse of admixture involving two genetically distinct sources, the exponential decay of these curves is proportional to the time since genetic material from the two donor groups came together and thus provides a date of the admixture event [11, 22]. Finally, we sequentially removed donor groups from the analysis where such curves were no different from background noise, a step that allowed us to (re-)assess the makeup of the contributing source groups and to identify whether two groups occur on the same side of an admixture event. We performed further tests on these curves, allowing us to assess whether admixture has occurred at multiple times in a group (i.e. we tried to fit multiple exponentials to the coancestry curves) and whether admixture occurred with more than two admixing source groups. We tested the robustness of the admixture inference by comparing these curves with those generated by considering CHROMOPAINTER painting samples from different individuals, leveraging the idea that ancestry LD characteristically decays within individual genomes much more strongly than when ancestry is measured in different individuals (Supplemental Information).

Characterizing Admixture Events and Source Copying Vectors

In cases where we inferred admixture ($p < 0.01$), we then characterized the admixture as one date (1D), one date multiway (1MW), or multiple dates (2D). For each event in each cluster, we inferred the proportion, α and date(s), λ, of admixture together with a set of βs, which describe the composition of the admixing sources. 1D events have two admixing sources; 1MW and 2D events have four admixing sources. To infer copying vectors for the admixture sources, we took the β coefficients for a given source and multiplied each by their respective copying vectors (see the Supplemental Information for a detailed discussion of this approach). In Figure 4, to assay pre-admixture variation, we showed comparisons between major source copying vectors (Major) and clusters with admixture sources removed (Cluster − Minor), and for post-admixture variation, we use the inferred admixed group (Major + Minor) and contemporary cluster copying vectors.

We generated 100 date bootstraps by re-estimating the date of admixture sampling the painted samples from all individuals in a cluster with replacement. In the text, figures, and tables, we converted time in admixture in generations to historical time assuming a generation time of 29 years [24]. In Figure 3, date bootstraps are combined across all events involving best-matching sources from a given region and then grouped by the region that the target cluster comes from.

Comparing Sets of Copying Vectors

We used TVD to compare copying vectors [20]. As the copying vectors are discrete probability distributions over the same set of donors, TVD is a natural metric for quantifying the difference between them. For a given pair of groups A and B with copying vectors describing the copying from i donors, a_i and b_i, we can estimate TVD with the following equation:

$$TVD = 0.5 \times \sum_{i=1}^{n} (|a_i - b_i|)$$

To compare variation in West Eurasia before and after admixture, we estimated TVD for each pair of copying vectors and show the distribution as violin plots (Figure 4G) and boxplots (Figure S4B).

ACCESSION NUMBERS

The accession number for the Croatian samples genotyped for this study is GEO: GSE71603.

SUPPLEMENTAL INFORMATION

Supplemental Information includes Supplemental Experimental Procedures, four figures, and five tables and can be found with this article online at http://dx.doi.org/10.1016/j.cub.2015.08.007.

AUTHOR CONTRIBUTIONS

C.C. and G.B.J.B. conceived and designed the research. G.H., S.M., and G.B.J.B. developed methods. G.B.J.B. and F.M. performed analyses. S.T., K.B., I.R., T.Z., C.H., D.T., S.K.-Y., D.N., P.A., F.C., F.B., V.R., G.L., C.B., J.B.C., A.H.-K., S.D., R.P., P.K., T.H., T.M., R.J.H., and J.F.W. provided DNA samples for genotyping. G.B.J.B. and C.C. wrote the paper, which was reviewed by all authors.

ACKNOWLEDGMENTS

We thank all anonymous donors of DNA. Additionally, we thank the staff of the Unità Operativa Complessa di Medicina Trasfusionale, Azienda Ospedaliera Umberto I, Siracusa, Italy. We are grateful to the John Fell Fund at the University of Oxford (C.C.), the Wellcome Trust (S.M., grant 098387/Z/12/Z), the Wellcome Trust and Royal Society (G.H., grant 098386/Z/12/Z), the Biotechnology and Biological Sciences Research Council (G.B.J.B.), and the British Academy (C.C., grant BARDA-47870) for funding. Request for access to the Daghestan data should be directed to K.B. at kbulayeva@hotmail.com. J.F.W. is a shareholder, employee, and director of the commercial genetic ancestry testing company ScotlandsDNA.

Received: April 1, 2015
Revised: June 29, 2015
Accepted: August 5, 2015
Published: September 17, 2015

REFERENCES

1. Haak, W., Balanovsky, O., Sanchez, J.J., Koshel, S., Zaporozhchenko, V., Adler, C.J., Der Sarkissian, C.S., Brandt, G., Schwarz, C., Nicklisch, N., et al.; Members of the Genographic Consortium (2010). Ancient DNA from European early neolithic farmers reveals their near eastern affinities. PLoS Biol. *8*, e1000536.
2. Skoglund, P., Malmström, H., Raghavan, M., Storå, J., Hall, P., Willerslev, E., Gilbert, M.T., Götherström, A., and Jakobsson, M. (2012). Origins and genetic legacy of Neolithic farmers and hunter-gatherers in Europe. Science *336*, 466–469.
3. Malmström, H., Gilbert, M.T.P., Thomas, M.G., Brandström, M., Storå, J., Molnar, P., Andersen, P.K., Bendixen, C., Holmlund, G., Götherström, A., and Willerslev, E. (2009). Ancient DNA reveals lack of continuity between neolithic hunter-gatherers and contemporary Scandinavians. Curr. Biol. *19*, 1758–1762.
4. Lazaridis, I., Patterson, N., Mittnik, A., Renaud, G., Mallick, S., Kirsanow, K., Sudmant, P.H., Schraiber, J.G., Castellano, S., Lipson, M., et al. (2014). Ancient human genomes suggest three ancestral populations for present-day Europeans. Nature *513*, 409–413.
5. Haak, W., Lazaridis, I., Patterson, N., Rohland, N., Mallick, S., Llamas, B., Brandt, G., Nordenfelt, S., Harney, E., Stewardson, K., et al. (2015). Massive migration from the steppe was a source for Indo-European languages in Europe. Nature *522*, 207–211.
6. Allentoft, M.E., Sikora, M., Sjögren, K.G., Rasmussen, S., Rasmussen, M., Stenderup, J., Damgaard, P.B., Schroeder, H., Ahlström, T., Vinner, L.,

(F) In cases where we infer one date multiway or two dates, we show the major source for the first and/or most recent event only. Clusters are in the same order from top to bottom as in the tree in Figure 1, and axis colors describe the geographical origin of the cluster.
(G) Violin plots comparing the distribution of TVD between the same two sets of copying vectors. The white point indicates the median value; the box shows the 25–75 percentiles; and the plots are truncated at the 2.5 and 97.5 percentiles. The colored shapes show kernel densities.

 ©2015 Elsevier Ltd All rights reserved

et al. (2015). Population genomics of Bronze Age Eurasia. Nature *522*, 167–172.

7. Brandt, G., Haak, W., Adler, C.J., Roth, C., Szécsényi-Nagy, A., Karimnia, S., Möller-Rieker, S., Meller, H., Ganslmeier, R., Friederich, S., et al.; Genographic Consortium (2013). Ancient DNA reveals key stages in the formation of central European mitochondrial genetic diversity. Science *342*, 257–261.
8. Moorjani, P., Patterson, N., Hirschhorn, J.N., Keinan, A., Hao, L., Atzmon, G., Burns, E., Ostrer, H., Price, A.L., and Reich, D. (2011). The history of African gene flow into Southern Europeans, Levantines, and Jews. PLoS Genet. *7*, e1001373.
9. Patterson, N., Moorjani, P., Luo, Y., Mallick, S., Rohland, N., Zhan, Y., Genschoreck, T., Webster, T., and Reich, D. (2012). Ancient admixture in human history. Genetics *192*, 1065–1093.
10. Ralph, P., and Coop, G. (2013). The geography of recent genetic ancestry across Europe. PLoS Biol. *11*, e1001555.
11. Hellenthal, G., Busby, G.B.J., Band, G., Wilson, J.F., Capelli, C., Falush, D., and Myers, S. (2014). A genetic atlas of human admixture history. Science *343*, 747–751.
12. Novembre, J., Johnson, T., Bryc, K., Kutalik, Z., Boyko, A.R., Auton, A., Indap, A., King, K.S., Bergmann, S., Nelson, M.R., et al. (2008). Genes mirror geography within Europe. Nature *456*, 98–101.
13. Lao, O., Lu, T.T., Nothnagel, M., Junge, O., Freitag-Wolf, S., Caliebe, A., Balascakova, M., Bertranpetit, J., Bindoff, L.A., Comas, D., et al. (2008). Correlation between genetic and geographic structure in Europe. Curr. Biol. *18*, 1241–1248.
14. Lawson, D.J., Hellenthal, G., Myers, S., and Falush, D. (2012). Inference of population structure using dense haplotype data. PLoS Genet. *8*, e1002453.
15. Rasmussen, M., Li, Y., Lindgreen, S., Pedersen, J.S., Albrechtsen, A., Moltke, I., Metspalu, M., Metspalu, E., Kivisild, T., Gupta, R., et al. (2010). Ancient human genome sequence of an extinct Palaeo-Eskimo. Nature *463*, 757–762.
16. Behar, D.M., Yunusbayev, B., Metspalu, M., Metspalu, E., Rosset, S., Parik, J., Rootsi, S., Chaubey, G., Kutuev, I., Yudkovsky, G., et al. (2010). The genome-wide structure of the Jewish people. Nature *466*, 238–242.
17. Yunusbayev, B., Metspalu, M., Järve, M., Kutuev, I., Rootsi, S., Metspalu, E., Behar, D.M., Varendi, K., Sahakyan, H., Khusainova, R., et al. (2012). The Caucasus as an asymmetric semipermeable barrier to ancient human migrations. Mol. Biol. Evol. *29*, 359–365.
18. Alexander, D.H., Novembre, J., and Lange, K. (2009). Fast model-based estimation of ancestry in unrelated individuals. Genome Res. *19*, 1655–1664.
19. Price, A.L., Patterson, N.J., Plenge, R.M., Weinblatt, M.E., Shadick, N.A., and Reich, D. (2006). Principal components analysis corrects for stratification in genome-wide association studies. Nat. Genet. *38*, 904–909.
20. Leslie, S., Winney, B., Hellenthal, G., Davison, D., Boumertit, A., Day, T., Hutnik, K., Royrvik, E.C., Cunliffe, B., Lawson, D.J., et al.; Wellcome Trust Case Control Consortium 2; International Multiple Sclerosis Genetics Consortium (2015). The fine-scale genetic structure of the British population. Nature *519*, 309–314.
21. Montinaro, F., Busby, G.B.J., Pascali, V.L., Myers, S., Hellenthal, G., and Capelli, C. (2015). Unravelling the hidden ancestry of American admixed populations. Nat. Commun. *6*, 6596.
22. Falush, D., Stephens, M., and Pritchard, J.K. (2003). Inference of population structure using multilocus genotype data: linked loci and correlated allele frequencies. Genetics *164*, 1567–1587.
23. Krzywinski, M., Schein, J., Birol, I., Connors, J., Gascoyne, R., Horsman, D., Jones, S.J., and Marra, M.A. (2009). Circos: an information aesthetic for comparative genomics. Genome Res. *19*, 1639–1645.
24. Fenner, J.N. (2005). Cross-cultural estimation of the human generation interval for use in genetics-based population divergence studies. Am. J. Phys. Anthropol. *128*, 415–423.
25. Heather, P. (2009). Empires and Barbarians: Migration, Development and the Birth of Europe (London: Macmillan).
26. Auton, A., Bryc, K., Boyko, A.R., Lohmueller, K.E., Novembre, J., Reynolds, A., Indap, A., Wright, M.H., Degenhardt, J.D., Gutenkunst, R.N., et al. (2009). Global distribution of genomic diversity underscores rich complex history of continental human populations. Genome Res. *19*, 795–803.
27. Botigué, L.R., Henn, B.M., Gravel, S., Maples, B.K., Gignoux, C.R., Corona, E., Atzmon, G., Burns, E., Ostrer, H., Flores, C., et al. (2013). Gene flow from North Africa contributes to differential human genetic diversity in southern Europe. Proc. Natl. Acad. Sci. USA *110*, 11791–11796.
28. Roberts, J. (2007). The New Penguin History of the World, Fifth Edition (London: Penguin Books).
29. Metcalfe, A. (2009). The Muslims in Medieval Italy (Edinburgh: Edinburgh University Press).
30. Brotherton, P., Haak, W., Templeton, J., Brandt, G., Soubrier, J., Jane Adler, C., Richards, S.M., Sarkissian, C.D., Ganslmeier, R., Friederich, S., et al.; Genographic Consortium (2013). Neolithic mitochondrial haplogroup H genomes and the genetic origins of Europeans. Nat. Commun. *4*, 1764.
31. Hodoğlugil, U., and Mahley, R.W. (2012). Turkish population structure and genetic ancestry reveal relatedness among Eurasian populations. Ann. Hum. Genet. *76*, 128–141.
32. Purcell, S., Neale, B., Todd-Brown, K., Thomas, L., Ferreira, M.A., Bender, D., Maller, J., Sklar, P., de Bakker, P.I., Daly, M.J., and Sham, P.C. (2007). PLINK: a tool set for whole-genome association and population-based linkage analyses. Am. J. Hum. Genet. *81*, 559–575.
33. Delaneau, O., Marchini, J., and Zagury, J.F. (2012). A linear complexity phasing method for thousands of genomes. Nat. Methods *9*, 179–181.
34. R Development Core Team (2011). R: A language and environment for statistical computing (R Foundation for Statistical Computing). http://www.r-project.org.

 ©2015 Elsevier Ltd All rights reserved

Current Biology
Supplemental Information

The Role of Recent Admixture in Forming the Contemporary West Eurasian Genomic Landscape

George B.J. Busby, Garrett Hellenthal, Francesco Montinaro, Sergio Tofanelli, Kazima Bulayeva, Igor Rudan, Tatijana Zemunik, Caroline Hayward, Draga Toncheva, Sena Karachanak-Yankova, Desislava Nesheva, Paolo Anagnostou, Francesco Cali, Francesca Brisighelli, Valentino Romano, Gerard Lefranc, Catherine Buresi, Jemni Ben Chibani, Amel Haj-Khelil, Sabri Denden, Rafal Ploski, Pawel Krajewski, Tor Hervig, Torolf Moen, Rene J. Herrera, James F. Wilson, Simon Myers, and Cristian Capelli

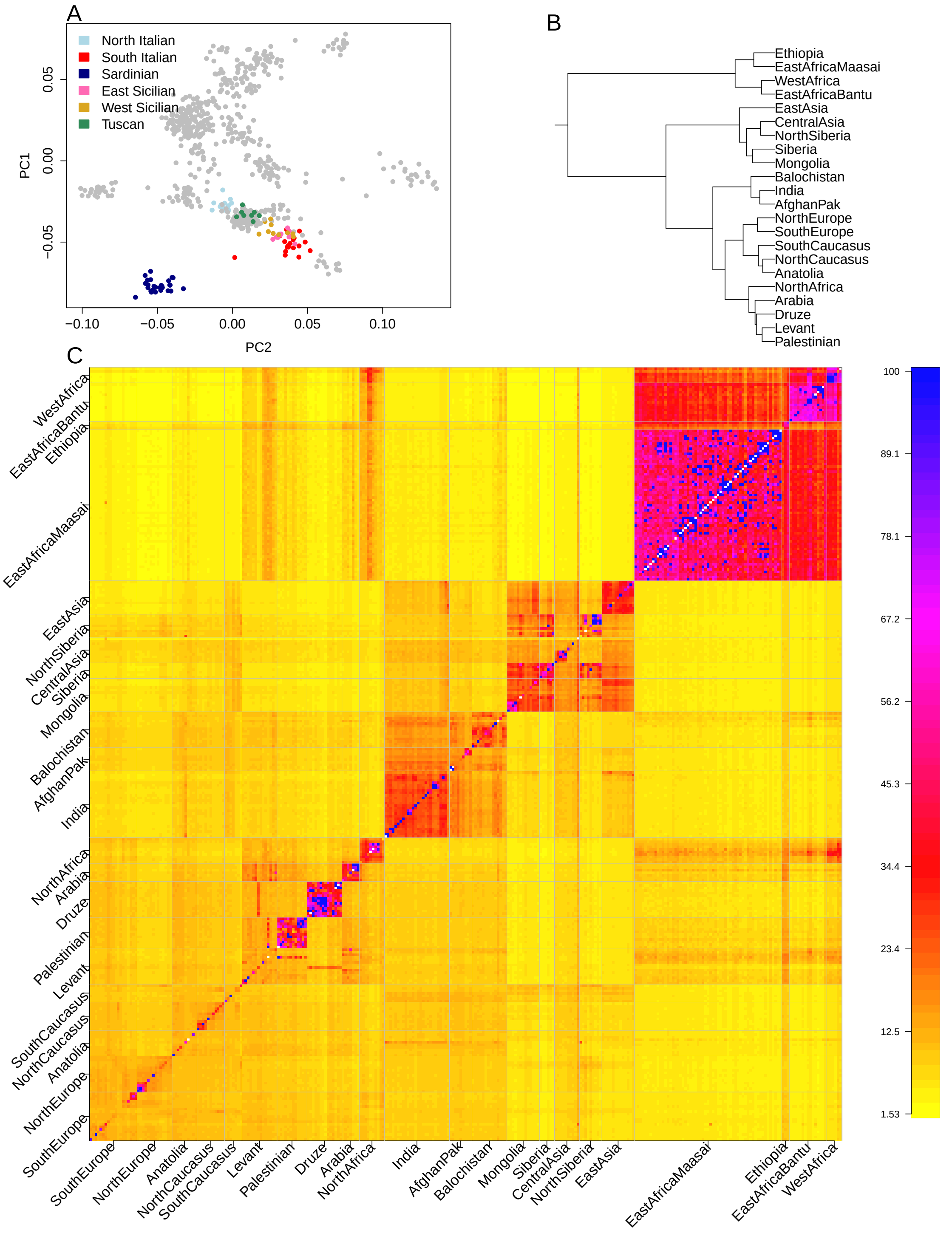

Figure S1. Identification of genetic populations and world regions, related to Figure 1. (A) PCA plot of European SNP genotypes with some Italian populations highlighted shows differentiation between Italian sub-populations (B) The collapsed fineSTRUCTURE tree generated by successively merging groups to generate world regions. (C) The CHROMOPAINTER chunkcount coancestry matrix ordered by the result from the full fineSTRUCTURE analysis based on the worldwide analysis of 2192 individuals which we use to define our analysis clusters. Each row of the heatmap represents a copying vector, with the number of chunks copied from each donor individual as columns. Individuals are ordered by world region and the heatmap is capped at 100 chunks.

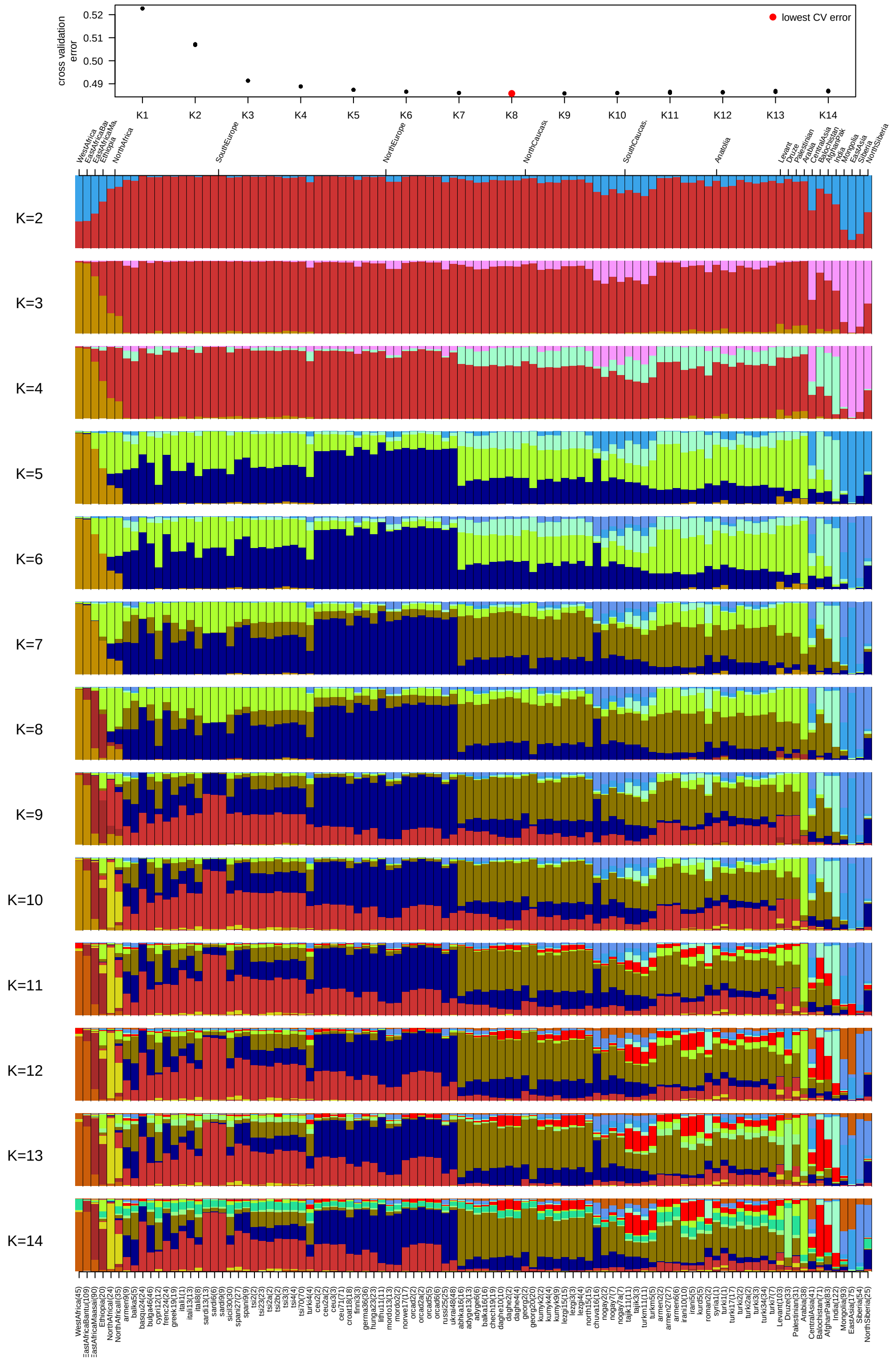

Figure S2. An ADMIXTURE analysis of the dataset showing the average admixture proportions for each cluster and world region. The top plot shows the cross validation error across multiple runs picking 8 as the optimum number of clusters. The number of individuals in each cluster world/region is in parentheses after the cluster/region name. We used an LD (R^2) threshold of 0.2.

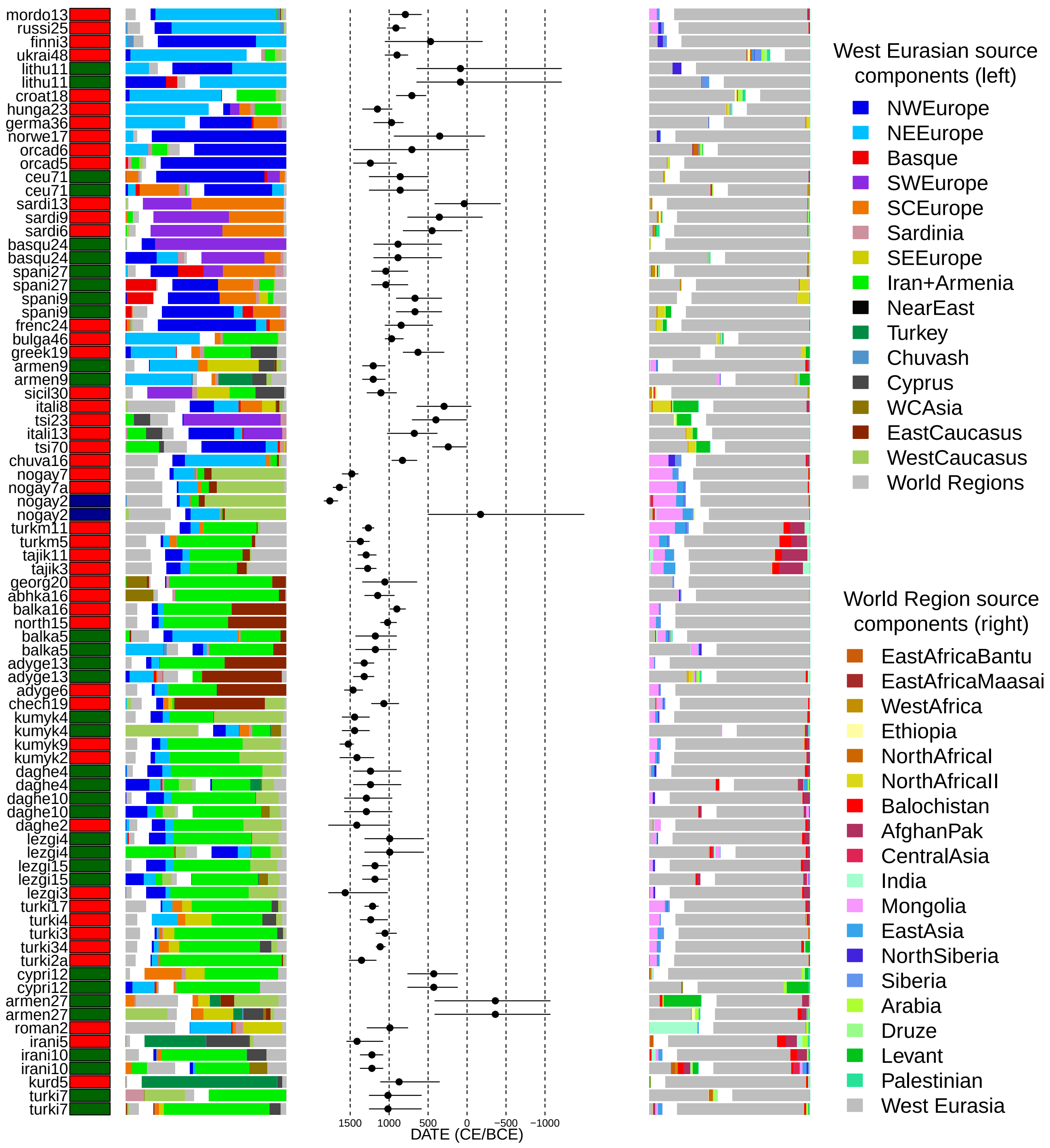

Figure S3. Proportions and dates of admixture shown in Figures 2 and 3. For each cluster we show the result of admixture inference, red = 1D (one date of admixture), darkgreen = 1MW (one date, multiple admixing groups), and darkblue = 2D (two admixture dates). Proportions of the two admixing sources of either side of an admixture event are shown as barplots with their components coloured by donor region. In the left-hand barplot, all non-West Eurasian components are greyed out, whereas the opposite is true for the right hand barplots. The colours of the bars represent the ancestry components detailed in the legends on the right. The date of admixture, with bootstrap CI is also shown in the central plot.

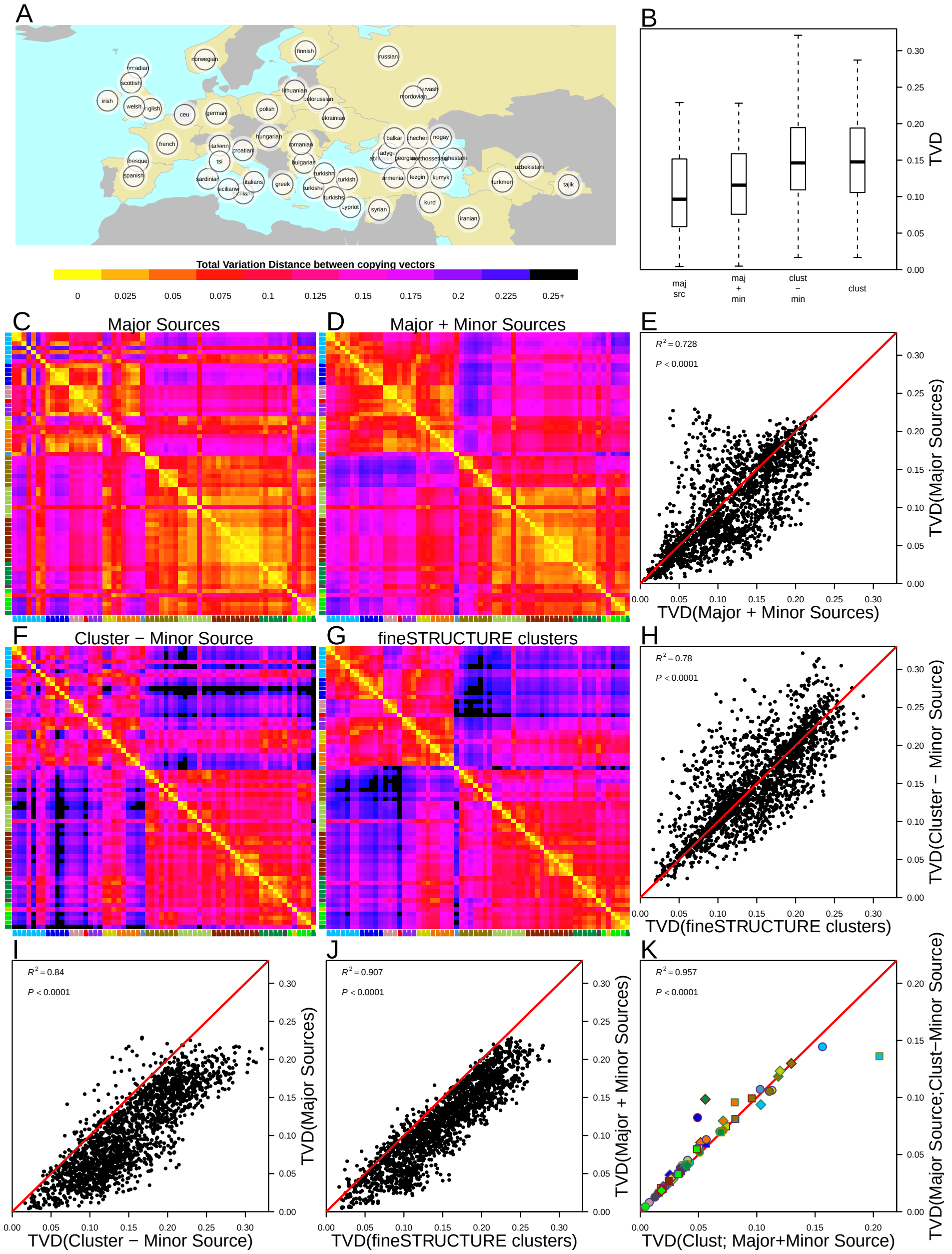

Figure S4. Additional plots related to Figure 4. (A) The geographic position of all populations used to generate Figure 4. For the 64 clusters where we infer admixture we show (B) boxplot showing the distibution of Total Variation Distance (TVD) for four sets of copying vectors: maj src = Major sources; maj + min = copying vectors generated by combining the major and minor source of admixture at inferred admixture proportions; clust - min = the fineSTUCTURE cluster copying vectors with the minor source of admixture removed; and clust = the fineSTRUCTURE copying vectors. (C,D,F,G) For the same four groups we show heatmaps of pairwise TVD. (E,H,I,J) pairwise comparisons of TVD computed separately for the four groups shows that variation tends to be higher when considering the fineSTRUCTURE clusters. (K) For each cluster where we infer admixture we show, the TVD between a copying vector generated from adding the major and minor sources together and the original fineSTRUCTURE cluster ("post-admixture"; x-axis) against the TVD of a copying vector generated by removing the minor source from the fineSTRUCTURE cluster copying vector and the major source of admixture ("pre-admixture"; y-axis). R^2 correlation coefficients and P-values (t-test, 62 degrees of freedom) are shown for all comparisons.

Table S1. Overview of sampled populations describing the continent, region, numbers of individuals used, and the source of any previously published datasets.

Population	Continent	Region	n(pre-QC)	n(post-QC)	Source
bantusa	Africa	SubAfrica	8	8	Li, et al 2008
luhya	Africa	SubAfrica	110	94	HAPMAP
maasai	Africa	SubAfrica	156	97	HAPMAP
mandenka	Africa	SubAfrica	22	21	Li, et al 2008
yoruba	Africa	SubAfrica	21	21	Li, et al 2008
ethiopiana	Africa	NorthAfrica	7	7	Behar, et al 2010
ethiopiano	Africa	NorthAfrica	7	7	Behar, et al 2010
ethiopiant	Africa	NorthAfrica	5	5	Behar, et al 2010
egyptian	Africa	NorthAfrica	12	12	Behar, et al 2010
moroccan	Africa	NorthAfrica	25	25	Hellenthal, et al 2014; Behar, et al 2010
mozabite	Africa	NorthAfrica	29	28	Li, et al 2008
tunisian	Africa	NorthAfrica	16	9	Hellenthal, et al 2014
chechen	Eurasia	EastCaucasus	20	20	Yunusbayev, et al 2011
daghestani/tabasaran	Eurasia	EastCaucasus	20	20	current study
kumyk	Eurasia	EastCaucasus	14	14	Yunusbayev, et al 2011
lezgin	Eurasia	EastCaucasus	18	18	Behar, et al 2010
abhkasian	Eurasia	WestCaucasus	20	20	Yunusbayev, et al 2011
adygei	Eurasia	WestCaucasus	17	17	Yunusbayev, et al 2011
balkar	Eurasia	WestCaucasus	19	19	Yunusbayev, et al 2011
georgian	Eurasia	WestCaucasus	20	20	Yunusbayev, et al 2011
northossetian	Eurasia	WestCaucasus	15	15	Yunusbayev, et al 2011
armenian	Eurasia	Armenia/Iran	35	35	Yunusbayev, et al 2011
iranian	Eurasia	Armenia/Iran	20	19	Behar, et al 2010
kurd	Eurasia	Armenia/Iran	6	6	Yunusbayev, et al 2011
turkishe	Eurasia	Turkey	23	23	Hodoğlugil et al 2012
turkishn	Eurasia	Turkey	20	20	Hodoğlugil et al 2012
turkishs	Eurasia	Turkey	20	20	Hodoğlugil et al 2012
turkish	Eurasia	Turkey	19	19	Behar, et al 2010
cypriot	Eurasia	Cyprus	12	12	Behar, et al 2010
bedouin	Eurasia	NearEast	46	39	Li, et al 2008
druze	Eurasia	NearEast	42	41	Li, et al 2008
jordanian	Eurasia	NearEast	20	19	Li, et al 2008
lebanese	Eurasia	NearEast	8	5	Behar, et al 2010
palestinian	Eurasia	NearEast	46	39	Li, et al 2008
saudi	Eurasia	NearEast	20	19	Behar, et al 2010
syrian	Eurasia	NearEast	16	15	Behar, et al 2010
uae	Eurasia	NearEast	19	13	Hellenthal, et al 2014
yemeni	Eurasia	NearEast	10	5	Behar, et al 2010
chuvash	Eurasia	Chuvash	17	17	Behar, et al 2010
nogay	Eurasia	WestCentralAsia	16	16	Yunusbayev, et al 2011
tajik	Eurasia	WestCentralAsia	15	15	Yunusbayev, et al 2011
turkmen	Eurasia	WestCentralAsia	15	10	Yunusbayev, et al 2011
hazara	Eurasia	CentralAsia	22	20	Li, et al 2008
uygur	Eurasia	CentralAsia	10	10	Li, et al 2008
uzbekistani	Eurasia	CentralAsia	15	15	Yunusbayev, et al 2011
burusho	Asia	CentralAsia	25	25	Li, et al 2008
kalash	Asia	CentralAsia	23	1	Li, et al 2008
pathan	Asia	CentralAsia	22	22	Li, et al 2008
sindhi	Asia	CentralAsia	24	23	Li, et al 2008
balochi	Asia	CentralAsia	24	23	Li, et al 2008
brahui	Asia	CentralAsia	25	23	Li, et al 2008
makrani	Asia	CentralAsia	25	20	Li, et al 2008
kyrgyz	Asia	CentralAsia	16	16	Hodoğlugil et al 2012
cambodian	Asia	SouthAsia	10	10	Metspalu, et al 2011; Chaubey et al 2012
brahmin	Asia	SouthAsia	11	11	Metspalu, et al 2011; Chaubey et al 2012
gond	Asia	SouthAsia	4	4	Metspalu, et al 2011; Chaubey et al 2012
kshatriya	Asia	SouthAsia	7	7	Metspalu, et al 2011; Chaubey et al 2012
meena	Asia	SouthAsia	1	1	Metspalu, et al 2011; Chaubey et al 2012
bengali	Asia	SouthAsia	1	1	Metspalu, et al 2011; Chaubey et al 2012
bhunjia	Asia	SouthAsia	1	1	Metspalu, et al 2011; Chaubey et al 2012
chamar	Asia	SouthAsia	10	10	Metspalu, et al 2011; Chaubey et al 2012

Continued on next page

Table S1 Continued from previous page

Population	Continent	Region	n(pre-QC)	n(post-QC)	Source
chenchu	Asia	SouthAsia	4	4	Metspalu, et al 2011; Chaubey et al 2012
dharkar	Asia	SouthAsia	12	8	Metspalu, et al 2011; Chaubey et al 2012
dhurwa	Asia	SouthAsia	1	1	Metspalu, et al 2011; Chaubey et al 2012
dusadh	Asia	SouthAsia	10	7	Metspalu, et al 2011; Chaubey et al 2012
hakkipikki	Asia	SouthAsia	4	3	Metspalu, et al 2011; Chaubey et al 2012
kanjar	Asia	SouthAsia	8	5	Metspalu, et al 2011; Chaubey et al 2012
karnataka	Asia	SouthAsia	9	8	Behar, et al 2010
kol	Asia	SouthAsia	17	16	Metspalu, et al 2011; Chaubey et al 2012
kurmi	Asia	SouthAsia	1	1	Metspalu, et al 2011; Chaubey et al 2012
kurumba	Asia	SouthAsia	4	4	Metspalu, et al 2011; Chaubey et al 2012
lambadi	Asia	SouthAsia	1	1	Metspalu, et al 2011; Chaubey et al 2012
malayan	Asia	SouthAsia	1	1	Behar, et al 2010
mawasi	Asia	SouthAsia	1	1	Metspalu, et al 2011; Chaubey et al 2012
meghawal	Asia	SouthAsia	1	1	Metspalu, et al 2011; Chaubey et al 2012
muslim	Asia	SouthAsia	5	5	Metspalu, et al 2011; Chaubey et al 2012
myanmar	Asia	SouthAsia	3	3	Behar, et al 2010
nihali	Asia	SouthAsia	2	2	Metspalu, et al 2011; Chaubey et al 2012
piramalaikallar	Asia	SouthAsia	8	8	Metspalu, et al 2011; Chaubey et al 2012
sakd	Asia	SouthAsia	4	4	Behar, et al 2010
tamilnadu	Asia	SouthAsia	2	2	Metspalu, et al 2011; Chaubey et al 2012
tharus	Asia	SouthAsia	2	2	Metspalu, et al 2011; Chaubey et al 2012
upcaste	Asia	SouthAsia	5	5	Metspalu, et al 2011; Chaubey et al 2012
velamas	Asia	SouthAsia	10	9	Metspalu, et al 2011; Chaubey et al 2012
han	Asia	EastAsia	34	34	Li, et al 2008
hannchina	Asia	EastAsia	10	10	Li, et al 2008
japanese	Asia	EastAsia	28	28	Li, et al 2008
naga	Asia	EastAsia	4	4	Metspalu, et al 2011; Chaubey et al 2012
naxi	Asia	EastAsia	8	7	Li, et al 2008
tu	Asia	EastAsia	10	10	Li, et al 2008
xibo	Asia	EastAsia	9	9	Li, et al 2008
yi	Asia	EastAsia	10	10	Li, et al 2008
dai	Asia	EastAsia	10	10	Li, et al 2008
lahu	Asia	EastAsia	8	6	Li, et al 2008
miao	Asia	EastAsia	10	10	Li, et al 2008
she	Asia	EastAsia	10	9	Li, et al 2008
tujia	Asia	EastAsia	10	10	Li, et al 2008
buryat	Asia	EastAsia	17	15	Metspalu, et al 2011; Chaubey et al 2012
daur	Asia	EastAsia	9	9	Li, et al 2008
hezhen	Asia	EastAsia	8	7	Li, et al 2008
mongolian	Asia	EastAsia	19	19	Li, et al 2008
oroqen	Asia	EastAsia	9	9	Li, et al 2008
yakut	Asia	EastAsia	25	25	Li, et al 2008
altai	Asia	Siberia	13	13	Rasmussen, et al 2010
burya	Asia	Siberia	2	2	Rasmussen, et al 2010
tuva	Asia	Siberia	16	13	Rasmussen, et al 2010
chukchi	Asia	Siberia	15	5	Rasmussen, et al 2010
dolgan	Asia	Siberia	7	7	Rasmussen, et al 2010
evenk	Asia	Siberia	16	12	Rasmussen, et al 2010
ket	Asia	Siberia	2	2	Rasmussen, et al 2010
koryake	Asia	Siberia	18	5	Rasmussen, et al 2010
selkup	Asia	Siberia	10	10	Rasmussen, et al 2010
yukagir	Asia	Siberia	9	4	Rasmussen, et al 2010
nganassan	Asia	Siberia	15	10	Rasmussen, et al 2010
basque	Europe	Basque	24	24	Li, et al 2008
finnish	Europe	NEEurope	2	2	Hellenthal, et al 2014
belorussian	Europe	NEEurope	10	9	Behar, et al 2010
lithuanian	Europe	NEEurope	10	10	Behar, et al 2010
mordovian	Europe	NEEurope	15	15	Behar, et al 2010
polish	Europe	NEEurope	18	17	Hellenthal, et al 2014
russian	Europe	NEEurope	25	25	Li, et al 2008
ukrainian	Europe	NEEurope	20	20	Yunusbayev, et al 2011
german	Europe	NWEurope	30	30	current study
ceu	Europe	NWEurope	59	59	HAPMAP
english	Europe	NWEurope	8	8	Hellenthal, et al 2014

Continued on next page

Table S1 Continued from previous page

Population	Continent	Region	n(pre-QC)	n(post-QC)	Source
irish	Europe	NWEurope	7	7	Hellenthal, et al 2014
norwegian	Europe	NWEurope	18	18	Hellenthal, et al 2014
scottish	Europe	NWEurope	8	6	Hellenthal, et al 2014
welsh	Europe	NWEurope	4	4	Hellenthal, et al 2014
orcadian	Europe	NWEurope	15	15	Li, et al 2008
sardinian	Europe	Sardinia	28	28	Li, et al 2008
italiann	Europe	SCEurope	12	12	Li, et al 2008
tsi	Europe	SCEurope	102	98	HAPMAP
tuscan	Europe	SCEurope	8	8	Li, et al 2008
bulgarian	Europe	SEEurope	31	31	Hellenthal, et al 2014; Yunusbayev, et al 2011
croatian	Europe	NEEurope	20	19	current study
hungarian	Europe	NEEurope	20	19	Behar, et al 2010
romanian	Europe	SEEurope	16	16	Behar, et al 2010
greek	Europe	SEEurope	22	20	Hellenthal, et al 2014
italians	Europe	SCEurope	18	18	Hellenthal, et al 2014
siciliane	Europe	SCEurope	10	10	Hellenthal, et al 2014
sicilianw	Europe	SCEurope	10	10	Hellenthal, et al 2014
french	Europe	SWEurope	28	28	Li, et al 2008
spanish	Europe	SWEurope	36	34	Hellenthal, et al 2014; Behar, et al 2010
144	4	21	2422	2192	

Table S2. Composition of the World Regions identified by the fineSTRUCTURE analysis. For each World Region we report the geographic origin of all individuals within the group. The n(by population) column displays the population followed by the number of individuals from that population.

WorldRegion	n(total)	n(by population)
Palestinian	31	palestinian31
Levant	103	jordanian19 bedouin18 syrian13 egyptian11 druze8 palestinian8 saudi7 lebanese5 uae5 yemeni5 iranian4
Druze	33	druze33
Arabia	38	bedouin21 saudi12 uae5
NorthAfricaI	24	mozabite24
NorthAfricaII	35	moroccan24 tunisian9 mozabite2
Armenia/Iran	126	armenian32 turkishe20 turkish18 iranian15 turkishn15 turkishs15 kurd6 romanian2 syrian2 kumyk1
NorthCaucasus	159	chechen20 daghestani20 abhkasian19 georgian19 lezgin18 balkar17 adygei16 northossetian15 kumyk13 turkish1 turkishe1
WestCentralAsia	62	chuvash16 nogay16 tajik15 turkmen10 uzbekistani4 hazara1
SouthEurope	363	tsi98 spanish34 bulgarian31 sardinian28 french25 basque24 greek20 italians18 romanian13 cypriot12 italiann12 siciliane10 sicilianw10 tuscan8 turkishn5 turkishs5 armenian2 balkar2 turkishe2 abhkasian1 adygei1 ceu1 georgian1
NorthEurope	290	ceu58 german30 russian25 ukrainian20 croatian19 hungarian19 norwegian18 polish17 mordovian15 orcadian15 lithuanian10 belorussian9 english8 irish7 scottish6 welsh4 french3 finnish2 armenian1 chukchi1 chuvash1 koryake1 romanian1
AfghanPak	83	burusho25 pathan22 sindhi18 brahmin7 kshatriya5 balochi2 gond1 kalash1 meena1 uae1
India	122	kol16 chamar10 velamas9 dharkar8 karnataka8 piramalaikallar8 dusadh7 kanjar5 muslim5 upcaste5 brahmin4 chenchu4 kurumba4 sakd4 gond3 hakkipikki3 myanmar3 kshatriya2 nihali2 tamilnadu2 tharus2 bengali1 bhunjia1 dhurwa1 kurmi1 lambadi1 malayan1 mawasi1 meghawal1
Balochistan	71	brahui23 balochi21 makrani20 sindhi5 uae2
Mongolia	93	buryat15 kyrgyz14 altai13 tuva13 mongolian12 daur9 oroqen7 hezhen6 burya2 nganassan1 uygur1
Siberia	54	yakut23 evenk12 nganassan7 dolgan5 koryake2 oroqen2 yukagir2 chukchi1
NorthSiberia	25	selkup10 chukchi3 dolgan2 ket2 koryake2 nganassan2 yakut2 yukagir2
CentralAsia	41	hazara19 uzbekistani11 uygur9 kyrgyz2
EastAsia	175	han34 japanese28 cambodian10 dai10 hannchina10 miao10 tu10 tujia10 yi10 she9 xibo9 mongolian7 naxi7 lahu6 naga4 hezhen1
EastAfricaBantu	109	luhya94 bantusa8 maasai7
WestAfrica	45	mandenka21 yoruba21 mozabite2 moroccan1
EastAfricaMaasai	90	maasai90
Ethiopia	20	ethiopiana7 ethiopiano7 ethiopiant5 egyptian1

Table S3. The final fineSTRUCTURE clusters used in the analysis. ClusterName refers to a unique short name given to each cluster which is named for the population that contributes the most individuals to a cluster; the Region and total number of individuals, which is also referred to in the Cluster Name are also listed. As with Table S2, the n(by population) column refers to the geographic population origin of the individuals in each cluster, with a numerical suffix representing the number of individuals from that population.

ClusterName	Region	n(total)	n(by population)
armen2	Armenia/Iran	2	armenian2
armen27	Armenia/Iran	27	armenian24 syrian1 turkishn1 turkishs1
armen9	Armenia/Iran	9	armenian2 bulgarian2 turkishe2 turkishn2 turkishs1
armen6	Armenia/Iran	6	armenian6
irani10	Armenia/Iran	10	iranian10
irani5	Armenia/Iran	5	iranian5
kurd5	Armenia/Iran	5	kurd5
basqu24	Basque	24	basque24
chuva16	Chuvash	16	chuvash16
cypri12	Cyprus	12	cypriot12
chech19	EastCaucasus	19	chechen19
daghe2*	EastCaucasus	2	daghestani2
daghe4†	EastCaucasus	4	daghestani4
daghe10†	EastCaucasus	10	daghestani9 lezgin1
kumyk2	EastCaucasus	2	kumyk2
kumyk4	EastCaucasus	4	kumyk3 chechen1
kumyk9	EastCaucasus	9	kumyk9
lezgi15	EastCaucasus	15	lezgin10 daghestani5
lezgi3	EastCaucasus	3	lezgin3
lezgi4	EastCaucasus	4	lezgin4
syria1	NearEast	1	syrian1
croat18	NEEurope	18	croatian18
finni3	NEEurope	3	finnish2 norwegian1
hunga23	NEEurope	23	hungarian19 armenian1 croatian1 german1 romanian1
lithu11	NEEurope	11	lithuanian9 belorussian1 polish1
mordo13	NEEurope	13	mordovian13
mordo2	NEEurope	2	mordovian2
russi25	NEEurope	25	russian25
ukrai48	NEEurope	48	ukrainian20 polish16 belorussian8 chukchi1 chuvash1 koryake1 lithuanian1
nogay2	WestCentralAsia	2	nogay2
nogay7	WestCentralAsia	7	nogay7
nogay7a	WestCentralAsia	7	nogay7
tajik11	WestCentralAsia	11	tajik10 turkmen1
tajik3	WestCentralAsia	3	tajik3
turkm11	WestCentralAsia	11	turkmen4 uzbekistani4 tajik2 hazara1
turkm5	WestCentralAsia	5	turkmen5
ceu2	NWEurope	2	ceu2
ceu2a	NWEurope	2	ceu2a
ceu3	NWEurope	3	ceu3
ceu71	NWEurope	71	ceu43 english8 irish7 scottish6 welsh4 french2 german1
germa36	NWEurope	36	german28 ceu8
norwe17	NWEurope	17	norwegian17
orcad2	NWEurope	2	orcadian2
orcad2a	NWEurope	2	orcadian2a
orcad5	NWEurope	5	orcadian5
orcad6	NWEurope	6	orcadian6
sardi13	Sardinia	13	sardinian13
sardi6	Sardinia	6	sardinian6
sardi9	Sardinia	9	sardinian9
itali13	SCEurope	13	italiann12 french1
itali1	SCEurope	1	italians1
itali8	SCEurope	8	italians8
sicil30	SCEurope	30	siciliane10 sicilianw10 italians9 greek1

Continued on next page

*Individuals from Daghestan belong to the Tabasaran ethnic group

Table S3 continued from previous page

ClusterName	Region	n(total)	n(by population)
tsi2	SCEurope	2	tsi2
tsi23	SCEurope	23	tsi23
tsi2a	SCEurope	2	tsi2a
tsi2b	SCEurope	2	tsi2b
tsi3	SCEurope	3	tsi3
tsi4	SCEurope	4	tsi4
tsi70	SCEurope	70	tsi62 tuscan8
bulga46[†]	SEEurope	46	bulgarian29 romanian13 turkishs3 turkishn1
greek19	SEEurope	19	greek19
roman2	SEEurope	2	romanian2
frenc24	SWEurope	24	french23 ceu1
spani27	SWEurope	27	spanish27
spani9	SWEurope	9	spanish7 french2
turki2	Turkey	2	turkish1 turkishe1
turki2a	Turkey	2	turkish1 turkishe1a
turki34	Turkey	34	turkishe14 turkish13 turkishn6 turkishs1
turki3	Turkey	3	turkishe3
turki4	Turkey	4	turkishn2 turkishs2
turki7	Turkey	7	turkishn3 turkish2 kurd1 turkishe1
turki1	Turkey	1	turkishs1
turki17	Turkey	17	turkishs11 turkishn5 turkish1
abhka16	WestCaucasus	16	abhkasian15 georgian1
adyge13	WestCaucasus	13	adygei12 turkishe1
adyge6	WestCaucasus	6	adygei4 balkar1 turkish1
balka16	WestCaucasus	16	balkar16
balka5	WestCaucasus	5	balkar2 abhkasian1 adygei1 georgian1
georg20	WestCaucasus	20	georgian16 abhkasian4
georg2	WestCaucasus	2	georgian2
north15	WestCaucasus	15	northossetian15

[†] Turkish individuals in this cluster likely have ancestors that recently moved to Turkey from Bulgaria or Romania and are most probably of Bulgarian / Romanian origin

Table S4. The final results of the GLOBETROTTER analysis on 82 Eurasian Clusters. $Analysis$ refers to whether the main or masked analysis was used to produce the final result. Admixture P-values are based on 100 bootstrap replicates of the NULL procedure. Our resulting inference, res can be: 1D (two admixing sources at a single date); 1MW (multiple admixing sources at a single date); 2D (admixture at multiple dates); NA (no-admixture); U (uncertain). max(R_1) refers to the R^2 goodness-of-fit for a single date of admixture, taking the maximum value across all inferred coancestry curves. FQ_1 is the fit of a single admixture event (i.e. the first principal component, reflecting admixture involving two sources) and FQ_2 is the fit of the first two principal components capturing the admixture event(s) (the second component might be thought of as capturing a second, less strongly-signalled event. M is the additional R^2 explained by adding a second date versus assuming only a single date of admixture; we use values above 0.35 to infer multiple dates (although see Supplementary Text for details). As well as the final result, for each event we show the inferred dates, αs and best matching sources for 1D, 1MW, and 2D inferences. Inferred dates are in years(+ 95% CI; B=BCE, otherwise CE); the proportion of admixture from the minority source (source 1) is represented by α. Date confidence intervals are based on 100 bootstrap replicates of the date inference (see Supplementary Experimental Procedures for details)

Cluster	Analysis	P	res	max(R_1)	FQ_1	FQ_2	M	$1D$	$1D$(CI)	$1D\alpha1$	$1D$ src1	$1D$ src2	$1MW$ α	$1MW$ src3	$1MW$ src4	$2D1$	$2D1$ (CI)	$2D1\alpha$	$2D1$ src1	$2D1$ src2	$2D2$	$2D1$(CI)	$2D2\alpha$	$2D2$ src1	$2D2$ src2
finni3	main	<0.01	1D	0.557	0.995	1	0.12	469	(213B-1011)	0.12	NorthSiberia	germa36													
tajik3	ncauc	<0.01	1D	0.947	0.996	1	0.07	1276	(1135-1388)	0.18	EastAsia	irani10													
daghe4	ecauc	<0.01	1MW	0.853	0.954	0.998	0.06	1239	(823-1442)	0.05	EastAsia	armen27	0.48	armen9	turki34										
nogay2	ncauc	<0.01	2D	0.953	0.999	1	0.40									1761	(1621-1815)	0.25	Mongolia	adyge6	173B	(1541B-478)	0.31	Mongolia	balka5
nogay7a	ncauc	<0.01	1D(2D)	0.97	0.999	1	0.70	1638	(1515-1684)	0.25	Mongolia	balka5													
lezgi3	ecauc	<0.01	1D	0.827	0.993	0.998	0.28	1564	(1001-1758)	0.04	Mongolia	armen27													
kumyk9	ecauc	<0.01	1D	0.98	0.995	0.999	0.28	1524	(1423-1592)	0.08	Mongolia	armen27													
nogay7	ncauc	<0.01	1D(2D)	0.979	0.997	1	0.48	1479	(1360-1564)	0.20	Mongolia	balka5													
adyge6	wcauc	<0.01	1D	0.963	0.99	1	0.26	1463	(1299-1543)	0.08	Mongolia	kumyk9													
kumyk4	ecauc	<0.01	1MW(2D)	0.918	0.942	0.996	0.39	1443	(1235-1580)	0.07	Mongolia	armen27	0.50	north15	armen9										
daghe2	ecauc	<0.01	1D	0.659	0.981	0.998	0.35	1415	(976-1734)	0.08	Mongolia	armen27													
kumyk2	ecauc	<0.01	1D	0.867	0.985	0.993	0.14	1413	(1172-1591)	0.07	Mongolia	armen27													
turkm5	ncauc	<0.01	1D	0.966	0.997	1	0.33	1369	(1226-1508)	0.14	Mongolia	irani10													
turki2a	turki	<0.01	1D	0.898	1	1	0.17	1354	(1146-1500)	0.07	Mongolia	armen27													
adyge13	wcauc	<0.01	1MW(2D)	0.938	0.969	0.996	0.44	1320	(1156-1427)	0.04	Mongolia	turki34	0.36	armen9	kumyk9										
tajik11	ncauc	<0.01	1D(2D)	0.973	0.994	1	0.50	1295	(1152-1374)	0.17	Mongolia	irani10													
daghe10	ecauc	<0.01	1MW	0.89	0.934	0.998	0.33	1292	(940-1536)	0.04	Mongolia	armen27	0.36	armen9	turki34										
turkm11	ncauc	<0.01	1D	0.985	1	1	0.24	1266	(1159-1323)	0.27	Mongolia	irani10													
turki4	turki	<0.01	1D(2D)	0.935	0.985	1	0.45	1236	(986-1329)	0.08	Mongolia	sicil30													
turki17	turki	<0.01	1D	0.988	1	1	0.30	1213	(1096-1272)	0.14	Mongolia	armen27													
armen9	anat	<0.01	1MW(2D)	0.954	0.931	1	0.57	1204	(1021-1307)	0.06	Mongolia	bulga46	0.49	ukrai48	turki7										
lezgi15	ecauc	<0.01	1MW	0.933	0.901	0.998	0.12	1182	(980-1322)	0.04	Mongolia	armen27	0.35	armen9	turki34										
turki34	turki	<0.01	1D	0.991	1	1	0.34	1115	(1016-1151)	0.08	Mongolia	armen27													
chech19	main	<0.01	1D	0.953	0.976	0.999	0.28	1068	(844-1209)	0.11	Mongolia	lezgi15													
turki3	turki	<0.01	1D	0.935	1	1	0.17	1053	(877-1149)	0.10	Mongolia	armen27													
north15	wcauc	<0.01	1D	0.973	0.991	0.999	0.31	1020	(866-1090)	0.08	Mongolia	kumyk9													
lezgi4	ecauc	<0.01	1MW	0.761	0.914	0.993	0.09	992	(529-1282)	0.06	Mongolia	armen27	0.49	turki34	armen9										
russi25	main	<0.01	1D	0.958	0.999	1	0.30	913	(754-1007)	0.10	Mongolia	ukrai48													
balka16	wcauc	<0.01	1D	0.968	0.997	0.999	0.17	901	(748-976)	0.08	Mongolia	armen27													
chuva16	main	<0.01	1D	0.968	1	1	0.33	829	(627-940)	0.22	Mongolia	ukrai48													
mordo13	main	<0.01	1D	0.927	1	1	0.20	792	(564-975)	0.07	Mongolia	ukrai48													
irani10	main	<0.01	1MW	0.942	0.96	0.989	0.30	1221	(1063-1330)	0.09	CentralAsia	turki7	0.34	Levant	turki17										
balka5	wcauc	<0.01	1MW	0.912	0.867	1	0.22	1177	(876-1389)	0.16	CentralAsia	bulga46	0.36	mordo13	armen27										
roman2	main	<0.01	1D	0.815	1	1	0.10	990	(741-1245)	0.34	India	bulga46													
frenc24	main	<0.01	1D	0.777	0.993	0.998	0.18	846	(424-1011)	0.12	Levant	ceu71													
sardi6	sard	<0.01	1D	0.631	0.993	1	0.05	449	(40-786)	0.07	Levant	itali13													
tsi23	italy	<0.01	1D	0.744	0.999	1	0.08	400	(33B-686)	0.29	Levant	frenc24													
itali8	main	<0.01	1D	0.73	0.988	1	0.07	295	(72B-604)	0.34	Levant	itali13													
armen27	anat	<0.01	1MW	0.548	0.958	0.993	0.04	363B	(1085B-382)	0.36	Levant	kumyk9	0.35	georg20	turki7										
sardi9	sard	<0.01	1D	0.74	0.996	1	0.06	356	(214B-736)	0.09	NorthAfricaII	itali13													
irani5	anat	<0.01	1D(2D)	0.955	0.994	1	0.37	1412	(1054-1526)	0.03	EastAfricaBantu	turki7													
sicil30	italy	<0.01	1D(2D)	0.961	0.984	1	0.50	1105	(882-1250)	0.05	WestAfrica	greek19													
spani27	spain	<0.01	1MW(2D)	0.961	0.907	0.999	0.47	1042	(740-1201)	0.07	WestAfrica	frenc24	0.22	basqu24	itali13										
basqu24	main	<0.01	1MW	0.722	0.703	0.997	0.06	886	(283-1162)	0.01	WestAfrica	spani9	0.42	germa36	spani27										
kurd5	anat	<0.01	1D	0.812	0.994	0.999	0.05	872	(312-1069)	0.01	WestAfrica	turki7													
cypri12	main	<0.01	1MW	0.906	0.971	0.999	0.06	427	(107-734)	0.03	WestAfrica	armen27	0.23	hunga23	armen27										
sardi13	sard	<0.01	1D	0.869	0.983	0.999	0.07	36	(471B-374)	0.02	WestAfrica	itali13													
hunga23	main	<0.01	1D	0.888	0.999	1	0.27	1150	(948-1326)	0.43	sicil30	lithu11													
germa36	main	<0.01	1D	0.711	0.995	1	0.07	969	(778-1160)	0.41	lithu11	frenc24													
bulga46	main	<0.01	1D	0.944	1	1	0.22	968	(800-1028)	0.49	armen27	lithu11													
ukrai48	main	<0.01	1D	0.934	0.988	1	0.26	899	(726-1025)	0.17	turki34	lithu11													
croat18	main	<0.01	1D	0.85	1	1	0.03	708	(492-877)	0.34	armen27	lithu11													
greek19	main	<0.01	1D	0.837	0.999	1	0.11	630	(280-781)	0.35	lithu11	cypri12													
norwe17	main	<0.01	1D	0.539	0.998	1	0.06	351	(262B-893)	0.08	mordo13	ceu71													
lithu11	slav	<0.01	1MW	0.331	0.962	0.994	0.01	85	(1240B-623)	0.22	russi25	hunga23	0.41	orcad5	russi25										
spani9	spain	<0.01	1MW	0.725	0.844	0.999	0.08	668	(286-876)	0.19	basqu24	itali13	0.15	Levant	frenc24										
orcad5	orcad	<0.01	1D	0.322	0.998	0.999	0.06	1241	(876-1442)	0.14	sicil30	norwe17													
ceu71	main	<0.01	1MW(2D)	0.81	0.962	0.994	0.61	858	(467-1224)	0.11	sicil30	germa36	0.44	itali13	norwe17										
turki7	turki	<0.01	1MW	0.526	0.965	0.994	0.04	1015	(561-1234)	0.47	armen27	kurd5	0.09	CentralAsia	kurd5										
orcad6	orcad	<0.01	1D	0.282	0.987	0.996	0.05	708	(65B-1421)	0.37	armen9	norwe17													
itali13	italy	<0.01	1D	0.606	0.986	1	0.04	677	(362-989)	0.33	cypri12	frenc24													
tsi70	italy	<0.01	1D	0.901	0.989	1	0.08	241	(16B-417)	0.42	cypri12	ceu71													
georg20	wcauc	<0.01	1D	0.763	0.995	0.998	0.16	1055	(603-1321)	0.17	nogay7	armen27													
abhka16	wcauc	<0.01	1D	0.889	0.996	0.999	0.34	1147	(906-1274)	0.22	nogay7a	armen27													
syria1	main	1	NA	0.876	0.969	1	0.18	1080	(1051-1051)	0.11	Ethiopia	balka16	0.47	balka16	cypri12	1835		0.49	balka16	armen27	1048		0.03	WestAfrica	armen27
turki2	turki	1	NA	0.845	1	1	0.02	958		0.07	Mongolia	armen27	0.28	hunga23	armen27	1038		0.06	Mongolia	armen27	597		0.13	Siberia	cypri12
orcad2a	main	1	NA							1.00	orcad5														
orcad2	main	1	NA							1.00	orcad5														

Continued on next page

Table S4 Continued from previous page

Cluster	Analysis	P	res	max(R_1)	FQ_1	FQ_2	M	$1D$	$1D$(CI)	$1D\alpha1$	$1D$ src1	$1D$ src2	$1MW$ α	$1MW$ src3	$1MW$ src4	$2D1$	$2D1$ (CI)	$2D1\alpha$	$2D1$ src1	$2D1$ src2	$2D2$	$2D1$(CI)	$2D2\alpha$	$2D2$ src1	$2D2$ src2
itali1	italy	1	NA	0.409	0.939	0.996	0.04	1183	(1154-1154)	0.40	sardi13	cypri12	0.17	sardi9	greek19	1350		0.4	sardi13	armen9	162		0.18	NorthAfricaII	spani9
turki1	turki	1	NA	0.959	0.954	1	0.73	1803	(1774-1774)	0.07	WestAfrica	kumyk9	0.23	CentralAsia	cypri12	1870	(1841-1841)	0.07	WestAfrica	kumyk9	1137	(1108-1108)	0.13	Mongolia	cypri12
tsi2	main	$<$0.01	U	0.404	0.928	1	0.04	582	(29-964)	0.14	balka16	tsi23	0.45	hunga23	tsi23	1318		0.24	balka5	tsi23	550		0.1	Levant	tsi23
ceu2a	main	$<$0.01	U	0.484	0.996	0.998	0.04	1010	(423B-1429)	0.10	finni3	ceu71	0.32	frenc24	ceu71	1625		0.46	frenc24	orcad6	1162		0.12	hunga23	ceu71
armen6	main	$<$0.01	U	0.134	0.987	0.998	0.03	143B	(2881B-1326)	0.42	georg20	sicil30	0.41	irani5	greek19	1344		0.31	armen9	armen27	1604B		0.43	abhka16	cypri12
georg2	main	$<$0.01	U	0.622	0.999	1	0.19	1785	(1605-1853)	0.27	hunga23	georg20	0.13	nogay7	georg20	1908		0.28	ukrai48	georg20	763		0.2	mordo13	georg20
tsi2a	main	$<$0.01	U	0.286	0.999	1	0.13	907B	(1946B-565)	0.39	Levant	norwe17	0.37	mordo13	tsi23	1619		0.41	frenc24	tsi23	494B		0.16	Levant	frenc24
tsi4	main	$<$0.01	U	0.357	0.991	0.999	0.11	91	(688B-569)	0.16	Levant	tsi23	0.39	mordo13	tsi23	1580		0.38	mordo13	tsi23	410		0.06	NorthAfricaII	tsi23
tsi3	main	$<$0.01	U	0.277	0.985	0.997	0.03	208	(315B-693)	0.23	Levant	tsi23	0.45	balka5	tsi23	1547		0.42	armen9	tsi23	7B		0.22	Levant	tsi23
armen2	main	$<$0.01	U	0.155	0.917	0.969	0.04	1594	(691-1892)	0.15	Levant	armen27	0.48	armen27	armen27	1892		0.44	armen27	irani5	1649		0.16	Levant	armen27
mordo2	main	$<$0.01	U	0.583	0.924	0.996	0.08	558	(179-843)	0.12	Mongolia	ukrai48	0.30	finni3	mordo13	1804		0.28	finni3	mordo13	804		0.05	Mongolia	mordo13
ceu2	main	0.59	U	0.0523	1	1	0.01	1825	(10949B-1892)	0.06	orcad5	ceu71	0.37	orcad6	ceu71	1869		0.06	orcad5	ceu71	12008B		0.5	bulga46	orcad5
tsi2b	main	$<$0.01	U	0.491	0.993	0.999	0.09	1512	(1133-1670)	0.31	spani27	tsi23	0.04	Levant	tsi23	1679		0.26	spani27	tsi23	1250		0.39	orcad5	tsi70
ceu3	main	$<$0.01	U	0.108	0.936	0.973	0.03	347B	(2002B-1892)	0.32	spani27	finni3	0.48	orcad5	frenc24	1921		0.14	spani27	ceu71	1237B		0.35	sardi9	finni3

Table S5. Results of All GLOBETROTTER Analyses Performed on 82 Eurasian Clusters, Related to Figure 4. Column headings are as in Table S4. All clusters were analyzed once using all clusters with at least five individuals as surrogates (main analysis). Others were also analyzed with bespoke masked analyses, as noted in the main text. The NULL procedure was run for each of these analyses for comparative purposes.

Supplemental Experimental Procedures

S1 Dataset and identification of genetic populations

S1.1 Outlier removal with PLINK IBD and PCA

We used pairwise IBD analysis to remove potentially related individuals from further analysis. Using PLINK, we compared the proportion of SNPs that were IBD for each individual in a population with every other individual from their population using the pi-hat statistic. Evolutionary history, genetic drift, and relative isolation will lead to differences in the background level of IBD in different populations. To take this into account, we chose a variable threshold of exclusion for putatively related individuals. We used the larger value of the 97.5% quantile of population IBD or 0.2, as the threshold for exclusion, and for all pairs above this threshold, dropped the individual with the lower genotyping rate from further analysis. We next used smartpca in the EIGENSOFT package [S1] to perform PCA on the pruned dataset. Individuals were split into broad geographical regions (Europe; Near East; Asia; Africa) and separate runs of the program were performed. Each region contained individuals typed from different studies on different chips, and visual inspection of PC1 v PC2 plots allowed a qualitative assessment of the merging process. For European populations, smartpca was run with no outlier iterations and all individuals were kept for futher analysis; for the remaining three regions, three outlier iterations were performed, with the resulting outliers dropped from the full analysis. The final dataset contained 2,192 individuals from 144 populations typed on 477,812 SNPs (Table S1).

S1.2 Phasing

We used SHAPEITv1 [S2] to phase the data. SHAPEITv1 conditions the underlying hidden Markov model (HMM) from ref. [S3] on all available haplotypes to quickly estimate haplotypic phase from genotype data. We split our dataset (in binary PLINK format) by chromosome and phased all individuals simulataneously, and used the most likely pairs of haplotypes (using the *–output-max* option) for each individual for downstream applications. Based on the numbers of individuals from Europe, Asia and Africa in our dataset and the instructions of SHAPEITv1 available at the time of phasing, we used an estimated effective population size of 13,887. (1189 Asians; Asian Ne: 14,269; 334 Africans; African Ne 17,469; 669 Europeans; European Ne: 11,418.)

S1.3 Visualising broad-scale population structure with ADMIXTURE

We used ADMIXTURE [S4] to describe the genome-wide ancestry of our dataset and by way of comparison of our method to commonly used procedures (Figure S2). We thinned the dataset to remove the effect of LD by removing SNPs whose pairwise correlation across the sample was greater than 0.2. This thinned dataset contained 135,101 SNPs, and ADMIXTURE was run for all values of $K \in [2, ..., 14]$.

S1.4 Rationale for using genomic data to group individuals

The major aim of our paper is to explore the different ancestral contributions to different "groups" of individuals across Western Eurasia. Often, researchers group individuals based on labels associated with geography (e.g. population), or less often a cultural label such as religion, language or life history. Whilst this is generally satisfactory, it is far from perfect. What constitutes a "population" has been, and still is, a topic of much debate [S5]. However, we nevertheless believe that it is important to consider how to split a dataset into constituent groups when investigating human evolutionary history. Take Italy

as an example. Figure S1A is a PCA of Europe with different population labels assigned to individuals, which are each represented by a point, who all fall under the general geographic descriptive label of Italy (HAPMAP TSI individuals are included in the PCA, but are not coloured). From this plot it is clear that Northern and Southern Italians are genetically distinct, which may potentially be due to different ancestries. Sardinia and Sicily are Italian islands, so fall under the geographic population label of Italy, but (as has been known for a long time [S6]) Sardinians are clearly distinct from the rest of Italy, whilst for Sicilians and Southern Italians it is not as clear-cut. Whilst this sort of difference is not necessarily present across all European countries, this example serves to show us that geographic labels can be improved upon. With this in mind, we constructed new groups of individuals based on an objective assessment of genetic similarity alone.

S1.5 Grouping individuals on the basis of genetic similarity

We used an inferential framework for investigating population structure from haplotypic data [S7]. Initially, haplotypic chromosomes are "painted" sequentially using an updated implementation of a model initially introduced by Li & Stephens [S3] and which is exploited by the CHROMOPAINTER package [S7]. The Li & Stephens copying model explicitly relates linkage disequilibrium to the underlying recombination process and CHROMOPAINTER uses an approximate method to reconstruct each "recipient" individual's haplotypic genome as a series of recombination "chunks" from a set of sample "donor" individuals. The aim of this approach is to identify, at each SNP as we move along the genome, the closest relative genome among the members of the donor sample. Because of recombination, the identity of the closest relative will change depending on the admixture history between individual genomes. Even distantly related populations share some genetic ancestry since most human genetic variation is shared, [S8,S9] but the amount of shared ancestry can differ widely. We use the term "painting" here to refer to the application of a different label to each of the donors, such that – conceptually – each donor is represented by a different colour. Donors may be coloured individually, or in groups based on *a priori* defined labels, such as the geographic population that they come from. By recovering the changing identity of the closest ancestor along chromosomes we can understand the varying contributions of different donor groups to a given population, and by understanding the distribution of these chunks we can begin to uncover the historical relationships between groups.

CHROMOPAINTER reconstructs each recipient individual's haplotypes as mosaics of a set of donors and efficiently summarises this ancestry painting in the form of a 'copying vector', which is the total proportion of genome-wide DNA (either by total expected number of shared chunks, or total expected length of chunks) copied from each labelled donor group (i.e. colour). These copying vectors contain a rich summary of the (genome-wide) relationships between individuals, and similarities in these vectors imply shared ancestral history [S7]. We therefore decided to use these copying vectors and fineSTRUCTURE to group individuals based on this measurement of shared ancestral history.

S1.6 Using CHROMOPAINTER to generate a 'coancestry matrix' for fineSTRUCTURE

S1.6.1 Estimating Ne and θ using Expectation-Maximisation

We performed an initial CHROMOPAINTER run to estimate the two nuisance parameters, Ne and θ using the expectation-maximisation (EM) option. (These are not Ne and θ in the traditional sense but refer to parameters from the Li and Stephens model that are used to estimate the recombination rate distribution underlying the model.) We note the process of estimating them here and their values for reproducibility. The EM algorithm iterates over the data to find the local optimum values for these parameters, given the data. Ne is the 'recombination scaling constant' and is directly related to the effective

population size. It is used by CHROMOPAINTER to convert the values of the genetic distance between SNPs (taken from the genetic map) to the population-scaled values for these distances required by the algorithm. We used the human genome build 36 genetic map downloaded from the HAPMAP website (http://hapmap.ncbi.nlm.nih.gov/downloads/recombination/). θ is the per site mutation rate parameter and is used by the CHROMOPAINTER HMM to allow for imperfect copying between haplotypes.

We used CHROMOPAINTER with 10 Expectation-Maximisation (E-M) steps to jointly estimate the program's parameters Ne and θ, repeating this separately for chromosomes 1, 5, 8, 12, 17, 22 and weight-averaging (using centimorgan sizes) the Ne and θ from the final E-M step across the six chromosomes with the following command line:

```
chromopainter -g <infile> -r <recomrates> -f <donorfile> -o <outfile>
    -a 0 0 -s 0 -i 10 -in -iM
```

Due to the exhaustive nature of this estimation, we averaged these values across a subset of populations (Armenian, Bedouin, Bulgarian, German, Mandenka, Mozabite, Palestinian). We used the global values estimated from these populations of 318.8 and 0.0002 for Ne (-n flag) and θ (-M flag) respectively

S1.7 Using the coancestry matrix to identify clusters with fineSTRUCTURE

We then ran CHROMOPAINTER on each chromosome separately for each individual as a recipient, using every other individual (i.e. 2191) as donors with the following command line:

```
chromopainter -g <infile> -r <recomrates> -f <donorfile> -o <outfile>
    -a 0 0 -s 0 -M 0.0002 -n 318.8
```

This generated a 2192 v 2192 matrix of copying vectors that was then passed to fineSTRUCTURE to cluster individuals on the basis of the similarities of these copying vectors. fineSTRUCTURE is a model based Bayesian clustering algorithm that efficiently uses the output of CHROMOPAINTER to identify population structure. Individuals are initially assumed to have independent ancestry proportions (i.e. copying vectors), but because historical relationships among individuals result in correlations in their copying vectors, individuals can be grouped together on the basis of this similarity. At each iteration, a series of splits and merges are performed on random samples of individuals, such that clusters with higher partition probability are kept at the end of each iteration. We ran fineSTRUCTURE for 10 million iterations, sampling every 10,000, using the following command:

```
finestructure -X -Y -x 0 -y 1e7 -z 1e5 <chunkcounts> <mcmcfile>
```

As the aim of this step was to group individuals into major world regions, we checked pairwise coincidence across all 10 million iterations. Pairwise coincidence (i.e. the number of times a pair of individuals are placed in the same cluster) within and across the two runs was high indicating that individuals tended to occur in the same clusters across all 10 million iterations of the algorithm.

We used the fineSTRUCTURE run with the highest posterior probability to produce a tree relating these clusters by running the maximum a posteriori (MAP) state from the initial run and used 10 million iterations of the tree building model and a very large value for the *maxtreestates* (t) option, to ensure that a large number of trees were considered at each iteration, using the following command:

```
finestructure -X -Y -m T -t 1e8 -x 1e6 <chunkcounts> <mcmcfile> <treefile>
```

To find the tree, fineSTRUCTURE starts from the MAP state and successively merges clusters, choosing the merge giving the highest probability for the merge at each step, which results in a bifurcating tree

relating the clusters [S7]. Bipartition uncertainties are produced for the nodes of the tree, which are the proportion of MCMC samples for which all individuals on one side of the split merge with each other prior to merging with any individual from the other side of the split.

S1.8 Identifying donor World Regions using fineSTRUCTURE

We used the full fineSTRUCTURE analysis to group individuals based on their position in the tree (Fig. S1C). We found 301 clusters in total and visually inspected the tree to identify 22 major clades on the tree and successively merged all non-European clusters to reduce the number from 2192 to 22 worldwide clades, containing individuals that broadly matched geographical regions of the world, and that we use to represent the donor genomes of these different regions (Table S2; Fig. S1B).

The purpose of this step was to identify fineSTRUCTURE "continents" (which we term "world regions") to be used for a second run of the algorithm. The processing time of the algorithm is directly related to the number of individuals included in the analysis, so reducing the number of individuals speeds up the analysis. Furthermore, fineSTRUCTURE initially uses a prior that assumes that all individuals are equally distant from each other, which in the case of worldwide populations is likely to be untrue: European populations are more closely related to each other than to African populations, for example. The result is that not all of the substructure is identified in one run. We therefore generated a set of world-regions, which combine all of the copying vectors from the individuals within them to look like (re-weighted) normal individuals but cannot be split and do not contribute to parameter inference, and can thus be considered as copying vectors that contain the average of the individuals within them. They can therefore be included in the algorithm at minimal extra computational cost and exist primarily to provide chunks to (and from) the remaining groups. We additionally use these world regions, together with the Eurasian clusters, throughout the subsequent analysis as our donor (and in the case of the Eurasian clusters recipient) groups in the GLOBETROTTER analysis. Noting that the North African World Region contained the drifted Mozabites together with Tunisians, and Moroccans, we split the North Africa World Region into two, keeping the Mozabites separate from the other North Africans.

S1.9 Identifying the final Eurasian clusters using fineSTRUCTURE

We performed 2 runs of the fineSTRUCTURE algorithm using the 18 non-Eurasian world regions as 'continents' with the 1000 Eurasian individuals belonging to the 5 world regions (Anatolia, SouthCaucasus, NorthCaucasus, SouthEurope, NorthEurope), using the following command:

```
finestructure -X -Y -m T -t 10e6 -x 1e6 -F <world_regions> <chunkcounts>
        <mcmcfile> <treefile>
```

The two runs of fineSTRUCTURE gave very similar results, inferring 82 and 83 clusters. 74 of the clusters were identical, with the remaining clusters containing cases where at most two individuals were swapped between clusters. Given the high similarity between the clusters, we chose the run with the highest posterior probability containing 100 clusters (82 Eurasian plus 18 world regions) as the final groups for the analysis (Table S3). We use these 82 clusters with GLOBETROTTER to assess the presence of admixture across Europe, and subsequently to infer the proportion, timing, and identity of admixture in these groups.

S1.10 Results of the fineSTRUCTURE analysis

Figure S1 shows summary results of the fineSTRUCTURE analysis. The heatmap clearly suggests that different European groups copy different amounts of their genome from different parts of the world.

These differences are subtle however, and are often not much more than a few percent. The aim of our analysis is to use GLOBETROTTER to try to explain these differences and to assess the evidence that genetic admixture may have caused them.

S1.11 Principal components analysis of the CHROMOPAINTER coancestry matrix

As final justification for the use of fineSTRUCTURE clusters we show a visualisation of a principal components of the CHROMOPAINTER chunklengths coancestry matrix (Fig. S4B-D). The PCA is somewhat similar to PCAs based on genotype data [S10,S11] although the inclusion of Caucasus and Turkish individuals causes the plot to differ from the familiar map of Europe. The points are labelled with the same labels as Figure 1 in the main paper (which is also reproduced in the legend). Whilst generally individuals with the same label (i.e. from the same fineSTRUCTURE cluster) appear close to each other on the plot, some clusters are more spread out. The first two (ten) PCs explain 14.4% (35.4%) of the variance in the data suggesting that not all variation described by the clustering is captured by the highest PCs.

S2 Description of ADMIXTURE analysis

We used ADMIXTURE [S4] to initially survey the dataset for evidence of admixture. We performed 10 independent runs of the algorithm on the pruned dataset of 132K SNPs (see Experimental Procedures), with different randomly selected seeds at all values of $K \in [2, ..., 14]$. Multiple runs at the same K were merged with CLUMPP [S12] using 10,000 iterations of the LargeKGreedy option. Figure S2 shows the results of this analysis, with bars showing the averaged admixture proportions from each of the K inferred groups across all individuals from a given cluster or world region. Admixture is largely visible in all clusters (and some of the world regions) at higher values of K.

In particular, West Eurasian populations tend to be some mixture of 3 main ancestral components (dark blue, dark green, light green), with clear examples of admixture from components outside of these main three (for example the light blue 'Middle Eastern' component present in most European groups), with the Caucasus tending to be a mixture of the same components, with a comparatively larger amount of dark green ancestry, compare to the dark blue/light green of Europe.

S3 GLOBETROTTER: description of method to infer admixture

S3.1 The GLOBETROTTER pipeline

We used GLOBETROTTER to characterise admixture in Eurasian populations. This procedure aims to identify and date admixture among clusters in our dataset, as well as identify the admixing groups involved and the proportions of DNA contributed from each group. The analysis presented here follows closely that of Hellenthal et al [S13] with the key difference that we (a) initially use fineSTRUCTURE to group individuals based on genetics alone; and (b) combine non-Eurasian individuals into broad geographic "world regions" as outlined above. We describe some of the testing and validation performed previously in Section S3.2 below.

To run GLOBETROTTER, we must generate a copying vector for each donor group and a set of painted chromosomes for each recipient group.

Using CHROMOPAINTER to generate DONOR copying vectors for the 18 non-Eurasian world regions and 82 Eurasian clusters

To estimate a set of copying vectors (f^i in the notation of Hellenthal et al [S13]) for j donor groups, for each recipient cluster k we used the original coancestry matrix (where we painted all 2192 with every other) and summed the contributions from each individual in each of the $j \neq k \in [1, ..., K]$ non-recipient groups separately (i.e. 18 non-West Eurasian world regions and 82 West Eurasian clusters), producing a $j \neq k \in [1, ..., K]$ element copying vector for each donor group in the analysis. Note that we therefore generate a separate set of copying vectors for each recipient group, describing the amount of copying from each $j \neq k$ donor groups to that recipient group. In practice, this meant that we estimated the mean copying from each of the $j \neq k$ (i.e. 99) donor groups across all individuals in each recipient group to produce a single donor copying-vector for each of the 100 groups in the analysis.

Using CHROMOPAINTER to generate painting samples of the 82 RECIPIENT Eurasian clusters

We calculated cluster-specific values of N_e and θ by performing a 'leave-one-out' procedure where each individual from a given cluster k is allowed to copy from every other individual with the same cluster label and $n_j - 1$ randomly chosen individuals from each donor cluster $j \neq k \in [1, ..., K]$ (n_j is the number of individuals with cluster label j). Because then there are n_j samples to copy from each cluster $j \neq k \in [1, ..., K]$, while only $n_k - 1$ samples to copy from their own cluster (as they cannot be used to paint them-self), we avoid this reduction by 1 causing problems later, by instead removing one individual from each of the other clusters $j \neq k \in [1, ..., K]$ when painting. Thus all individuals in the dataset copy from the same number of individuals from each labelled cluster ensuring that each individual from each cluster copies from the exact same number of individuals from every other cluster label including their own.

Accounting for the fact that each recipient group is now copying from a different (i.e. $j \neq k$) set of donors, we used the cluster-specific values of Ne and θ calculated above, and re-ran CHROMOPAINTER for each of the K (82) Eurasian clusters with all individuals from the other $j \neq k$ clusters and World Regions (99) as donors, this time without allowing individuals to copy from other individuals in the same cluster, and generating 10 painting samples for each recipient cluster.

```
chromopainter -g <infile> -r <recomrates> -f <donorfile> -o <outfile>
              -s 10 -M mu -n Ne
```

S3.2 Characterising admixture events

To test the robustness of the admixture inference, we use the idea that in truly admixed populations, ancestry segments producing "admixture LD" occur within individual genomes, resulting in ancestry LD characteristically decaying within individual genomes much more strongly than when ancestry is measured in different individuals. To construct a test of this based on our method's inference of genetic ancestry, we first generate "across-individual" coancestry curves, by considering CHROMOPAINTER painting samples from different individuals (using the NULL procedure outlined in the GLOBETROTTER manual and here [S13]), and use these to normalise our original coancestry curves. We re-infer admixture using this "NULL" individual, generating 100 date bootstraps, and test to see if the results are different from the non-NULL inference. Specifically, we assess evidence of any admixture, by obtaining an empirical P-value as P = D/101, where D is the number of NULL date bootstraps with a date λ, where $\lambda \leq 1$ or $\lambda \geq 400$, rejecting the null of no admixture only if $P < 0.01$. We also compare how well the modelled date of admixture (i.e. the coancestry curve R^2) changes between the normal and NULL run of the algorithm, and reject the null of no-admixture only when this reduction is less than 1/3. In practice, this meant that we have little confidence in admixture inference in most clusters containing small numbers of individuals (n <4). Admixture in such clusters is defined as uncertain (U) or, when P >0.01, as no-admixture (NA).

A key aspect of GLOBETROTTER is that we do not need to identify *a priori* the admixing source groups: all that is needed is a set of surrogate groups from which to infer admixture events. Moreover, this approach allows us to define source groups as mixtures of these available surrogate groups. We ran GLOBETROTTER under a number of different scenarios, changing the identity of the available donor groups (outlined in full in Supplemental Experimental Procedures Section S4). We note that in general GLOBETROTTER will identify the most recent admixture event.

S3.3 Validation of GLOBETROTTER

We reported the full description of the GLOBETROTTER method, and our extensive testing of its robustness to different conditions in real and simulated data, in Hellenthal et al [S13] and its associated Supplementary Material. We also conducted comparisons of GLOBETROTTER to other available methods, such as ROLLOFF and ADMIXTURE, in that publication. We re-iterate here several key aspects of the validation we performed.

S3.3.1 GLOBETROTTER simulations undertaken by Hellenthal et al 2014

To test GLOBETROTTER under diverse single, complex, and no-admixture scenarios, incorporating many of the complexities (such as unsampled or admixed donor groups) likely to be present in real data, we simulated admixture scenarios involving real (but hidden to our analysis) human populations [S13] and populations generated under a coalescent framework [S14] incorporating inferred [S15–18] past demographic events.

Admixture was simulated between 7 and 160 generations [200 to 4400 years] ago, with admixture fractions 3 to 50% and genetic differentiation (F_{ST}) between the admixing groups varying from 0.018 (similar to Europe versus Central Asia) to 0.185 (similar to West Africa versus Europe). Results are detailed in Hellenthal et al 2014 [S13]. All populations simulated without admixture, including those with long-term migration, showed no admixture evidence (P >0.1). Power to detect admixture (P <0.01) when present was 94%, and 95% of our 95% bootstrapped confidence intervals (CIs) contained the true admixture date, including cases with two distinct incidents of admixture or multiple groups admixing simultaneously.

In our simulations, inferred source accuracy was very high, with, for example, the mixture representation predicting a haplotype composition more correlated to the true, typically unsampled, source

population than to any single sampled population >80% of the time. However, source accuracy was lower for admixing sources contributing only 5% of DNA, with around 40% of such scenarios yielding elevated (>25%) rates of falsely inferring multiple admixture times and/or admixing groups.

Further testing demonstrated robustness of GLOBETROTTER, in simulations and real data, to haplotypic phase inference approach used, inclusion/exclusion of particular chromosomes, genetic map chosen to provide genetic distances, and the presence of population bottlenecks since admixture, whereas GLOBETROTTER admixture dating was improved relative to ROLLOFF [S19].

Even so, there are multiple settings that we believe are challenging for GLOBETROTTER:

1. although the admixing sources need not be sampled—often impossible because of genetic drift, extinction, or later admixture into the sources themselves—source inference is improved when more similar extant groups are sampled, and GLOBETROTTER may miss events where we lack any extant group that can separate sources.

2. sampling of several genetically very similar groups can mask admixture events they share. Similarly, a caveat is that where genuine, recent bidirectional gene flow has occurred, admixture fractions are difficult to define and interpret. However, date estimation is predicted to still be useful, and in real data the majority of our inferred events do not appear to be bidirectional in this manner.

3. even in theory our approach finds it challenging to distinguish distinct continuous "pulses" of admixture and continuous migration over some time frame, because of the difficulty of separating exponential mixtures [S20]. If the time frame were narrow, we expect to infer a single admixture time within the range of migration dates. Where we infer two admixture dates, in particular with the same source groups, the exponential decay signal could also be consistent with more continuous migration, and so we conservatively refer to this as admixture at multiple dates.

4. we only attempt to analyze populations with signals consistent with at most three groups admixing and infer at most two admixture times, and we can provide only less precise inference of sources for the weaker or older admixture signal in these complex cases.

S3.3.2 Other considerations

We note from the above that the presence of admixed donor groups, as is the case in the current analysis should not affect the power of GLOBETROTTER to identify admixture events. In fact, the program is designed to account for this situation by identifying admixture sources as mixtures of the available donor groups.

The variation in the size of the different donor groups in our analysis is large. This may have an effect on the chromosome painting step of the analysis, as larger groups will provide a greater pool of donor individuals for the recipient individuals to copy from. We try to mitigate against this by using fineSTRUCTURE to initially group together individuals who are statistically indistinguishable from each other, from a genetic point of view. The effect of this step is to standardise individuals into groups that are similarly (un-)related to each other. In this situation, individuals will be equally likely to copy from all individuals from a group, irrespective of group size, because they have, at a given position of genome, the similar haplotype to other individuals in their group.

S3.4 Running GLOBETROTTER

We used GLOBETROTTER (v2) as per the author's guidelines, initially using the samples and donor copying vectors described above and using all clusters with more than 5 individuals as surrogates. A full

description of the GLOBETROTTER algorithm is provided in Hellenthal et al 2014 [S13]. A brief description of the use of GLOBETROTTER in the current context is provided in the Experimental Procedures section of the main paper.

We performed several further analyses where we removed different groups from being putative admixture surrogates. Whilst our fineSTRUCTURE analysis generated independent clusters of individuals, it is still the case that Eurasian groups are in fact very similar. Including very closely related groups as surrogates is not recommended as there needs to be sufficient differences between the surrogates and target such that the target can be reconstructed as a mixture of surrogates. Put in a different way, if a surrogate copying vector is so similar to the target copying vector that they are very highly correlated then the inference procedure will not work.

S3.5 Selecting surrogate clusters for use with GLOBETROTTER

When characterising admixture, GLOBETROTTER uses a set of surrogate populations, whose copying vectors are used to describe the composition of the admixing sources. Whilst theoretically any sampled population who has been painted with the same set of donors can be used, we decided to limit the surrogate populations such that two analyses were performed for most clusters:

1. **main analysis** in this analysis, we used all clusters containing at least 5 individuals as surrogate groups. The only exception to this was the finni3 cluster, containing one Norwegian and both of the Finnish individuals included in the analysis, and which we kept in the analysis to include a representative Scandinavian cluster.

2. **masked analyses** using the main analysis surrogate set meant including related groups in the admixture analysis of some of the clusters. For example, with fineSTRUCTURE, we inferred three Sardinian clusters, each of which contained more than five individuals and which were all therefore included as surrogates in the main analysis. In previous work [S13], we showed that masking closely related groups from the analysis can uncover subtle admixture signals.

Practically to achieve this we split the clusters into European and non-European west Eurasian groups and re-ran the inference removing all clusters from the same fineSTRUCTURE clade in the non-European west Eurasian clusters as defined in Figure 1 and Table S3, (i.e. the Caucasus, Anatolia, and Turkey), whilst for European clusters, we re-ran only those clusters where we inferred multiple clusters from the same geographic population. In Tables S4 and S5, the **Analysis** column records whether the main or masked analysis was used and whether the null procedure was additionally used. Note in these analyses, we did not repaint the individuals, but excluded these groups as surrogates. Specifically, we ran the following masked analyses:

1. *slav* masked lithu11 from ukrai48, mask ukrai48 from lithu11

2. *orcad* the four Orcadian clusters were masked as donors from each other

3. Southern Europe

   (a) *italy* we reran the the Italian clusters disallow all other mainland Italian clusters being surrogates

   (b) *sard* we masked the Sardininian groups from each other

   (c) *spani* we masked the other Spanish cluster from both of these clusters

4. *turk* we re-ran GLOBETROTTER for each Turkish cluster disallowing all other Turkish clusters from being surrogates

5. *anat* we re-ran GLOBETROTTER for each Armenian/Iran cluster disallowing all other Anatolian clusters from being surrogates
6. *wca* we re-ran GLOBETROTTER for each West Central Asia cluster disallowing all other North Caucasus clusters from being surrogates
7. *ecauc* we re-ran GLOBETROTTER for each East Caucasus cluster disallowing all other East Caucasus clusters from being surrogates
8. *wcauc* we re-ran GLOBETROTTER for each West Caucasus cluster disallowing all other West Caucasus clusters from being surrogates

S4 Results of GLOBETROTTER admixture analysis on 82 Eurasian Groups

S4.1 Obtaining an admixture result with GLOBETROTTER

For completeness, we include the results of all GLOBETROTTER analyses performed on the dataset as well as the final set of results that we use to inform the Results and Discussion in the main paper. Further information and a version of the program available to download can be found at www.paintmychromosomes.com. In each run of the program, GLOBETROTTER produces one of the following characterisations of admixture:

1. *no admixture* (NA): we generate an admixture P value by running 100 date bootstraps using the null procedure and compute the number of these bootstraps where the inferred data is > 400 and < 1. If the P value ≥ 0.01, we infer no admixture.

2. *uncertain* (U): admixture is detected but difficult to describe (combined fit quality for two events "fit.quality.2events" < 0.985, or R^2 coefficient of determination of the coancestry curves < 0.2)

3. *one-date* (1D): a single date of admixture between two sources (combined fit quality for two events ≥ 0.985; two-date score "maxScore.2events" < 0.35; fit-quality for a single event "fit.quality.1event" ≥ 0.975)

4. *one-date-multiway* (1MW):a single date of admixture between more than two sources (combined fit quality for two events ≥ 0.985; two-date score < 0.35; fit-quality for a single event < 0.975)

5. *multiple-dates* (2D): two (or more) distinct dates of admixture between two or more sources (combined fit quality for two events ≥ 0.985; two-date score ≥ 0.35)

Additionally, for all events where we infer multiple dates, we checked the results of 100 two date bootstraps. In such cases, if the lower bound of the more recent admixture date bootstrap interval was ≤ 3 generations, we switched the final result to either 1D, if the fit quality for the first event < 0.975 or 1MW if ≥ 0.975.

S4.2 Summary of the GLOBETROTTER results tables

To identify our final results used in the paper, we chose the masked analysis in preference to the main analysis, unless the event inferred in the masked analysis was *uncertain*. In such cases we instead used the result from the main analysis. The final results are shown in Table S4. We also provide the full output from all main and masked runs as well as associated runs in Table S5. We also report the results of running GLOBETROTTER with the null procedure for these analyses. In general, the results of these two analyses were qualitatively similar: the effect of removing closely related clusters from the analysis tends to result in the next most similar cluster being chosen as the best matching source.

As noted above, in some cases where the initial GLOBETROTTER results was inferred to be two dates it was necessary to alter the admixture event inference based on the date bootstraps. In these cases the **res** column contains both the original result (in parentheses) as well as the final result used in the paper. In such cases we include this information in the final table for reference.

In general, when reading the GLOBETROTTER results tables one should identify the admixture result, from the **res** column and then identify the set of columns header with this value to find the detailed admixture characteriations

S4.3 Inferring admixture source copying vectors

GLOBETROTTER estimates the proportion that each source contributes to an admixture event α together with the proportions that all donor copying vectors contribute to each source, which we term β. For each admixing source, the βs sum to one. We can thus recreate the genomic identity for each of the the admixing sources on either side of an event in the form of a copying vector. To do so, for each group involved in the mixture of a source, we take their original copying vector and multiply this by β to generate an 'inferred' copying vector for each source involved in an event. Therefore, in addition to the inference of admixing proportions and dates, we are also able to gain insight into the genetic identity of the sources of admixture.

We can use these source copying vectors in a number of ways. We can compare them to the set of donor (in this case, the fineSTRUCTURE cluster) copying vectors to identify the closest matching contemporary group to the admixture source, which we do to find the "best-matching" donor for each admixture source in Tables S4 and S5 and the figures in the main paper. We can also compare them both to each other and to different sets of copying vectors to provide further understanding on the variation present in the dataset. This approach appeals because it allows us to characterise the genetic profile of the sources of admixture back in time, when they are unlikely to be most similar to a single contemporary group, so viewing them as mixtures is a more appropriate method of viewing these events that happened in the past.

In practice, to compute an inferred source copying vector, we use the βs and our original copying vectors (e.g. f^{donor}) and generate the genetic profile of the admixture source simply as the product of the β coefficients and these fs. For example, if an admixture source, f^{source}, is inferred to be made up of 50% East Asia ($\beta_{EastAsia}$=0.5) and 50% Mongolia ($\beta_{Mongolia}$=0.5), then:

$$f^{source} = \beta_{EastAsia} \times f^{EastAsia} + \beta_{Mongolia} \times f^{Mongolia} = 0.5 \times f^{EastAsia} + 0.5 \times f^{Mongolia}$$

More formally, we have mixture coefficients $\beta_1, \beta_2, ..., \beta_K$ corresponding to the mixing coefficients for populations $1 \leq l \leq K$, where $\beta_l > 0$, so:

$$f = \sum_{l=1}^{K} \beta_l f^l$$

For each event we can generate a major and minor source copying vector in this way, which, as before we normalise to sum to 1, and which is of exactly the same form as copying vectors of both present-day individuals and present-day clusters. This also allows us to (a) project the source genetic profiles onto PCA space computed from the present-day individuals, an aspect which we utilise in Figure 4, and (b) estimate the variation present within groups of copying vectors both before and after admixture.

S4.4 Inferring admixture source copying vectors prior to admixture

We show above the procedure for using GLOBETROTTER to infer a genetic profile for the sources of admixture. In the current setting we are interested in characterising the genetic profile of our Eurasian groups prior to admixture. There are two ways to generate such profiles. The first is to generate the major admixture source exactly as described above:

$$f^{PRE} = f^{MAJOR} = \sum_{l=1}^{K} \beta_l^{MAJOR} f^l$$

where β_l^{MAJOR} are the mixture coefficients for the l donor groups involved in the major admixture source

mixture inferred by GLOBETROTTER. However, because we model admixture between a major and minor source mixing at proportion α, an alternative estimate of the pre-admixture source copying vector can be made by "removing" the minor source of admixture from the original (post-admixture) fineSTRUCTURE cluster copying vector:

$$f^{PRE} = f^{POST} - (\alpha \times f^{MINOR})$$

The estimate of f^{POST}, which is in practice the cluster copying vector inferred from the painting, implicitly includes a drift component. As such, our estimates of the pre-admixture source group copying vectors inferred in these two ways should be similar, but are unlikely to be identical. This is because, in the second case, we incorporate an error term into our estimate. That is, our estimate of the pre-admixture source copying vector is in fact:

$$f^{PRE} = f^{POST} - (\alpha \times f^{MINOR}) = f^{MAJOR} + \epsilon$$

The main two sources of the error (ϵ) here are likely to be imperfect characterisation of the admixture process and genetic drift specific to the cluster being considered (f^{POST}), which is unlikely to be well identified in the GLOBETROTTER admixture source inference. At present we are unable to fully model this error, but we are able to account for it when we compare copying vectors to each other.

S4.5 Estimating diversity in West Eurasia before and after admixture

Although, as outlined above, we identified two alternative approaches for estimating the pre-admixture genetic profile of a cluster, we are still interested in attempting to quantify the change in diversity before and after admixture. We can therefore perform an analysis where we compare the two different pre-admixture sources to separate estimates of the current post-admixture West Eurasian diversity. For the 64 clusters where we inferred admixture we computed the total variation distance (TVD; see below) [S21] for all pairs of copying vectors in the the following four groups (for clusters with multiple sources or dates, we only used the sources from the first event):

1. pre-admixture 1: f^{MAJOR} – the major sources of admixture
2. pre-admixture 2: $f^{POST} - \alpha f^{MINOR}$ – the minor source of admixture "removed" from the cluster copying vector
3. post-admixture 1: $(1-\alpha) \times f^{MAJOR} + \alpha \times f^{MINOR}$ – the admixed group inferred by GLOBETROTTER
4. post-admixture 2: f^{POST} – the fineSTRUCTURE cluster copying vector

In order to disregard the additional error (drift) components that will not be characterised well by GLOBETROTTER, we compared (a) the average TVD (pre-admixture1) with TVD (post-admixture1) and (b) TVD (pre-admixture2) with TVD (post-admixture2). We report (a) in Figure 4 and (a) and (b) in Figure S4. In general we see that variation is greater when we consider the fineSTRUCTURE clusters (e.g. Figure S4I and Figure S4J), but that in general there are no large differences between the TVD estimated across copying vectors within the four groups (Figure S4B), with the caveat that there is perhaps a suggestion that the TVD amongst the major sources of admixture is less than in the other groups. Figure S4K shows that when we compare, for each of the 64 groups where we infer admixture, the TVD between the two methods of inferring "pre-admixture" sources (x-axis) and the two methods for inferring "post-admixture" groups, the variation is highly correlated. This suggests that the differences between the two estimation methods are likely to be because of the same error.

S4.5.1 Computing TVD

We used Total Variation Distance (TVD) to compare copying vectors [S21]. As the copying vectors are discrete probability distributions over the same set of donors, TVD is a natural metric for quantifying the difference between them. For a given pair of groups A and B with copyinig vectors describing the copying from i donors, a_i and b_i we can estimate TVD with the following equation:

$$TVD = 0.5 \times \sum_{i=1}^{n}(|a_i - b_i|)$$

S4.6 The landscape of admixture in West Eurasia

To generate panel (A) Figure 4 in the main text, we took the all individuals from each geographic sampling location and assigned ancestry to them based on the results of GLOBETROTTER. The location of the points are shown in Figure S4A. In locations containing individuals from multiple clusters, we averaged these proportions across all individuals within a location to arrive at the final estimates of ancestry for a given location. To generate panel (C) we show arrows from admixture donor groups to recipients, where the admixture donors are sources where the best-matching coying vector is a West Eurasian group. Panel (D) is an analysis that plots the copying vector of each of these West Eurasian sources on a PCA of contemporary West Eurasians.

S5 Additional discussion of results

In the following section we first provide a brief, direct, comparison of admixture events that we infer in Sardinia and the Balkans with previous studies using different methods and datasets. We then discuss the inferred admixture events from our study in more detail. Except in a couple of cases, we restrict the following discussion to the 57 recipient clusters containing at least four individuals.

S5.1 Comparison to previous results

1. Moorjani et al [S19], who use a method based on allele frequency comparisons, and not haplotypes (ROLLOFF), found evidence for sub-Saharan African admixture in Sardinia 71$\pm$28 generations ago, at a proportion of 3%. These are the same Sardinians included in our analysis. In the largest Sardinian (sardi13) cluster in our analysis we infer West African admixture 66 (53-82) generations ago at a proportion of 2%.

2. Ralph and Coop [S9] using a method that infers tracts of ancestry by inferring Identity by Descent (IBD) along chromosomes, showed evidence that individuals from the Balkans have a high number of shared ancestors, with the length of these shared IBD tracts consistent with common ancestry from the Migration Period. We also find admixture events dating to this period in groups from the Balkans, for example in groups from Hungary (hunga23), Bulgaria (bulga46) and Croatia (croat18), in which we infer north east European ancestry flowing into more southerly regions. Ralph and Coop use a different dataset (POPRES) and different methods to produce qualitatively similar results to us.

S5.2 Continuous low level African admixture in the Mediterranean and Anatolia

We infer West African admixture across broad date ranges, but at low admixture proportions (admixture $\alpha < 0.07$; Figs. 2 and S3) in several Mediterranean groups, consistent with a long term movement between sub-Saharan Africa and southern Europe [S22,S23]. Specific West African admixture dating to the Arabic conquest of the Mediterranean [S24] is seen in Spanish (spani27: 1042 (740-1201CE)), Southern Italian and Sicilian (sicil30: 1105 (882-1250CE)), and Basque (basqu24: 886 (283-1162CE)) clusters. Earlier African admixture at low admixture proportion is inferred in the Cypriots (cypri12: 427(107-734CE)), and a Sardinian cluster (sardi13: 36 (458BCE-430CE); $\alpha = 0.02$). This latter event is consistent with the occurrence of A3b2-M13 (0.6%) and E1a-M44 (0.4%) African Y chromosome lineages in Sardinia [S25]. and the dating is more compatible with documented exchanges between the island and *Mauretania Cesariensis* in Roman times (2nd century BCE to 2nd century CE) than later displacements of northern-African males to Sardinia at the time of the Vandals rule (5th century CE) [S24].

Two Iranic clusters show evidence of African admixture, from West Africa in a Kurdish cluster (kurd5: 872CE (312-1069CE)), and from East Africa in an Iranian cluster (irani5: 1412CE (1054CE-1526CE)). The low admixture proportion ($\alpha = 0.01$) in the Kurdish cluster suggests a very subtle event, the dates of which roughly align with the Arabic conquest of the Mediterranean and the increased movement of sub-Saharan African slaves in the region [S13]. Admixture is more recent in the Iranian cluster, and similarly at a low admixture proportion ($\alpha = 0.03$), but the distinct eastern origin of the African ancestry in this group suggests a different route of African admixture into the region, across the Indian Ocean and Arabia. We observe subtly different signatures of admixture in each of the three Sardinian clusters, as already discussed from West Africa, but also from North Africa, specifically Tunisian and Moroccan sources (sardi9: 356 (507BCE-756CE); $\alpha = 0.09$), and also from the Levant source at a

proportion of $\alpha = 0.07$ (sardi6: 449 (11BCE-755CE)). The dates for these events overlap, consistent with African ancestry originating from a number of different sources. Previous admixture analysis of these individuals found evidence for a small amount of sub-Saharan admixture [S19,S26] around 40 generations ago, which we corroborate here, with the additional suggestion of multiple complex African ancestral histories within individuals from the island.

S5.3 A key role for the Levant in the genetic history of the Mediterranean

Early admixture involving source groups most similar to contemporary populations from in and around the Levant (which we define as the World Region containing individuals from Syria, Palestine, Lebanon, Jordan, Saudi, Yemen and Egypt) is seen at high proportions in several clusters from Italy dating to the first half of the first millennium CE, from Southern Italy (itali8: 295CE (72BCE-604CE); $\alpha = 0.34$), Tuscany (tsi23: 400CE(30BCE-686); $\alpha = 0.29$), and Sardinia, as well as in a large cluster from Armenia at an early date (armen27: 363BCE(1085BCE-383CE)). Traces of Phoenician ancestry (1200-300BCE) have been observed using uni-parental markers from populations around the Mediterranean [S27] which, based on the dates, is unlikely to be the source of ancestry we observe here. Instead these events loosely coincide with the formation of the pan-Mediterranean Roman Empire [S24], which may also have allowed increased gene flow from east to west Mediterranean. A significant amount of ancestry ($\alpha = 0.36$) in a large Armenian cluster (armen27) is the result of a complex ancient event involving multiple admixture sources from the Caucasus, Turkey, and the Levant. Armenia was at various times across this period part of Roman, Christian, Parthian and Arabic empires. Whilst it is impossible to identify a particular event that caused the admixture that we see, the proportion, timing and identity of the source groups in this event are consistent with Armenia's geographic and political position at a junction between Europe and west Asia. Similarly, we also infer a complex event involving the Levant, Central Asia and Turkish sources in an Iranian cluster (irani10), although dates for this event are more recent (1221CE (1063-1330CE)) and therefore more consistent with influx on ancestry from the Near East during and after the Mongolian expansions into western Eurasia. We infer more recent Levant admixture in the French (frenc24: 728(424-1011CE)) and in a complex multiway event in a Spanish cluster (spani9: 668 (286-876CE)). The dates and sources of admixture in these cases are consistent with movements of Middle Eastern and North African individuals during the Islamic Conquest of Spain [S24], and suggest a legacy of this key moment in southern European history in the genomes of French as well as Spanish populations.

S5.4 Multiple waves of admixture from east Asia

The Caucasus contain many linguistically diverse but genetically homogeneous populations [S28]. The historical influence of Asia is particularly clear in the Caucasus and Anatolia, where most events involve Mongolian sources and occur after 1000CE (Figs. 2 and S3, Table S4). Two clusters from Turkey (turki34, turki17) show clear evidence of admixture from Mongolia, which is also present in several groups from the steppe east of the Caucasus, in clusters containing individuals from Tajikistan (tajik11: 1295(1152-1374CE)) and Turkmenistan (turkm11: 1266(1159-1323CE); turkm5: 1369 (1226-1508CE)). Dates for these events centre around 1250CE, suggesting further evidence of the large-scale impact of Chinnghid Mongolian nomads in Western Eurasia [S13,S29] in a wide set of populations. The more recent events involving Mongolian sources in Eastern and Northern Caucasus clusters containing Nogay, Kumyk, Lezgin, and Tabasaran individuals centre around 1400-1500CE which may related to the post-Mongol Timurud dynasty which ruled Central Asia and the Caucaus between 1360 and 1425CE [S30].

Among the Caucasus clusters where we do not specifically infer Mongolian admixture, we find evidence of events, often involving multiple admixture sources including Central Asian (Uzbek and Hazara) sources, in clusters containing Turkish (turki7: 1015 (541-1309CE)), Balkasian (balka5: 1177 (876-1389CE)), and Iranian (irani10: 1221 (1063-1330CE)) individuals. The dates for these events, which peak around 750 years ago, together with the non-Mongolian source groups, suggest a role for secondary movement into this area at the time of the Mongolian expansion, but from Asian groups with distinct non-Mongolian ancestry.

A cluster of two individuals from Romania (roman2: 990 (741-1245CE)) have clear evidence of an admixture event involving a source most similar to India. Romany gypsies have been shown to have ancestry from the India subcontinent, which has been dated to an event between 780 and 900 years ago [S31,S32]. Our date of 800 years ago, or 1200CE broadly agrees with these inferences. The identity of these two individuals as Romani is not possible from the data associated with the samples, but if confirmed, shows that in certain settings GLOBETROTTER can produce accurate historical inference even with limited sample sizes.

Among the northern Europeans, the Finnish (finni3) show evidence of an admixture event involving a minority source most similar to contemporary North Siberians (469CE (213BCE-1011CE)). Finns are thought to have originated from the northward migration, and subsequent contact, between Central Europeans and indigenous Scandinavian hunter-gatherers closely related to the Saami [S33]. The Saami are closely related to the individuals that make up the North Siberian world region, and whilst our confidence in this admixture date is low because of the small size of the cluster, the event we see is likely to represent this key period in Finnish history. Within our dataset, only the Finnish, Hungarians and Mordovians speak Finno-Ugric languages, the latter of which we group into two clusters (mordo13: 792 (564-975CE); mordo2: 558 (179-843CE)) and, together with the Russians (russi25: 913(754-1007CE)) and Chuvash (chuva16: 829 (627-940CE)) populations, infer admixture at approximately the same time (500-900CE) involving Mongolian, Central European, and Finnish donors. In a recent analysis that reconstructed the ancestry of Eurasia on the basis of ancient DNA [S34], the ancestry of these groups could not be explained without a putative stream of recent Asian admixture, a scenario which we confirm in our analysis. As such, the Asian admixture in these groups is unlikely to be associated with the Mongolian expansion described above and may instead be related to earlier Turkic movements, involving the Huns and Avars [S29], but separate to the event inferred in the Finnish.

S5.5 Admixture within Europe: the Medieval Migration Period

A separate set of events involves admixture between groups within West Eurasia. As we previously reported [S13], the formation of the Slavic people at around 1000CE had a significant impact on the populations of northern and eastern Europe, a result that is supported by a related but different analysis [S9]. We infer events involving a "Slavic" source (represented here by a cluster of Lithuanians; lithu11) across all Balkan groups in the analysis (Greece, Bulgaria, Romania, Croatia, and Hungary) as well as in a large cluster of Germanic origin (germa36) and a composite cluster of eastern European individuals (ukrai48). Dates for these events mostly overlap, although are older in Croatia and Greece, and appear to concentrate on the end of the first millenium CE (Figure 3), a time known as the European Migration Period, or *Völkerwanderung* [S35]. We additionally infer events during this period in the Spanish (spani9: 668 (286-876CE)), involving Basque- and northern Italian-like sources, in the British (ceu71: 858 (467-1224CE) involving German-, Central and Southern Italian-, and Norwegian-like sources , in the Orcadians (orcad5: 1241 (889-1412CE); orcad6: 708 (94BCE-1399)) involving Norwegian-, Southern Italian-, and Armenian-like sources, in the Norwegians (norwe17: 351 (262BCE-893CE)) involving Mordovian- and British- like sources, in the northern Italians (itali13: 677 (362-989CE)) involving Cypriot- and French-like sources, and in a large cluster of Tuscans (tsi70: 241 (16BCE-417CE)) involv-

ing Cypriot- and British-like sources. Interestingly, these groups contain individuals that are largely from north-western and central European regions with historically attested influences from different groups during the *Völkerwanderung* [S35], suggesting that this period had a further visible effect on the contemporary populations across Northern and Central Europe.

Supplemental References

[S1] Price AL, Patterson NJ, Plenge RM, Weinblatt ME, Shadick NA, et al. (2006) Principal components analysis corrects for stratification in genome-wide association studies. Nat Genet 38: 904–909.

[S2] Delaneau O, Marchini J, Zagury JF (2012) A linear complexity phasing method for thousands of genomes. Nature Methods 9: 179–181.

[S3] Li N, Stephens M (2003) Modeling linkage disequilibrium and identifying recombination hotspots using single-nucleotide polymorphism data. Genetics 165: 2213–2233.

[S4] Alexander DH, Novembre J, Lange K (2009) Fast model-based estimation of ancestry in unrelated individuals. Genome Res 19: 1655 –1664.

[S5] Lawson DJ (2015) Populations in Statistical Genetic Modelling and Inference. In: Kreager P, Winney B, Ulijaszek S, Capelli C, editors, Population in the Human Sciences: Concepts, Models, Evidence, Also available as: eBook.

[S6] Cavalli-Sforza LL, Menozzi P, Piazza A (1994) The history and geography of human genes. Princeton University Press.

[S7] Lawson DJ, Hellenthal G, Myers S, Falush D (2012) Inference of Population Structure using Dense Haplotype Data. PLoS Genet 8: e1002453.

[S8] Consortium TIH (2010) Integrating common and rare genetic variation in diverse human populations. Nature 467: 52–58.

[S9] Ralph P, Coop G (2013) The Geography of Recent Genetic Ancestry across Europe. PLoS Biol 11: e1001555.

[S10] Novembre J, Johnson T, Bryc K, Kutalik Z, Boyko A, et al. (2008) Genes mirror geography within Europe. Nature 456: 98–101.

[S11] Lao O, Lu TT, Nothnagel M, Junge O, Freitag-Wolf S, et al. (2008) Correlation between Genetic and Geographic Structure in Europe. Current Biology 18: 1241–1248.

[S12] Jakobsson M, Rosenberg NA (2007) CLUMPP: a cluster matching and permutation program for dealing with label switching and multimodality in analysis of population structure. Bioinformatics 23: 1801–1806.

[S13] Hellenthal G, Busby GBJ, Band G, Wilson JF, Capelli C, et al. (2014) A Genetic Atlas of Human Admixture History. Science 343: 747–751.

[S14] Chen GK, Marjoram P, Wall JD (2009) Fast and flexible simulation of DNA sequence data. Genome Res 19: 136–142.

[S15] Gronau I, Hubisz MJ, Gulko B, Danko CG, Siepel A (2011) Bayesian inference of ancient human demography from individual genome sequences. Nat Genet 43: 1031–1034.

[S16] Li H, Durbin R (2011) Inference of human population history from individual whole-genome sequences. Nature 475: 493–496.

[S17] Gutenkunst RN, Hernandez RD, Williamson SH, Bustamante CD (2009) Inferring the Joint Demographic History of Multiple Populations from Multidimensional SNP Frequency Data. PLoS Genet 5: e1000695.

[S18] Keinan A, Mullikin JC, Patterson N, Reich D (2007) Measurement of the human allele frequency spectrum demonstrates greater genetic drift in East Asians than in Europeans. Nat Genet 39: 1251–1255.

[S19] Moorjani P, Patterson N, Hirschhorn JN, Keinan A, Hao L, et al. (2011) The History of African Gene Flow into Southern Europeans, Levantines, and Jews. PLoS Genet 7: e1001373.

[S20] Henn BM, Botigué LR, Gravel S, Wang W, Brisbin A, et al. (2012) Genomic Ancestry of North Africans Supports Back-to-Africa Migrations. PLoS Genet 8: e1002397.

[S21] Leslie S, Winney B, Hellenthal G, Davison D, Boumertit A, et al. (2015) The fine-scale genetic structure of the British population. Nature 519: 309–314.

[S22] Auton A, Bryc K, Boyko AR, Lohmueller KE, Novembre J, et al. (2009) Global distribution of genomic diversity underscores rich complex history of continental human populations. Genome Research 19: 795–803.

[S23] Botigué LR, Henn BM, Gravel S, Maples BK, Gignoux CR, et al. (2013) Gene flow from North Africa contributes to differential human genetic diversity in southern Europe. PNAS 110: 11791–11796.

[S24] Roberts J (2007) The New Penguin History of the World. London, UK: Penguin Books, 5th edition.

[S25] Francalacci P, Morelli L, Angius A, Berutti R, Reinier F, et al. (2013) Low-Pass DNA Sequencing of 1200 Sardinians Reconstructs European Y-Chromosome Phylogeny. Science 341: 565–569.

[S26] Loh PR, Lipson M, Patterson N, Moorjani P, Pickrell JK, et al. (2013) Inferring Admixture Histories of Human Populations Using Linkage Disequilibrium. Genetics 193: 1233–1254.

[S27] Zalloua P, Platt D, El Sibai M, Khalife J, Makhoul N, et al. (2008) Identifying Genetic Traces of Historical Expansions: Phoenician Footprints in the Mediterranean. American Journal of Human Genetics 83: 633–642.

[S28] Yunusbayev B, Metspalu M, Järve M, Kutuev I, Rootsi S, et al. (2011) The Caucasus as an asymmetric semipermeable barrier to ancient human migrations. Molecular Biology and Evolution .

[S29] Atwood C P (2004) Encyclopedia of Mongolia and the Mongol Empire. New York, USA: Facts on File, Inc.

[S30] Beckwith CI (2006) Empires of the Silk Road: A History of Central Eurasia from the Bronze Age to the Present. Princeton, US: Princeton University Press.

[S31] Mendizabal I, Lao O, Marigorta U, Wollstein A, Gusmão L, et al. (2012) Reconstructing the Population History of European Romani from Genome-wide Data. Current Biology 22: 2342–2349.

[S32] Moorjani P, Patterson N, Loh PR, Lipson M, Kisfali P, et al. (2013) Reconstructing Roma History from Genome-Wide Data. PLoS ONE 8: e58633.

[S33] Huyghe JR, Fransen E, Hannula S, Van Laer L, Van Eyken E, et al. (2011) A genome-wide analysis of population structure in the Finnish Saami with implications for genetic association studies. Eur J Hum Genet 19: 347–352.

[S34] Lazaridis I, Patterson N, Mittnik A, Renaud G, Mallick S, et al. (2014) Ancient human genomes suggest three ancestral populations for present-day Europeans. Nature 513: 409–413.

[S35] Heather P (2009) Empires and Barbarians: migration, development and the birth of Europe. London, UK: Macmillan.
